# Supplementary material for: Costs of transitioning the livestock sector to net-zero emissions under future climates
Source: Nat Commun. 2025 Apr 23;16:3810. doi: 10.1038/s41467-025-59203-5 (PMC12019546; doi:10.1038/s41467-025-59203-5)
Supplement: Supplementary file 1 — Supplementary Information [file 41467_2025_59203_MOESM1_ESM.pdf]

Supplementary information

Costs of transitioning the livestock sector to net-zero emissions under future climates

Franco Bilotto[1,2,3], Karen Michelle Christie-Whitehead[4], Bill Malcolm[5], Nicoli Barnes[4,6], Brendan Cullen[5], Margaret Ayre[5], Matthew Tom Harrison[1,*]

[1] Tasmanian Institute of Agriculture, University of Tasmania, Newnham, Launceston, TAS, 7248, Australia

[2] AgResearch, Grasslands Research Centre, Tennent Drive, Private Bag 11008, Palmerston North 4442, New Zealand

[3] Department of Global Development, College of Agriculture and Life Sciences, Cornell University, Ithaca, New York, 14850, USA

[4] Tasmanian Institute of Agriculture, University of Tasmania, 4-8 Bass Highway, Burnie, TAS, 7320, Australia

[5] School of Agriculture, Food and Ecosystem Sciences, The University of Melbourne, Parkville, VIC 3010, Australia

[6] IEAC Federation University Australia PO Box 663 Ballarat VIC 3353, Australia

*Corresponding author: matthew.harrison@utas.edu.au

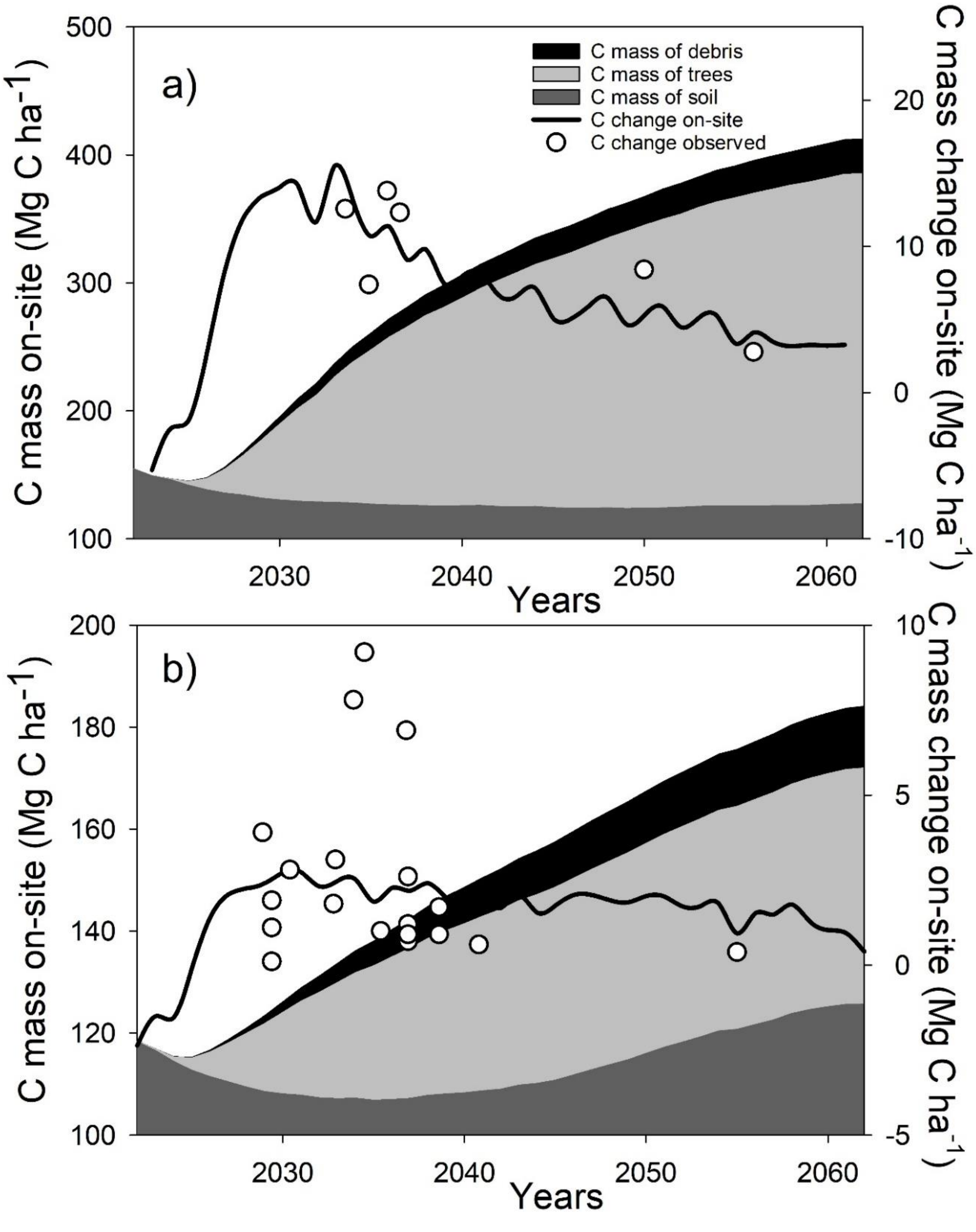

Supplementary figure 1. Carbon stocks and fluxes for the beef and sheep case study farms. Carbon stocks by fraction (trees, debris, and soil; left axes) and annual carbon flux (solid lines, right axes) for tree plantations on the beef (a) and sheep (b) case study farms with annual precipitation >660 mm and between 400 mm and 660 mm, respectively. White circles depict field observations derived from Hobbs et al.[1] and Neumann et al.[2] of carbon sequestered by trees grown in temperate regions of southern Australia (Supplementary tables 16 and 17).

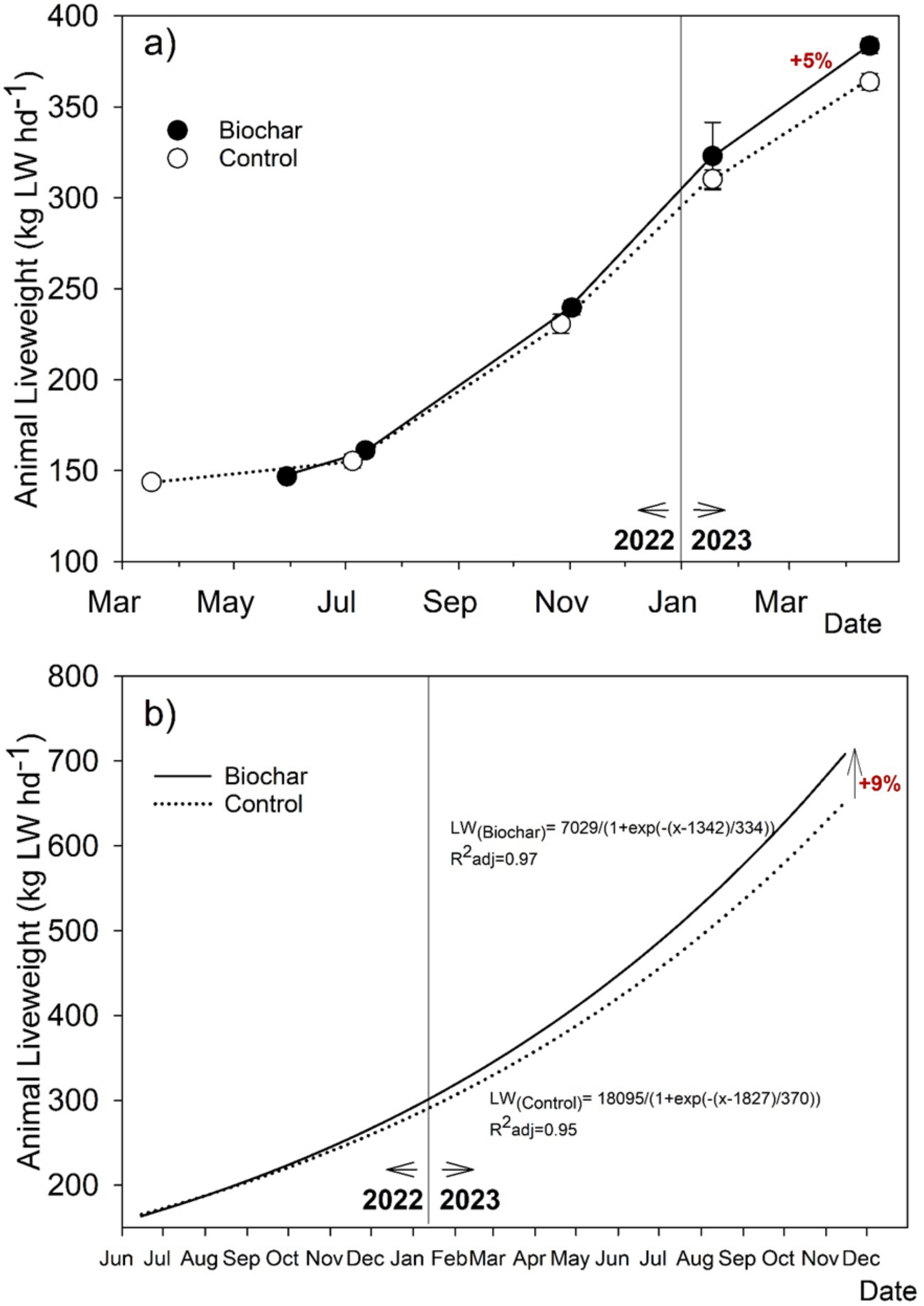

Supplementary figure 2. Measured and modelled cattle liveweight for field experiments conducted at Deloraine, Tasmania, Australia. (a) mean liveweight of steers fed biochar ad libitum (n=50) and no biochar (control, n=65) adapted from Bilotto et al.[3] (b) Regression analysis fitted to data in (a) to project differences between the control and biochar treatment to liveweight values (LW) used in the modelling (x = days). Error bars depict standard error of the mean. Radj = adjusted R^2 value.

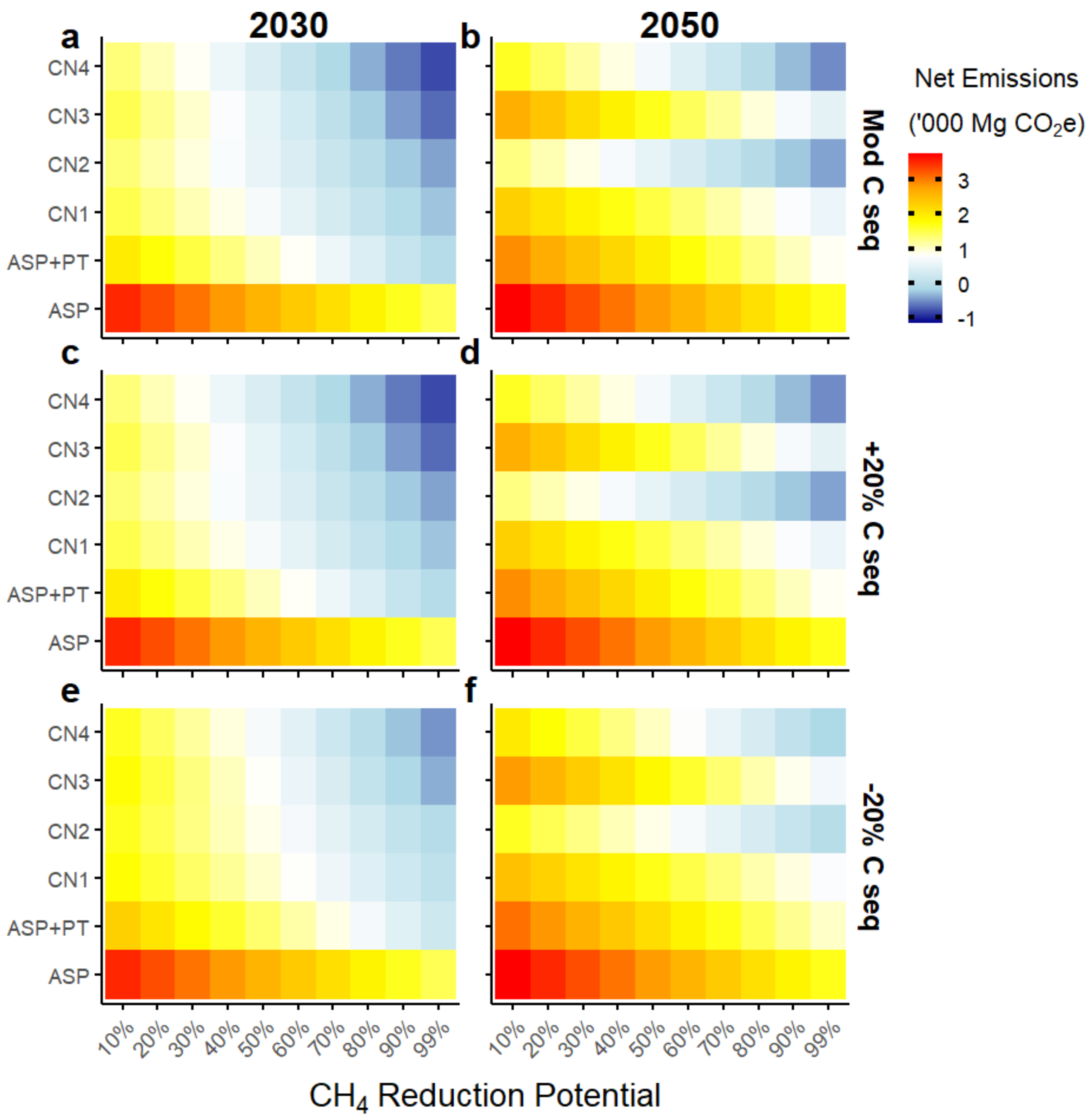

Supplementary figure 3. Sensitivity of net farm GHG emissions to mitigation associated with *Asparagopsis taxiformis* feed supplementation and/or carbon sequestration for a beef production system in 2030 and 2050. Carbon neutral packages were attained by improving animal genetics (CN1 and CN2) or renovating pasture swards with lucerne (CN3 and CN4) in 2030 and 2050 climates. Mod C seq: modelled C sequestration in planting trees; +20 C seq: 20% increase in modelled C sequestration; -20 C seq: 20% decrease in modelled C sequestration; ASP: *A. taxiformis* as livestock feed supplement; ASP+PT: *A. taxiformis* + planting trees (50 ha); CN1: carbon neutral package 1 [*A. taxiformis* + planting trees (50 ha) + transformational feed conversion efficiency]; CN2: carbon neutral package 2 [*A. taxiformis* + planting trees (55 ha in 2030 and 110 ha in 2050) + transformational feed conversion efficiency]; CN3: carbon neutral package 3 [*A. taxiformis* + planting trees (50 ha) + Lucerne]; CN4: carbon neutral package 4 [*A. taxiformis* + planting trees (55 ha in 2030 and 110 ha in 2050) + Lucerne].

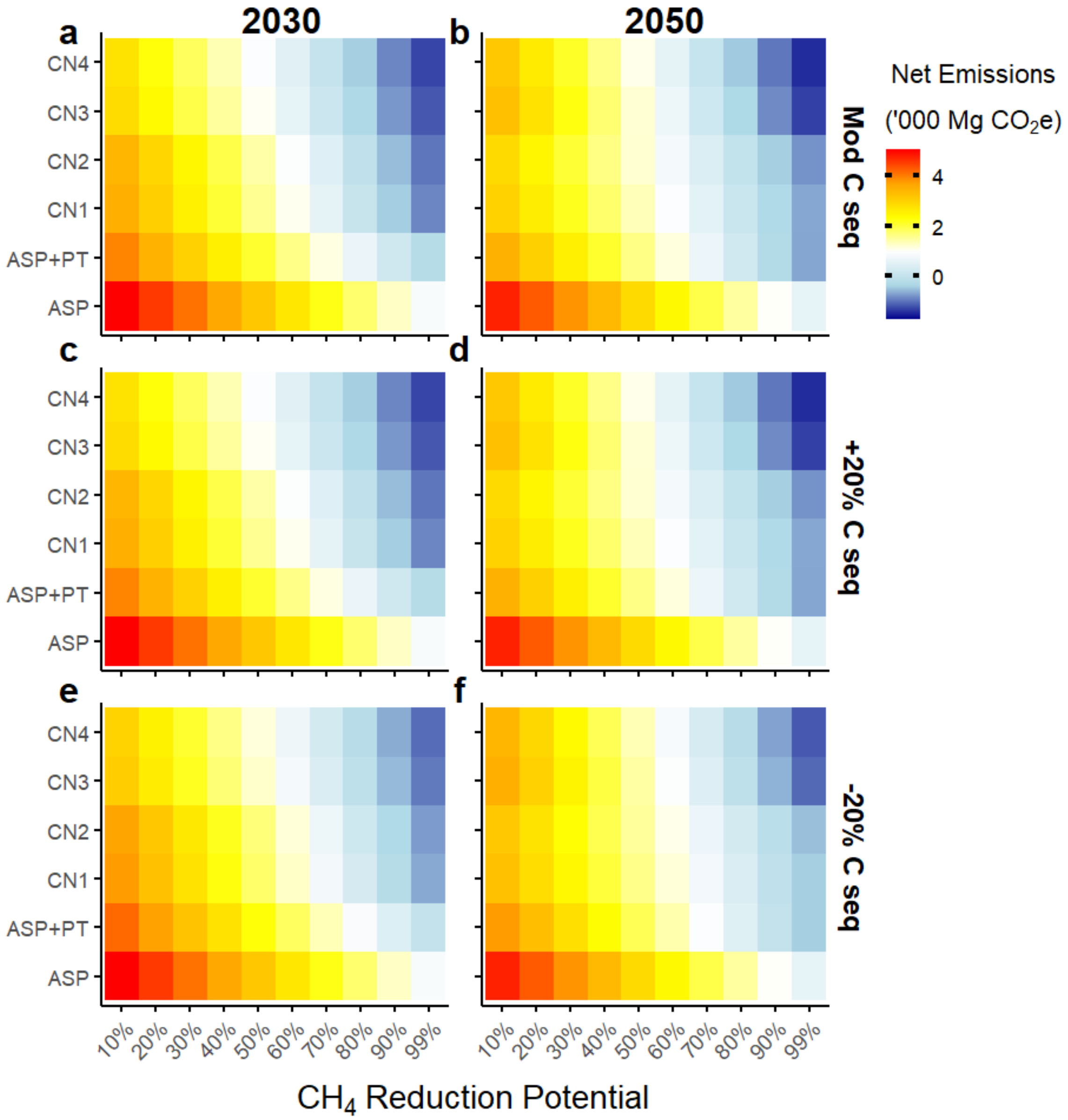

Supplementary figure 4. Sensitivity of net farm GHG emissions to mitigation associated with *Asparagopsis taxiformis* feed supplementation and/or carbon sequestration for a sheep production system in 2030 and 2050. Carbon neutral packages were attained by improving animal genetics (CN1 and CN2) or renovating pasture swards with lucerne (CN3 and CN4) in 2030 and 2050 climates. Mod C seq: modelled C sequestration in planting trees; +20 C seq: 20% increase in modelled C sequestration; -20 C seq: 20% decrease in modelled C sequestration; ASP: *A. taxiformis* as livestock feed supplement; ASP+PT: *A. taxiformis* + planting trees (200 ha); CN1: carbon neutral package 1 [*A. taxiformis* + planting trees (200 ha) + transformational feed conversion efficiency]; CN2: carbon neutral package 2 [*A. taxiformis* + planting trees (220 ha) + transformational feed conversion efficiency]; CN3: carbon neutral package 3 [*A. taxiformis* + planting trees (200 ha) + Lucerne]; CN4: carbon neutral package 4 [*A. taxiformis* + planting trees (220 ha) + Lucerne].

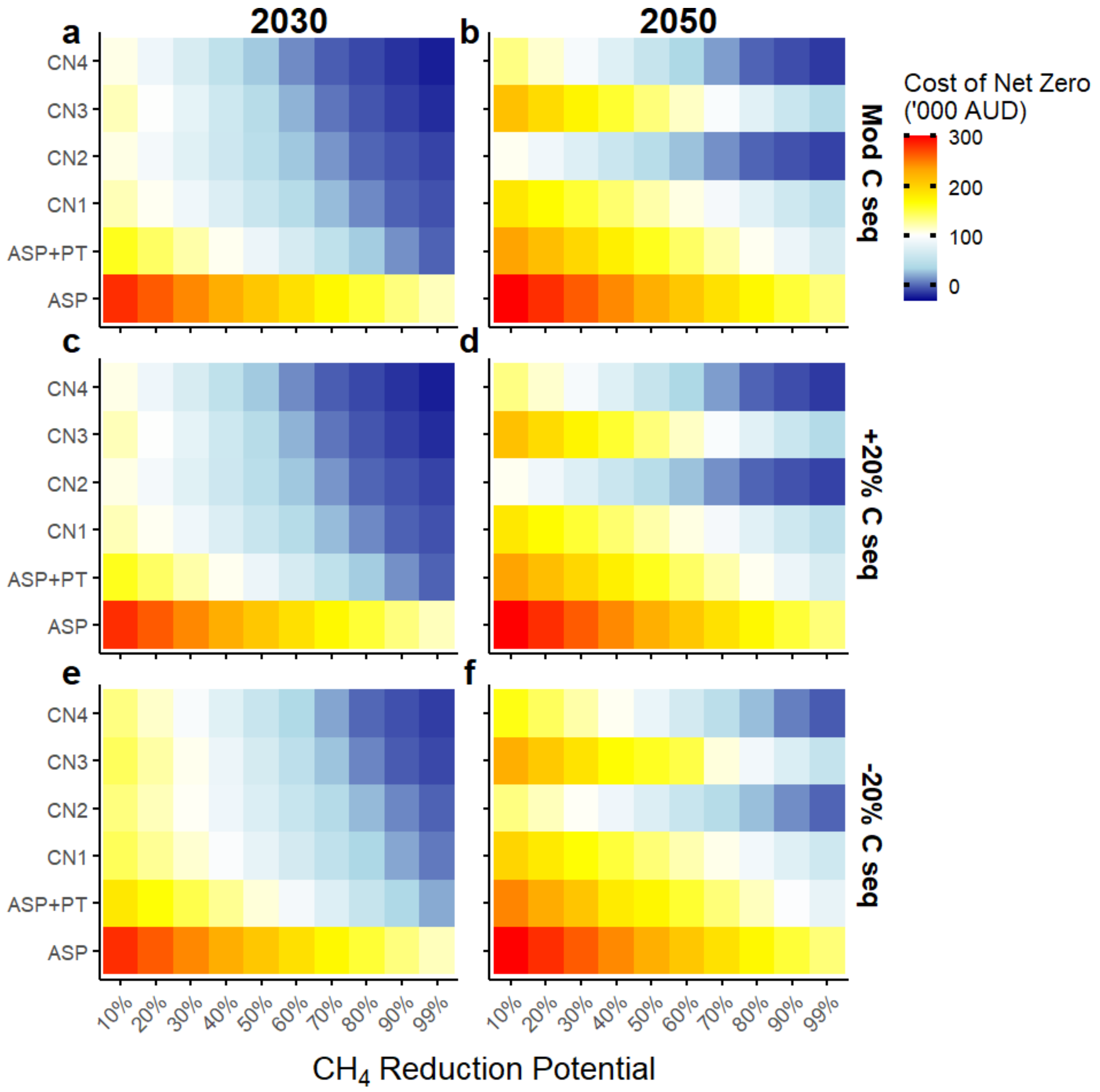

Supplementary figure 5. Cost of transitioning to net-zero emissions associated with CH_4 reduction from feeding *A. taxiformis* and/or carbon sequestration from planting trees for a beef production system in 2030 and 2050. Carbon neutral packages were attained by improving animal genetics (CN1 and CN2) or renovating pasture swards with lucerne (CN3 and CN4) in 2030 and 2050 climates. Mod C seq: modelled C sequestration in planting trees; +20 C seq: 20% increase in modelled C sequestration; -20 C seq: 20% decrease in modelled C sequestration; ASP: *A. taxiformis* as livestock feed supplement; ASP+PT: *A. taxiformis* + planting trees (50 ha); CN1: carbon neutral package 1 [*A. taxiformis* + planting trees (50 ha) + transformational feed conversion efficiency]; CN2: carbon neutral package 2 [*A. taxiformis* + planting trees (55 ha in 2030 and 110 ha in 2050) + transformational feed conversion efficiency]; CN3: carbon neutral package 3 [*A. taxiformis* + planting trees (50 ha) + Lucerne]; CN4: carbon neutral package 4 [*A. taxiformis* + planting trees (55 ha in 2030 and 110 ha in 2050) + Lucerne].

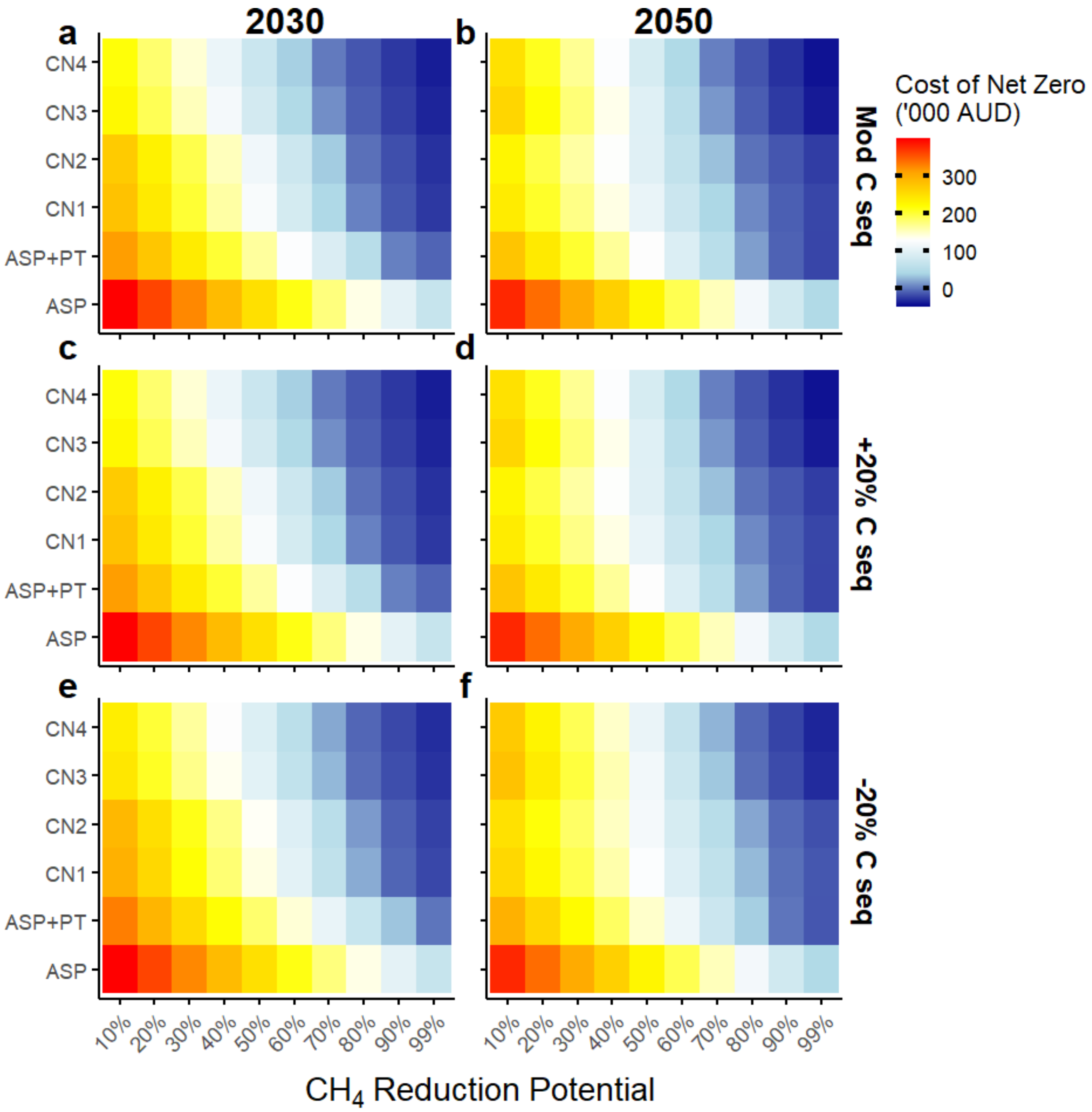

Supplementary figure 6. Cost of transitioning to net-zero emissions associated with CH_4 reduction from feeding *A. taxiformis* and/or carbon sequestration from planting trees for a sheep production system in 2030 and 2050. Carbon neutral packages were attained by improving animal genetics (CN1 and CN2) or renovating pasture swards with lucerne (CN3 and CN4) in 2030 and 2050 climates. Mod C seq: modelled C sequestration in planting trees; +20 C seq: 20% increase in modelled C sequestration; -20 C seq: 20% decrease in modelled C sequestration; ASP: *A. taxiformis* as livestock feed supplement; ASP+PT: A. taxiformis + planting trees 200 ha); CN1: carbon neutral package 1 [*A. taxiformis* + planting trees (200 ha) + transformational feed conversion efficiency]; CN2: carbon neutral package 2 [*A. taxiformis* + planting trees (220 ha) + transformational feed conversion efficiency]; CN3: carbon neutral package 3 [*A. taxiformis* + planting trees (200 ha) + Lucerne]; CN4: carbon neutral package 4 [*A. taxiformis* + planting trees (220 ha) + Lucerne].

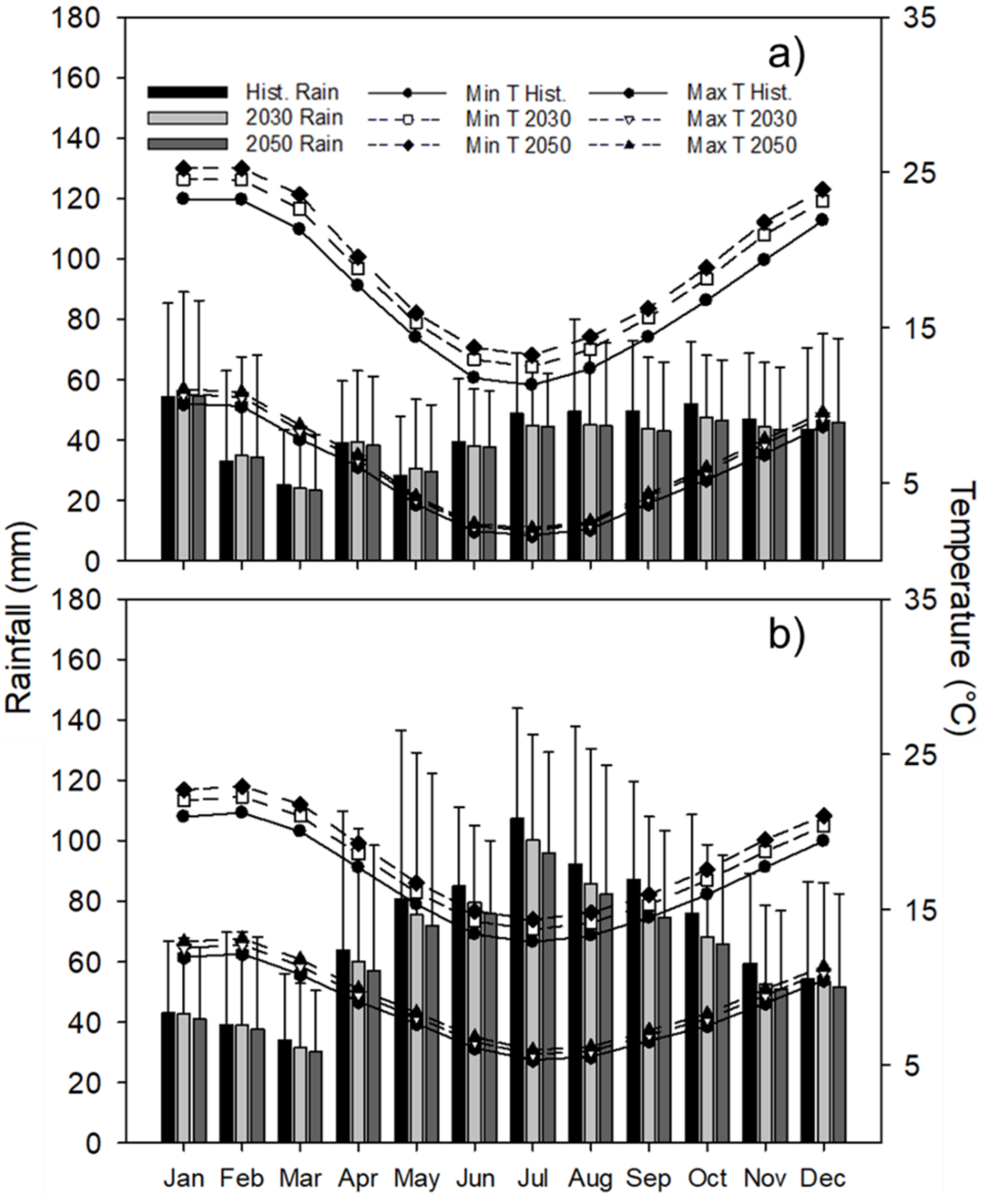

Supplementary figure 7. Monthly average rainfall and temperature for historical, 2030 and 2050 climate horizons for the sheep farm (a) and beef farm (b). Error bars indicate standard deviation. Hist = historical, Min T = minimum average monthly temperature, Max T = maximum average monthly temperature.

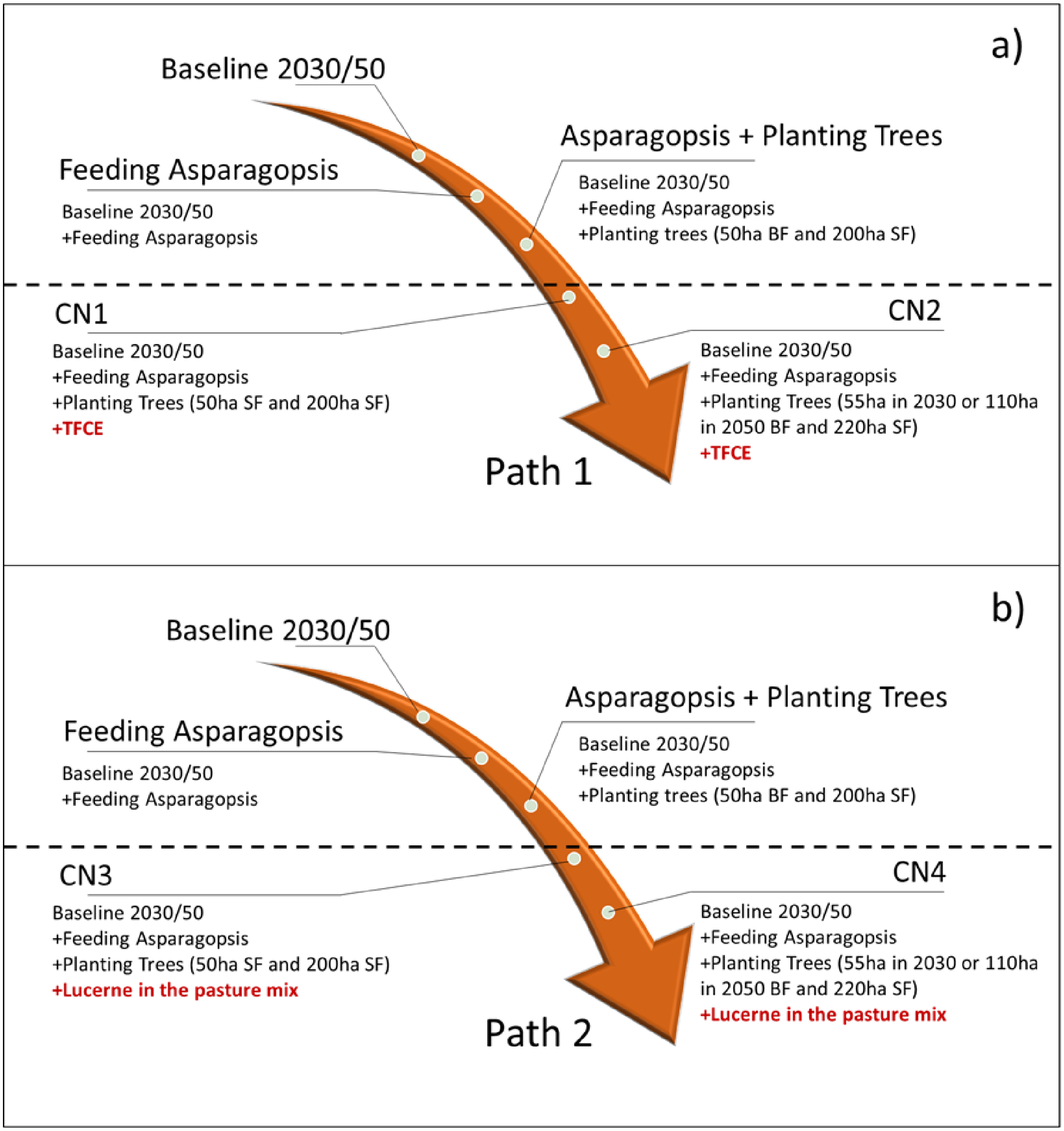

Supplementary figure 8. Co-designed pathways for beef and sheep farms to achieve carbon neutrality under 2030 and 2050 climates. a) Description of pathway 1 including transformational feed conversion efficiency (TFCE), and b) pathway 2 renovating pastures with legumes (lucerne). BF: beef farm. SF: sheep farm. CN1: carbon neutral package 1; CN2: carbon neutral package 2; CN3: carbon neutral package 3; CN4: carbon neutral package 4. TFCE = transformational feed conversion efficiency.

Supplementary Methods

Using GrassGro outputs in RothC to simulate long-term soil carbon accrual in grazed pastures

The Rothamsted Carbon model (RothC) version 26.3[4] was used to simulate dynamic soil organic carbon. RothC has been used extensively to model the impacts of climate and management on SOC stocks around the world[5]. RothC is driven by monthly means of temperature, rainfall and pan evaporation. Monthly average GrassGro outputs were input into RothC including dung (ManureC) and litter (senescent leaves, stems and roots = Plant residueC + RootC) following supplementary equation (1) and (2). Root residue C inputs (RootC) were derived considering the allocation of net primary production (ANP) between plant components [percentage allocated to leaves (Leaf%), stems (Stem%) and seeds (Seed%)] active root length density and proportion of root by layer (0-30 cm and 30-100 cm depth) as shown in supplementary equation (1).

Cumulative monthly pasture litter (litter C, kg DM month^{-1}) was converted to C mass applying a conversion factor of 0.36. The DM fraction was 90% organic matter containing 40% of C[6].

$$\text{LitterC (kg C ha}^{-1}\text{ month}^{-1}) = [\text{Plant residueC (kg C ha}^{-1}\text{ month}^{-1}) + \text{RootC (kg C ha}^{-1}\text{ month}^{-1})] \times 0.9 \times 0.4 \quad \textbf{(1)}$$

Some work allocated only 50% plant residueC and 50% RootC turned over annually and available for soil organic formation[7]. However, if 50% over the remaining 50% in a period of 20-40 years, it makes little difference in the long-term. On the other hand, if LitterC is fully available to the soil organic formation the decomposition rates will impact the C pools instantly. We followed the latter comprehensive and conservative approach to guarantee C accumulation rates. For short-term rotations, we recommend using 50% turnover rate for LitterC.

The cumulative monthly plant residueC was estimated subtracting cumulative pasture intake and haymaking or silage making (mainly in Spring-Summer) over cumulative aboveground net primary production (ANPP) as follows:

$$\text{Plant residueC (kg C ha}^{-1}\text{ month}^{-1}) = [\text{ANPP (kg DM ha}^{-1}\text{ month}^{-1}) - \text{Pasture Intake (kg DM ha}^{-1}\text{ month}^{-1}) - \text{Haymaking or Silage (kg DM ha}^{-1}\text{ month}^{-1})\ ] \times 0.9 \times 0.4 \quad \textbf{(2)}$$

Cumulative monthly RootC was calculated indirectly by using the allocated percentage of assimilates to leaf (Leaf%), stem (Stem%) and seed (Seed%) or a given time *i* following supplementary equation (3):

$$\text{If ANPP} > 0, \text{RootC(kg C ha}^{-1}\text{ month}^{-1}) = [\frac{\text{ANPP (kg DM ha}^{-1}\text{ month}^{-1})}{\text{Leaf\%}_\text{i} + \text{Stem\%}_\text{i} + \text{Seed\%}_\text{i}} - \text{ANPP (kg DM ha}^{-1}\text{ month}^{-1})] \times 0.9 \times 0.4$$

$$\text{If ANPP } \leq 0, \text{RootC(kg C ha}^{-1}\text{ month}^{-1}) = 0 \quad \textbf{(3)}$$

The monthly accumulated amount of RootC (kg C ha^{-1}) at 30 cm and 100 cm depth was based on the proportion of active root by layer.

Cumulative monthly ManureC was estimated as the indigestible fraction of the pasture intake (DMD_{Pi}) or supplement supplied (DMD_{Si}) (mainly in Autumn-Winter) for a given time *i* and was returned as dung to the soil C pool following supplementary equation (4):

$$\text{ManureC (kg C ha}^{-1}\text{ ha}^{-1}\text{ month}^{-1}) = [\text{Pasture Intake (kg DM ha}^{-1}\text{ month}^{-1}) \times (1 - \text{DMD}_{\text{Pi}}) + \text{Supplements (kg DM ha}^{-1}\text{ month}^{-1}) \times (1 - \text{DMD}_{\text{Si}})] \times 0.9 \times 0.4 \quad \textbf{(4)}$$

Accounting for soil carbon flux via biochar in manure

Based on peer-reviewed literature, we used RothC with a sub-model for biochar decomposition[8, 9].

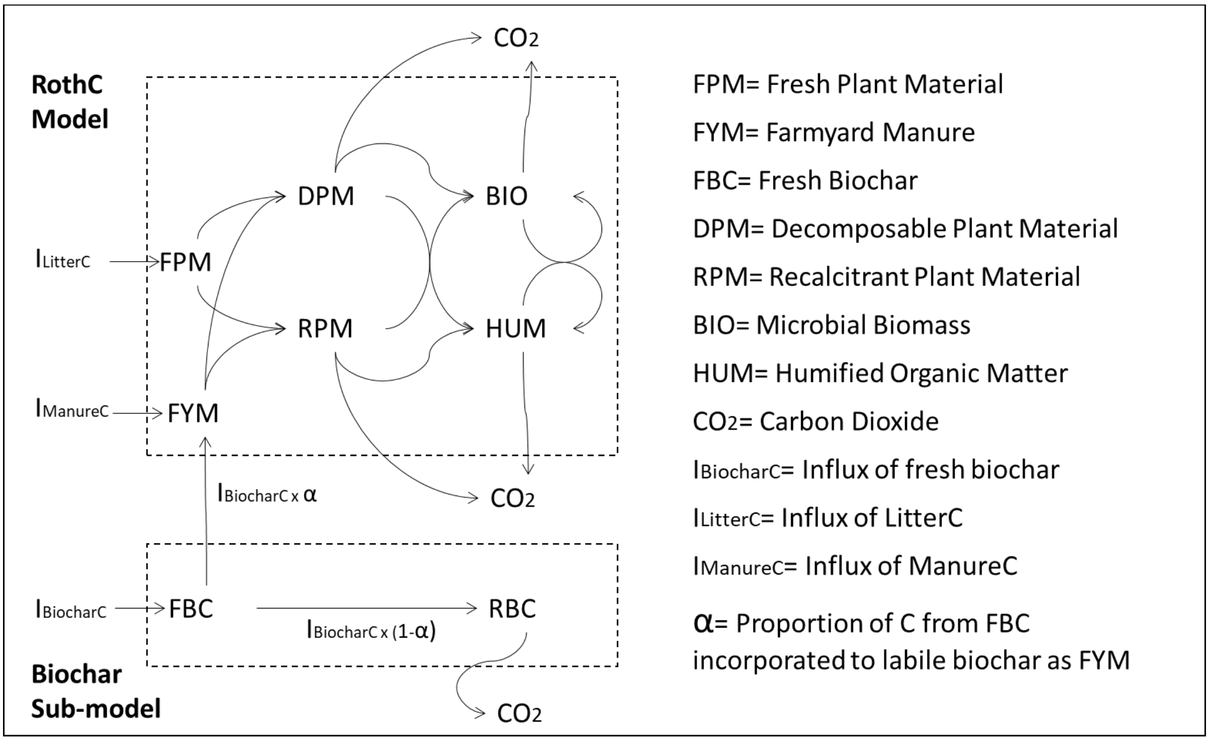

Supplementary figure 9. Conceptual approach for modelling soil organic carbon from biochar carbon enrichment of manure. Biochar sub-model adapted from Lefebvre et al.[8] to RothC for modelling the decomposition of fresh biochar combined with fresh manure and litter.

We targeted total biochar intake rates between 0.5-1.0% (DM basis) from experiments developed under Australian conditions[10]. Assuming that almost 100% of the biochar supplied is excreted as dung with an average carbon concentration of 65%, we modelled cumulative monthly influx C excreted from biochar ($I_{BiocharC}$) following supplementary equation (5):

$$I_{BiocharC}\ (\mathrm{kg\ C\ ha^{-1}\ month^{-1}}) = \mathrm{Biochar\ intake\ (kg\ DM\ ha^{-1}\ month^{-1})} \times 0.65 \quad \textbf{(5)}$$

Here, a proportion, α, of the C in $I_{BiocharC}$ is treated as manure and added to FYM pool in RothC as labile biochar. The remaining fraction of $I_{BiocharC}$ is simulated as recalcitrant material (RBC), thus decomposing very slowly. Given the work developed by Lefebvre et al.[8]; Pulcher et al.[9], we assumed a constant rate of 3% for α added to FYM pool year^{-1} ($I_{BiocharC}$ x 0.03) and 97% as RBC [$I_{BiocharC}$ x (1-0.03)]. For simplicity, the RCB fraction decomposes at a decay rate (*d*CRCB) of 11.9% over 100 years (mean residence time=840 years)[1] as in supplementary equations (6) and (7):

$$\mathrm{dCRCB} = \mathrm{RCB} \times \frac{0.1189}{100} \quad \textbf{(6)}$$

$$\mathrm{RCB_{i+1}} = \left[\mathrm{I_{BiocharC_i}} \times (1 - 0.03)\right] - \mathrm{dCRCB_i} + \mathrm{RCB_i} \quad \textbf{(7)}$$

Diversifying revenue with a grape vine enterprise on the sheep farm

The RRG defined 30 ha of vineyards on the sheep farm to cultivate and harvest Chardonnay and Pinot Noir grapes (representing <1% of total grazable area of the farm). We assume that the animal stock over these 30 ha was absorbed into the remaining farm so no reduction in livestock emissions. Annual inputs included an extra 150 kWh ha^{-1}, 60 litres of diesel ha^{-1}, 35 kg N ha^{-1}, 0.6 kg SSP ha^{-1}, and 10 litres herbicide ha^{-1} [11-13]. Grape GHG emissions were estimated using SB-GAF and were comparative to other studies[14, 15]. Carbon stored in above-ground vines was estimating based on 3,075 vines ha^{-1} (typical planting spacing for the region) and 0.187 kg C ha^{-1} [16]. Changes in below-ground C accumulation in rootstock was assumed to be zero due to unavailable data, while changes in SOC remained the same as per the whole farm due to variable results in the literature[11, 17-19].

Supplementary table 1. Long-term biophysical, environmental, and economic averages for individual incremental, systemic and transformational adaptations in LHF, TCN, ID and CN packages in the high rainfall beef production system in 2030. Hist: historical, B30: baseline farm with no adaptation in 2030, F: increasing soil fertility, DR: increasing 10% root depth, SR: increasing 10% stocking rate, CCD: changing calving date, FCE: increasing 10% feed conversion efficiency, Luc: pasture renovation with lucerne, CH_4 Vac: enteric CH_4 inhibitor vaccine, Planting trees: buying an extra paddock for trees, Extra Farm: buying an extra farm in a different agroclimatic region, Wind: hosting a wind farm by leasing land, Bioc: feeding biochar, Asp: *A. taxiformis* feed supplement, TFCE: increasing 20% feed conversion efficiency.

Variables	Scenarios													
	Hist	B30	F	DR	SR	CCD	FCE	Luc	CH_4 Vac	Planting Trees	Extra Farm	Wind	Bioc	Asp	TFCE
**Livestock System**															
Stocking Rate (DSE ha^{-1} yr^{-1})	24.2	24.4	24.5	24.3	25.9	24.8	23.3	25.1	24.4	24.4	12.6	24.4	24.4	24.4	22.2
Farm Liveweight Production (Mg LW yr^{-1})	287	291	293	291	309	294	307	305	291	291	341.3	291	324	291	319
Protein Production (Mg protein yr^{-1})	52	52	53	52	56	53	55	55	52	52	61	52	58	52	57
Pasture Production (Mg DM ha^{-1} yr^{-1})	20.0	20.5	21.3	20.9	20.2	20.3	20.4	21.8	20.5	20.5	12.3	20.5	20.5	20.5	20.4
Supplementary Feeding (Mg DM ha^{-1} yr^{-1})	0.80	0.78	0.73	0.78	0.86	0.83	0.61	0.32	0.78	0.78	0.56	0.78	0.78	0.78	0.46
Total livestock GHG emissions (Mg CO_2e)	3,864	3,881	3,976	3,889	4,078	3,974	3,638	4,035	3,138	3,881	5,691	3,881	3,652	2,041	3,435
**Soil organic carbon**															
Initial SOC stocks (Mg C ha^{-1}, 1m depth)	235	240	240	240	240	240	240	240	240	240	161	240	240	240	240
Final SOC stocks (Mg C ha^{-1}, 1m depth)	238	241	244	243	240	241	242	248	241	241	163	241	242	241	242
SOC change (Mg C ha^{-1} yr^{-1})	0.14	0.06	0.18	0.12	-0.03	0.03	0.10	0.37	0.06	0.06	0.11	0.06	0.10	0.06	0.10
SOC change (Mg CO_2e ha^{-1} yr^{-1})	0.53	0.21	0.67	0.44	-0.10	0.12	0.37	1.36	0.21	0.21	0.40	0.21	0.35	0.21	0.37
Total SOC change (Mg CO_2e yr^{-1})	301	119	383	252	-58	78	211	773	119	119	523	119	201	119	211
**Forestry system**															
Site C change (Mg C ha^{-1} yr^{-1})	-	-	-	-	-	-	-	-	-	8.3	-	-	-	-	-
Site C change (Mg CO_2e ha^{-1} yr^{-1})	-	-	-	-	-	-	-	-	-	30.5	-	-	-	-	-
Total site C change (Mg CO_2e yr^{-1})	-	-	-	-	-	-	-	-	-	1,527	-	-	-	-	-
**Net GHG emissions**															
Net farm emissions (Mg CO_2e)	3,563	3,762	3,593	3,637	4,135	3,904	3,426	3,262	3,018	2,236	5,168	3,762	3,451	1,921	3,225
Net emission intensity (kg CO_2e kg^{-1} LW produced)	12.4	12.9	12.3	12.5	13.4	13.3	11.2	10.7	10.4	7.7	15.1	12.9	10.6	6.6	10.1
Net emission intensity (kg CO_2e kg^{-1} protein)	69	72	68	70	74	74	62	59	58	43	83	72	60	37	57
**Economics**															

Earnings before interests and taxes ('000 AU$)	487	500	491	502	538	501	585	586	491	494	631	590	589	459	655
Return on Capital (RoC, %)	4.04	4.15	4.08	4.16	4.43	4.15	4.88	4.85	4.07	3.93	3.58	4.89	4.89	3.81	5.49

Supplementary table 2. Long-term biophysical, environmental, and economic averages for individual incremental, systemic and transformational adaptations in LHF, TCN, ID and CN packages in the high rainfall beef production system in 2050. Hist: historical, B50: baseline farm with no adaptation in 2050, F: increasing soil fertility, DR: increasing 10% root depth, SR: increasing 15% stocking rate, CCD: changing calving date, FCE: increasing 10% feed conversion efficiency, Luc: pasture renovation with lucerne, CH_4 Vac: enteric CH_4 inhibitor vaccine, Planting trees: buying an extra paddock for trees, Extra Farm: buying an extra farm in a different agroclimatic region, Wind: hosting a wind farm by leasing land, Bioc: feeding biochar, Asp: *A. taxiformis* feed supplement, TFCE: increasing 30% feed conversion efficiency.

Variables	Scenarios														
	Hist	B50	F	DR	SR	CCD	FCE	Luc	CH_4 Vac	Planting Trees	Extra Farm	Wind	Bioc	Asp	TFCE
**Livestock System**															
Stocking Rate (DSE ha^{-1} yr^{-1})	24.2	24.4	24.5	24.4	25.9	24.9	22.6	25.0	24.4	24.4	12.3	24.4	24.4	24.4	21.6
Farm Liveweight Production (Mg LW yr^{-1})	287	290	294	292	308	295	314	309	290	290	333.6	290	323	290	326
Protein Production (Mg protein yr^{-1})	52	52	53	53	56	53	57	56	52	52	60	52	58	52	59
Pasture Production (Mg DM ha^{-1} yr^{-1})	20.0	20.3	21.1	20.6	20.0	20.3	20.2	19.2	20.3	20.3	12.0	20.3	20.3	20.3	20.3
Supplementary Feeding (Mg DM ha^{-1} yr^{-1})	0.80	0.79	0.74	0.80	0.89	0.84	0.54	0.35	0.79	0.79	0.59	0.79	0.79	0.79	0.39
Total livestock GHG emissions (Mg CO_2e)	3,864	3,890	3,981	3,888	4,073	3,979	3,525	4,136	3,144	3,890	5,635	3,890	3,659	2,045	3,397
**Soil organic carbon**															
Initial SOC stocks (Mg C ha^{-1}, 1m depth)	235	241	244	243	240	241	242	248	241	241	163	241	242	241	242
Final SOC stocks (Mg C ha^{-1}, 1m depth)	238	241	245	242	238	240	242	251	241	241	164	241	242	241	243
SOC change (Mg C ha^{-1} yr^{-1})	0.14	-0.05	0.04	-0.01	-0.10	-0.05	-0.01	0.19	-0.05	-0.05	0.04	-0.05	-0.00	-0.05	0.03
SOC change (Mg CO_2e ha^{-1} yr^{-1})	0.53	-0.18	0.14	-0.04	-0.35	-0.19	-0.03	0.68	-0.18	-0.18	0.16	-0.18	-0.03	-0.18	0.10
Total site C change (Mg CO_2e yr^{-1})	301	-102	80	-23	-196	-110	-20	387	-102	-102	204	-102	-19	-102	58
**Forestry system**															
Site C change (Mg C ha^{-1} yr^{-1})	-	-	-	-	-	-	-	-	-	4.6	-	-	-	-	-
Site C change (Mg CO_2e ha^{-1} yr^{-1})	-	-	-	-	-	-	-	-	-	16.7	-	-	-	-	-
Total site C change (Mg CO_2e yr^{-1})	-	-	-	-	-	-	-	-	-	836	-	-	-	-	-
**Net GHG emissions**															
Net farm emissions (Mg CO_2e)	3,563	3,992	3,902	3,910	4,269	4,088	3,545	3,749	3,246	3,156	5,431	3,992	3,678	2,147	3,340
Net emission intensity (kg CO_2e kg^{-1} LW produced)	12.4	13.8	13.3	13.4	13.9	13.9	11.3	12.1	11.2	10.9	16.3	13.8	11.4	7.4	10.2
Net emission intensity (kg CO_2e kg^{-1} protein)	69	76	74	74	76	77	62	67	62	61	91	76	63	41	57
**Economics**															

Earnings before interests and taxes ('000 AU$)	487	500	494	504	534	505	622	594	491	494	600	560	589	458	694
Return on Capital (RoC, %)	4.04	4.15	4.10	4.18	4.40	4.18	5.19	4.92	4.07	3.93	3.40	4.64	4.89	3.80	5.84

Supplementary table 3. Long-term biophysical, environmental, and economic averages for individual incremental, systemic and transformational adaptations in LHF, TCN, ID and CN packages in the low rainfall sheep production system in 2030. Hist: historical, B30: baseline farm with no adaptation except removal of cattle, DR: increasing rooting depth by 10%, F: increasing soil fertility by 3%, FCE: increasing feed conversion efficiency by 10%, TC: *Talish clover*, LD: Altered lambing date, SR: increased stocking rate, LD/SR: altered lambing date and increased stocking rate, Luc: pasture renovation with lucerne, CH_4 Vac: enteric CH_4 inhibitor vaccine, Planting trees: thickening of non-grazing land with 200 ha of trees, ID: income diversification with vineyard, Bioc: feeding biochar, Asp: *A. taxiformis* feed supplement, TFCE: increasing feed conversion efficiency by 20%.

Variable	Scenario															
	Hist	B30	DR	F	FCE	TC	LD	SR	LD/SR	Luc	CH_4 Vac	Trees	ID	Bioc	Asp	TFCE
**Livestock System**																
Stocking Rate (DSE ha^{-1} yr^{-1})	9.0	8.0	8.0	8.0	7.6	8.0	8.1	9.3	9.5	8.4	8.0	8.0	8.0	8.0	8.0	7.3
Farm Liveweight Production (Mg LW yr^{-1})	370.0	293.6	295.1	294.9	306.9	292.6	309.6	336.0	354.4	322.1	293.6	293.6	293.6	293.6	293.6	319.5
Farm Wool Production (Mg CFW yr^{-1})	71.0	79.0	75.2	77.9	75.5	77.7	78.1	90.8	90.7	82.1	79.0	79.0	79.0	79.0	79.0	72.0
Farm Livestock Production (Mg LW + CFW yr^{-1})	441.0	372.6	370.3	372.8	382.4	370.3	387.7	426.9	445.1	404.1	372.6	372.6	372.6	372.6	372.6	391.5
Protein Production (Mg protein yr^{-1})	137.6	131.9	128.4	131.0	130.7	130.3	133.8	151.3	154.4	140.0	131.9	131.9	133.3	131.9	131.9	129.5
Pasture Production (Mg DM ha^{-1} yr^{-1})	7.2	7.7	7.8	7.9	7.7	7.7	7.5	7.6	7.7	8.3	7.7	7.7	7.7	7.7	7.7	7.7
Supplementary Feeding (Mg DM ha^{-1} yr^{-1})	0.3	0.1	0.1	0.1	0.1	0.1	0.1	0.2	0.2	0.0	0.1	0.1	0.1	0.11	0.11	0.1
Total livestock GHG emissions (Mg CO_2e)	7,037	6,375	6,371	6,413	6,065	6,350	6,505	7,278	7,454	6,804	4,944	6,375	6,375	5,911	2,662	5,834
**Soil organic carbon**																
Initial SOC stocks (Mg C ha^{-1}, 1m depth)	175.0	182.5	182.5	182.5	182.5	182.5	182.5	182.5	182.5	182.5	182.5	182.5	182.5	182.5	182.5	182.5
Final SOC stocks (Mg C ha^{-1}, 1m depth)	179.2	184.1	184.3	185.5	184.9	185.1	184.1	184.3	184.0	185.9	184.1	184.1	184.1	184.9	184.1	184.7
SOC change (Mg C ha^{-1} yr^{-1})	0.21	0.08	0.09	0.15	0.12	0.13	0.08	0.09	0.07	0.17	0.08	0.08	0.08	0.12	0.08	0.11
SOC change (Mg CO_2e ha^{-1} yr^{-1})	0.77	0.29	0.33	0.54	0.43	0.48	0.30	0.33	0.27	0.62	0.29	0.29	0.29	0.44	0.29	0.41
SOC change (Mg CO_2e yr^{-1})	2,425	910	1,043	1,724	1,375	1,537	950	1,049	8,64	1,954	910	910	910	1,388	910	1,298
**Forestry/horticulture system**																
Site C change (Mg C ha^{-1} yr^{-1})	-	-	-	-	-	-	-	-	-	-	-	1.5	0.6	-	-	-
Site C change (Mg CO_2e ha^{-1} yr^{-1})	-	-	-	-	-	-	-	-	-	-	-	5.4	2.1	-	-	-
Farm Grape Production (Mg fresh fruit yr^{-1})	-	-	-	-	-	-	-	-	-	-	-	-	300	-	-	-
Grapes GHG emissions (Mg CO_2e ha^{-1} yr^{-1})	-	-	-	-	-	-	-	-	-	-	-	-	1.1	-	-	-
Total site C change (Mg CO_2e yr^{-1})	-	-	-	-	-	-	-	-	-	-	-	1,073	29	-	-	-

Variable	Scenario															
	Hist	B30	DR	F	FCE	TC	LD	SR	LD/SR	Luc	CH_4 Vac	Trees	ID	Bioc	Asp	TFCE
**Net GHG emissions**																
Net farm emissions (Mg CO_2e)	4,612	5,466	5,328	4,688	4,690	4,813	5,555	6,229	6,591	4,850	4,034	4,393	5,436	4,523	1,752	4,535
Net emission intensity (kg CO_2e kg^{-1} LW produced)	6.0	7.5	7.5	6.4	6.5	6.6	7.5	7.4	7.7	6.2	5.5	6.0	7.5	62	2.4	6.3
Net emission intensity (kg CO_2e kg^{-1} CFW produced)	33.5	41.5	41.5	35.8	35.9	36.9	41.5	41.2	42.7	34.6	30.6	33.3	41.5	34.3	13.3	35.0
Net emission intensity (kg CO_2e kg^{-1} fruit produced)	-	-	-	-	-	-	-	-	-	-	-	-	-0.01	-	-	-
Net emission intensity (kg CO_2e kg^{-1} LW + CFW produced)	10.5	14.7	14.4	12.6	12.3	13.0	14.3	14.6	14.8	12.0	10.8	11.8	14.6	12.1	4.7	11.6
Net emission intensity (kg CO_2e kg^{-1} protein)	33.5	41.5	41.5	35.8	35.9	36.9	41.5	41.2	42.7	34.6	30.6	33.3	40.8	34.3	13.3	35.0
**Economics**																
Earnings before interests and taxes ('000 AU$)	919	1,246	1,210	1,226	1,302	1,224	1,309	1,327	1,408	1,494	1,200	1,240	1,510	1,132	1,158	1,340
Return on Capital (RoC, %)	5.11	6.93	6.73	6.82	7.24	6.81	7.28	7.26	7.70	8.05	6.68	6.78	8.40	6.30	6.44	7.45

Supplementary table 4. Long-term biophysical, environmental, and economic averages for individual incremental, systemic and transformational adaptations in LHF, TCN, ID and CN packages in the low rainfall sheep production system in 2050. Hist: historical, B50: baseline farm with no adaptation except removing cattle, DR: increasing rooting depth by 10%, F: increasing soil fertility by 3%, FCE: increasing feed conversion efficiency by 15%, TC: *Talish clove*r, LD: Altered lambing date, SR: increased stocking rate, LD/SR: altered lambing date and increased stocking rate, Luc: pasture renovation with lucerne, CH_4 Vac: enteric CH_4 inhibitor vaccine, Planting trees: thickening of non-grazing land with 200 ha of trees, ID: income diversification with vineyard, Bioc: feeding biochar, Asp: *A. taxiformis* feed supplement, TFCE: increasing feed conversion efficiency by 30%.

Variable	Scenario															
	Hist	B50	DR	F	FCE	TC	LD	SR	LD/SR	Luc	CH_4 Vac	Trees	ID	Bioc	Asp	TFCE
**Livestock System**																
Stocking Rate (DSE ha^{-1} yr^{-1})	9.0	8.0	8.0	8.0	7.4	8.0	8.2	9.3	9.5	8.4	8.0	8.0	8.0	8.0	8.0	7.0
Farm Liveweight (Mg LW yr^{-1})	370.0	297.2	299.0	299.7	317.0	297.6	314.4	341.8	360.0	328.6	297.2	297.2	297.2	297.2	297.2	334.4
Farm Wool Production (Mg CFW yr^{-1})	71.0	77.6	78.8	78.0	72.6	77.2	77.0	90.4	89.9	81.8	77.6	77.6	77.6	77.6	77.6	68.8
Farm Livestock (Mg LW + CFW yr^{-1})	441.0	374.8	377.8	377.7	389.5	374.8	391.4	432.1	449.9	410.5	374.8	374.8	374.8	374.8	374.8	403.2
Protein Production (Mg protein yr^{-1})	137.6	131.1	132.6	132.0	129.6	130.8	133.6	151.9	154.7	141.0	131.1	131.1	132.5	131.1	131.1	129.0
Pasture Production (Mg DM ha^{-1} yr^{-1})	7.2	7.9	8.0	8.0	7.8	7.9	7.9	7.8	7.8	8.6	7.9	7.9	7.9	7.9	7.9	7.8
Supplementary Feeding (Mg DM ha^{-1} yr^{-1})	0.3	0.1	0.1	0.1	0.1	0.1	0.1	0.2	0.2	0.0	0.1	0.1	0.1	0.12	0.12	0.1
Total livestock GHG emissions (Mg CO_2e)	7,037	6,332	6,375	6,407	5,929	6,330	6,471	7,263	7,425	6,944	4,897	6,332	6,332	5,868	2,622	5,637
**Soil organic carbon**																
Initial SOC stocks (Mg C ha^{-1}, 1m depth)	175.0	184.1	184.3	185.5	184.9	185.1	184.1	184.3	184.0	185.8	184.1	184.1	184.1	184.1	184.1	184.7
Final SOC stocks (Mg C ha^{-1}, 1m depth)	179.2	186.0	185.8	187.7	187.0	187.4	186.0	185.6	185.2	189.2	186.0	186.0	186.0	186.8	186.0	186.4
SOC change (Mg C ha^{-1} yr^{-1})	0.21	0.10	0.07	0.11	0.11	0.11	0.10	0.06	0.06	0.17	0.10	0.10	0.10	0.14	0.10	0.09
SOC change (Mg CO_2e ha^{-1} yr^{-1})	0.77	0.36	0.27	0.42	0.39	0.41	0.35	0.23	0.22	0.61	0.36	0.36	0.36	0.51	0.36	0.31
SOC change (Mg CO_2e yr^{-1})	2,425	1,142	851	1,323	1,221	1,291	1,114	734	705	1,926	1,142	1,142	1,142	1,610	1,142	993
**Forestry/horticulture system**																
Site C change (Mg C ha^{-1} yr^{-1})	-	-	-	-	-	-	-	-	-	-	-	1.7	0.6	-	-	-
Site C change (Mg CO_2e ha^{-1} yr^{-1})	-	-	-	-	-	-	-	-	-	-	-	6.2	2.1	-	-	-
Farm Grape Production (Mg fresh fruit yr^{-1})	-	-	-	-	-	-	-	-	-	-	-	-	300	-	-	-
Grapes GHG emissions (Mg CO_2e ha^{-1} yr^{-1})	-	-	-	-	-	-	-	-	-	-	-	-	1.1	-	-	-
Total site C change (Mg CO_2e yr^{-1})	-	-	-	-	-	-	-	-	-	-	-	1,247	29	-	-	-

Variable	Scenario															
	Hist	B50	DR	F	FCE	TC	LD	SR	LD/SR	Luc	CH_4 Vac	Trees	ID	Bioc	Asp	TFCE
**Net GHG emissions**																
Net farm emissions (Mg CO_2e)	4,612	5,190	5524	5083	4708	5039	5357	6529	6720	5018	3755	3943	5,160	4,258	1,480	4,644
Net emission intensity (kg CO_2e kg^{-1} LW produced)	6.0	7.1	7.5	6.9	6.5	6.9	7.2	7.7	7.8	6.4	5.2	5.4	7.1	5.8	2.0	6.5
Net emission intensity (kg CO_2e kg^{-1} CFW produced)	33.5	39.6	41.6	38.5	36.3	38.5	40.1	43.0	43.4	35.6	28.7	30.1	39.6	32.5	11.3	36.0
Net emission intensity (kg CO_2e kg^{-1} fruit produced)	-	-	-	-	-	-	-	-	-	-	-	-	-0.01	-	-	-
Net emission intensity (kg CO_2e kg^{-1} LW + CFW produced)	10.5	13.8	14.6	13.5	12.1	13.4	13.7	15.1	14.9	12.2	10.0	10.5	13.8	11.4	3.9	11.5
Net emission intensity (kg CO_2e kg^{-1} protein)	33.5	39.6	41.6	38.5	36.3	38.5	40.1	43.0	43.4	35.6	28.7	30.1	39.0	32.5	11.3	36.0
**Economics**																
Earnings before interests and taxes ('000 AU$)	919	1,249	1,278	1,263	1,335	1,254	1,326	1,356	1,437	1,464	1,203	1,228	1,513	1,135	1,161	1,377
Return on Capital (RoC, %)	5.11	6.95	7.11	7.02	7.42	6.97	7.37	7.41	7.85	7.88	6.69	6.72	8.42	6.31	6.46	7.65

Supplementary table 5. Parameters used in modelling the beef cattle farm in north-western (Stanley) and north-eastern (Gladstone) Tasmania, Australia. CFA = cast for age; CP = crude protein; CS = condition score; DM = dry matter; FS = fertility scalar; LW = liveweight; ME = metabolizable energy; MJ = megajoules; RD = root depth.

Herd	Variable	Stanley (Baseline)	Gladstone (main herd) and Stanley (weaners from Gladstone + purchased weaners + purchased yearlings and agisted heifers)
**Main herd (Cow-calf and home-bred young stock herd)**	Area grazed	· 402 ha	· 750 ha
	Livestock numbers	· Stocking rate of 1.1 cows ha^{-1}	· Stocking rate of 0.9 cows ha^{-1}
	Livestock management	· Breed: Angus<br>· Average liveweight at the start of the analysis:<br>-Cows 580 kg LW head^{-1}<br>-Weaners 240 kg LW head^{-1}<br>-Yearlings 425 kg LW head^{-1} steers and 400 kg LW head^{-1} heifers<br>-2-3 years old 650 kg LW head^{-1} steers and 625 kg LW head^{-1} heifers<br>-Calves 50 kg LW/ head^{-1}<br>· Self-replacing herd, replace 11 Feb<br>· Culled cows sold on 10 Feb (6-7 yrs)<br>· Sell excess heifers 30 Sep (26 months) or at 600 kg target LW<br>· Sell steers 15 Sep (25 months) or at 650 kg target LW<br>· Mate 23 Oct, Calving 2 Aug, wean 7 Feb (27 wks)<br>· Age of first joining 1-2 years<br>· 1 bull 25 cows^{-1} (kept for 4 years)<br>· Maint. feed females, when thinnest CS2.5	· Breed: Angus<br>· Average liveweight at the start of the analysis:<br>-Cows 580 kg LW head^{-1}<br>-Weaners 240 kg LW head^{-1}<br>-Calves 50 kg LW head^{-1}<br>· Self-replacing herd, replace 11 Feb<br>· Culled cows sold on 10 Feb (6-7 yrs)<br>· Sell excess heifers 8 Feb (27 wks) or at 200 kg target LW<br>· Sell steers 8 Feb (27 wks) or at 220 kg target LW<br>· Mate 23 Oct, Calving 2 Aug, wean 7 Feb (27 wks)<br>· Age of first joining 1-2 years<br>· 1 bull 25 cows^{-1} (kept for 4 years)<br>· Maint. feed females, when thinnest CS2.5<br>· Maint. feed weaners in paddock when thinnest CS3<br>· Maint. feed 100% hay (DM 85%, ME 11.5 MJ kg DM^{-1}, CP 20%)

	· Maint. feed weaners in paddock when thinnest CS3<br>· Maint. feed 100% hay (DM 85%, ME 11.5 MJ kg DM^{-1}, CP 20%)<br>· Production feeding rule- feedlot cows every year in feedlot and feed 5.5 kg $head^{-1}$ to oldest cows from 1 Jul to 31 Jul<br>· Feed steers in a paddock from 1 Feb to reach 515 kg LW $head^{-1}$ on 31 Aug<br>· Feed heifers in a paddock from 1 Feb to reach 505 kg LW $head^{-1}$ on 15 Sep	· Production feeding rule- feedlot cows every year in feedlot and feed 5.5 kg $head^{-1}$ to oldest cows from 1 Jul to 31 Jul
Livestock genetics	· Default GrassGro parameters for c-k-1, c-k-2, c-k-13 and c-k-14 are 0.5, 0.02, 0.035 and 0.33<br>· Conception rate 95%<br>· Analysis of historical mortality rate from GrassGro 0.5%	· Default GrassGro parameters for c-k-1, c-k-2, c-k-13 and c-k-14 are 0.5, 0.02, 0.035 and 0.33<br>· Conception rate 95%<br>· Analysis of historical mortality rate from GrassGro 0.5%
Pasture types	· Paddock 1 (8 ha), Irrigated Perennial Ryegrass (720 mm RD), Cocksfoot (850 mm RD) and White Clover (500 mm RD)<br>· Paddock 2 (20 ha), Irrigated Lucerne-semi winter active (1200 mm RD), Perennial Ryegrass (720 mm RD)<br>· Paddock 3 (187 ha), Rainfed Perennial Ryegrass (720 mm RD), Cocksfoot (850 mm RD) and Subterranean Clover – Seaton Park (600 mm RD)<br>· Paddock 4 (187 ha), Rainfed Perennial Ryegrass (750 mm RD) and White Clover (500 mm RD)	· Paddock 1 (187 ha), Rainfed Perennial Ryegrass (720 mm RD), Cocksfoot (850 mm RD) and White Clover (500 mm RD)<br>· Paddock 2 (187 ha), White Clover (500 mm RD), Rainfed Perennial Ryegrass (720 mm RD)<br>· Paddock 3 (188 ha), Rainfed Perennial Ryegrass (720 mm RD), Cocksfoot (850 mm RD) and Subterranean Clover – Seaton Park (600 mm RD)<br>· Paddock 4 (188 ha), Rainfed Perennial Ryegrass (750 mm RD) and White Clover (500 mm RD)
Pasture management (note rooting depth in pasture type)	· Irrigate paddock 1 and 2 between 21 Nov and 31 Mar, applying 20 mm and fill to 0.95<br>· Cut paddocks 3 and 4 (whenever DM yield exceeds 5000 kg ha^{-1} between 2 Sep-14 Dec). Proportion gathered 90%. Cutting height 125 mm. Do not cut when DM is below 800 kg ha^{-1}	· Rainfed pastures<br>· Cut paddocks 1 (Years: 1 and 2), 2 (Years: 2 and 3), 3 (Years: 3 and 4) and 4 (Years: 1 and 4) (whenever DM yield exceeds 5000 kg ha^{-1} between 2 Sep-14 Dec).

			Proportion gathered 90%. Cutting height 125 mm. Don't cut when DM is below 800 kg ha^{-1}
	Grazing management	· Cows- From 1 Jul to 30 Jun graze paddocks 3 and 4, withhold 21 days, check every 4 days and move when weight gain margin is > 0.01 kg/day<br>· Heifer Weaners- From 1 Jul to 30 Jun graze paddocks 3 and 4, withhold 21 days, check every 4 days and move when weight gain margin is > 0.01 kg/day<br>· Heifer Yearlings- From 1 Jul to 30 Jun graze paddocks 3 and 4, withhold 21 days, check every 4 days and move when weight gain margin is > 0.01 kg/day<br>· Heifers 2-3 years old- From 1 Jul to 30 Jun graze paddocks 3 and 4, withhold 21 days, check every 4 days and move when weight gain margin is > 0.01 kg/day<br>· Steers Weaners- From 1 Jul to 30 Jun graze paddocks 1, 2, 3 and 4, withhold 21 days, check every 4 days and move when weight gain margin is > 0.01 kg/day<br>· Steers Yearlings- From 1 Jul to 30 Jun graze paddocks 2, 3 and 4, withhold 21 days, check every 4 days and move when weight gain margin is > 0.01 kg/day<br>· Steers 2-3 years old - From 1 Jul to 30 Jun graze paddocks 3 and 4, withhold 21 days, check every 4 days and move when weight gain margin is > 0.01 kg/day	· As per Stanley (mature breeders and self-replacing herd)
	Soils	· All paddocks soil texture defined from Atlas in GrassGro, corresponding to a Northcote Uc2.3 classification[20]<br>· Paddocks 1 and 2 FS 0.87<br>· Paddocks 3 and 4 FS 0.85	· All paddocks soil texture defined from Atlas in GrassGro, corresponding to a Northcote Uc2.33 classification[20]<br>· Paddocks 1 and 2 FS 0.35<br>· Paddocks 3 and 4 FS 0.35
	Tree plantings	· No environmental plantings beyond currently on farm	· No environmental plantings above what currently on farm
**Purchased weaner herd**	Area grazed	· 127 ha	
	Livestock numbers	· Stocking rate of 1.8 steers ha^{-1}	

Livestock management	· Breed: Angus	• Breed: Angus
	· Average liveweight at the start of the analysis:<br>-Weaners 225 kg LW head^{-1}<br>-Yearlings 425 kg LW head^{-1}<br>-2-3 years old 650 kg LW head^{-1}<br>-3-4 years old 700 kg LW head^{-1}<br>· Purchased 1 Feb at 6 mths of age and sold on 15 Sep (25 mths) or at 633 kg LW head^{-1}<br>· Maint. feed mature males and weaners in paddock when thinnest CS2.5.<br>· Maint. feed 100% hay (DM 85%, ME 11.5 MJ kg DM^{-1}, CP 20%)<br>· Feed steers in a paddock from 1 Feb to reach 500 kg LW head^{-1} on 1 Sep	• Average liveweight at the start of the analysis:<br>-Weaners 210 kg LW head^{-1}<br>-Yearlings 415 kg LW head^{-1}<br>-2-3 years old 640 kg LW head^{-1}<br>-3-4 years old 690 kg LW head^{-1}<br>• Purchased 1 Feb at 6 mths of age and sold on 15 Sep (25 mths) or at 610 kg LW head^{-1}<br>• Maint. feed mature males and weaners in paddock when thinnest CS2.5.<br>• Maint. feed 100% hay (DM 85%, ME 11.5 MJ kg DM^{-1}, CP 20%)<br>• Feed steers in a paddock from 1 Feb to reach 490 kg LW head^{-1} on 1 Sep
Livestock genetics	· Default within GrassGro for c-k-1, c-k-2, c-k-13 and c-k-14 are 0.5, 0.02, 0.035 and 0.33	
Pasture types	· Paddock 1 (32 ha), Rainfed Perennial Ryegrass (720 mm RD) and White Clover (500 mm RD)	
	· Paddock 2 (32 ha), Rainfed Perennial Ryegrass (720 mm RD) and White Clover (500 mm RD)<br>· Paddock 3 (31.5 ha), Rainfed Perennial Ryegrass (720 mm RD) and White Clover (500 mm RD)<br>· Paddock 4 (31.5 ha), Rainfed Perennial Ryegrass (720 mm RD) and White Clover (500 mm RD)	
Pasture management	· Reset pasture species as necessary 1 Feb<br>· Cut paddocks 1 (Years: 1 and 4), 2 (Years: 1 and 2), 3 (Years: 2 and 3) and 4 (Years: 3 and 5) (whenever DM yield exceeds 5000 kg ha^{-1} between 2 Sep-14 Dec). Proportion gathered 90%. Cutting height 125 mm. Don't cut when DM is below 800 kg ha^{-1}	

	Grazing management	· Weaners, Yearlings and 2-3 years old- From 1 Jan to 31 Dec graze paddocks 1, 2, 3 and 4, withhold 14 days, check every 7 days and move when weight gain margin is > 0.01 kg day^{-1}
	Soils	· All paddocks soil texture defined from Atlas in GrassGro, corresponding to a Northcote Uc2.3 classification[20]<br>· All paddocks FS 0.85
	Tree plantings	· No environmental plantings above what currently on farm
**Purchased yearlings with agisted heifers**	Area grazed	· 40 ha
	Livestock numbers	· Stocking rate of 3.9 steers ha^{-1}
	Livestock management	· Breed: Angus<br>· Purchased 1 Feb at 16 mths of age (375 kg LW head^{-1}) and sold on 15 Sep (28 mths) or at 545 kg LW head^{-1}<br>· Maint. feed steers in paddock when thinnest CS2.<br>· Maint. feed 100% hay (DM 85%, ME 11.5 MJ kg DM^{-1}, CP 20%)<br>· Feed steers in a paddock from 1 Feb to reach 350 kg LW head^{-1} on 1 Sep
	Livestock genetics	· Default within GrassGro for c-k-1, c-k-2, c-k-13 and c-k-14 are 0.5, 0.02, 0.035 and 0.33
	Pasture types	· Paddock 1 (20 ha), Rainfed Perennial Ryegrass (720 mm RD) and White Clover (500 mm RD)<br>· Paddock 2 (20 ha), Rainfed Perennial Ryegrass (720 mm RD) and White Clover (500 mm RD)
	Pasture management	· No hay cutting
	Grazing management	· Steers (Yearling and 2-3 years old)- From 1 Jan to 31 Dec graze paddocks 1 and 2, withhold 14 days, check every 7 days and move when weight gain margin is > 0.01 kg day^{-1}
	Soils	· All paddocks soil texture defined from Atlas in GrassGro, corresponding to a Northcote Uc2.3 classification[20]<br>· Paddock 1 and 2 FS 0.82

	Tree plantings	· No environmental plantings above what currently on farm
**Weaners from Gladstone to Stanley**	Area grazed	• 402 ha
	Livestock numbers	• Stocking rate of 1.6 steers ha^{-1}
	Livestock management	• Breed: Angus<br>• Average liveweight at the start of the analysis:<br>-Weaners 210 kg LW head-1<br>-Yearlings 415 kg LW head-1<br>-2-3 years old 640 kg LW head-1<br>-3-4 years old 690 kg LW head-1<br>• Purchased 1 Feb at 6 mths of age and sold on 15 Sep (25 mths) or at 610 kg LW head-1<br>• Maint. feed mature males and weaners in paddock when thinnest CS2.5.<br>• Maint. feed 100% hay (DM 85%, ME 11.5 MJ kg DM^{-1}, CP 20%)<br>• Feed steers in a paddock from 1 Feb to reach 490 kg LW $head^{-1}$ on 1 Sep
	Livestock genetics	• Default within GrassGro for c-k-1, c-k-2, c-k-13 and c-k-14 are 0.5, 0.02, 0.035 and 0.33
	Pasture types	• Paddock 1 (32 ha), Rainfed Perennial Ryegrass (720 mm RD) and White Clover (500 mm RD)<br>• Paddock 2 (32 ha), Rainfed Perennial Ryegrass (720 mm RD) and White Clover (500 mm RD)<br>• Paddock 3 (31.5 ha), Rainfed Perennial Ryegrass (720 mm RD) and White Clover (500 mm RD)<br>• Paddock 4 (31.5 ha), Rainfed Perennial Ryegrass (720 mm RD) and White Clover (500 mm RD)
	Pasture management	• Reset pasture species as necessary 1 Feb

	• Cut paddocks 1 (Years: 1 and 4), 2 (Years: 1 and 2), 3 (Years: 2 and 3) and 4 (Years: 3 and 5) (whenever DM yield exceeds 5000 kg ha^{-1} between 2 Sep-14 Dec). Proportion gathered 90%. Cutting height 125 mm. Don't cut when DM is below 800 kg ha^{-1}
Grazing management	• Weaners, Yearlings and 2-3 years old- From 1 Jan to 31 Dec graze paddocks 1, 2, 3 and 4, withhold 14 days, check every 7 days and move when weight gain margin is > 0.01 kg day^{-1}
Soils	• All paddocks soil texture defined from Atlas in GrassGro, corresponding to a Northcote Uc2.3 classification[20]<br>• All paddocks FS 0.85
Tree plantings	• No environmental plantings above what currently on farm

Supplementary table 6. Biophysical, environmental, and economic outcomes associated with pathways to net zero emissions in the high rainfall beef production system in 2030. Hist: historical, Base: baseline farm with no adaptation, LHF: Low Hanging Fruit and TCN: Towards Carbon Neutral. Hist: scenarios simulated with historical climates. Base: impact of future climates. LHF: low-hanging fruit packages. TCN: towards carbon neutrality package. ID: income diversification. Asp: *A. taxiformis*. Asp+PT: *A. taxiformis* + Planting trees 50ha. CN1: carbon neutral package 1 (*A. taxiformis* + planting trees 50ha+ transformational feed conversion efficiency. CN2: carbon neutral package 2 (*A. taxiformis*+ planting trees 55ha + transformational feed conversion efficiency. CN3: carbon neutral package 2 (*A. taxiformis*+ planting trees 50ha + Lucerne). CN4: carbon neutral package 4 (*A. taxiformis*+ planting trees 55ha + Lucerne)

**Variables**	**Scenarios**										
	**Hist**	**Base30**	**LHF**	**TCN**	**ID**	**Asp**	**Asp+PT**	**CN1**	**CN2**	**CN3**	**CN4**
**Livestock System**											
Stocking Rate (DSE ha^{-1} yr^{-1})	24.2	24.4	25.3	25.8	12.6	24.4	24.4	22.2	22.2	25.1	25.1
Farm Liveweight Production (t LW yr^{-1})	287	291	332	344	341.3	291	291	319	319	305	305
Protein Production (t protein yr^{-1})	52	52	60	62	61	52	52	57	57	55	55
Pasture Production (t DM ha^{-1} yr^{-1})	20.0	20.5	21.5	22.6	12.3	20.5	20.5	20.4	20.4	21.8	21.8
Supplementary Feeding (t DM ha^{-1} yr^{-1})	0.80	0.78	0.67	0.30	0.56	0.78	0.78	0.46	0.46	0.32	0.32
Total livestock GHG emissions (t CO_2e)	3,864	3,881	4,364	3,627	5,691	2,041	2,041	1,877	1,877	2,120	2,120
**Soil organic carbon**											
Initial SOC stocks (t C ha^{-1}, 1m depth)	235	240	240	240	161	240	240	240	240	240	240
Final SOC stocks (t C ha^{-1}, 1m depth)	238	241	243	249	163	241	241	242	242	248	248
SOC change (t C ha^{-1} yr^{-1})	0.14	0.06	0.12	0.45	0.11	0.06	0.06	0.10	0.10	0.37	0.37
SOC change (t CO_2e ha^{-1} yr^{-1})	0.53	0.21	0.44	1.65	0.40	0.21	0.21	0.37	0.37	1.36	1.36
Total SOC change (t CO_2e yr^{-1})	301	119	250	945	523	119	119	211	211	774	774
**Forestry system**											
Site C change (t C ha^{-1} yr^{-1})	-	-	-	8.3	-	-	8.3	8.3	8.3	8.3	8.3
Site C change (t CO_2e ha^{-1} yr^{-1})	-	-	-	30.5	-	-	30.5	30.5	30.5	30.5	30.5
Site C change x 50 ha (t CO_2e yr^{-1})	-	-	-	1,527	-	-	1,527	1,527	1,678	1,527	1,678
**Net GHG emissions**											
Net farm emissions (t CO_2e)	3,563	3,762	4,114	1,155	5,168	1,921	396	139	-13	-180	-331
Net emission intensity (kg CO_2e kg^{-1} LW produced)	12.4	12.9	12.4	7.8	15.1	6.6	1.4	0.4	-0.0	-0.6	-1.1
Net emission intensity (kg CO_2e kg^{-1} protein)	69	72	69	43	83	37	7.6	2.4	-0.2	-3.3	-6.0
**Economics**											

Earnings before interests and taxes (‘000 AU$)	487	500	621	667	721	459	414	613	609	537	530
Return on Capital (RoC, %)	4.04	4.15	5.13	5.50	4.09	3.81	3.30	4.91	4.77	4.25	4.09

Supplementary table 7. Biophysical, environmental, and economic outcomes associated with pathways to net zero emissions in the high rainfall beef production system in 2050. Hist: historical, Base: baseline farm with no adaptation, LHF: Low Hanging Fruit and TCN: Towards Carbon Neutral. Hist: scenarios simulated with historical climates. Base: impact of future climates. LHF: low-hanging fruit packages. TCN: towards carbon neutrality package. ID: income diversification. Asp: *A. taxiformis*. Asp+PT: *A. taxiformis* + Planting trees 50ha. CN1: carbon neutral package 1 (*A. taxiformis*+ planting trees 50ha+ transformational feed conversion efficiency. CN2: carbon neutral package 2 (*A. taxiformis*+ planting trees 110ha + transformational feed conversion efficiency. CN3: carbon neutral package 2 (*A. taxiformis* + planting trees 50ha + Lucerne). CN4: carbon neutral package 4 (*A. taxiformis* + planting trees 110ha + Lucerne)

**Variables**	**Scenarios**										
	**Hist**	**Base50**	**LHF**	**TCN**	**ID**	**Asp**	**Asp+PT**	**CN1**	**CN2**	**CN3**	**CN4**
**Livestock System**											
Stocking Rate (DSE ha^{-1} yr^{-1})	24.2	24.4	25.2	25.6	12.3	24.4	24.4	21.6	21.6	25.0	25.0
Farm Liveweight Production (t LW yr^{-1})	287	290	332	349	333.6	290	290	326	326	309	309
Protein Production (t protein yr^{-1})	52	52	60	63	60	52	52	59	59	56	56
Pasture Production (t DM ha^{-1} yr^{-1})	20.0	20.3	21.2	19.8	12.0	20.3	20.3	20.3	20.3	19.2	19.2
Supplementary Feeding (t DM ha^{-1} yr^{-1})	0.80	0.79	0.67	0.29	0.59	0.79	0.79	0.39	0.39	0.35	0.35
Total livestock GHG emissions (t CO_2e)	3,864	3,890	4,364	3,736	5,635	2,045	2,045	1,869	1,869	2,196	2,196
**Soil organic carbon**											
Initial SOC stocks (t C ha^{-1}, 1m depth)	235	241	243	249	163	241	241	242	242	248	248
Final SOC stocks (t C ha^{-1}, 1m depth)	238	241	244	254	164	241	241	243	243	251	251
SOC change (t C ha^{-1} yr^{-1})	0.14	-0.05	0.06	0.21	0.04	-0.05	-0.05	0.03	0.03	0.19	0.19
SOC change (t CO_2e ha^{-1} yr^{-1})	0.53	-0.18	0.20	0.77	0.16	-0.18	-0.18	0.10	0.10	0.68	0.68
Total SOC change (t CO_2e yr^{-1})	301	-102	115	438	204	-102	-102	58	58	387	387
**Forestry system**											
Site C change (t C ha^{-1} yr^{-1})	-	-	-	4.6	-	-	4.6	4.6	4.6	4.6	4.6
Site C change (t CO_2e ha^{-1} yr^{-1})	-	-	-	16.7	-	-	16.7	16.7	16.7	16.7	16.7
Site C change (t CO_2e yr^{-1})	-	-	-	836	-	-	836	836	1,837	836	1,837
**Net GHG emissions**											
Net farm emissions (t CO_2e)	3,563	3,992	4,250	2,462	5,431	2,147	1,312	976	-26	973	-28
Net emission intensity (kg CO_2e kg^{-1} LW produced)	12.4	13.8	12.8	9.5	16.3	7.4	4.5	3.0	-0.1	3.1	-0.1
Net emission intensity (kg CO_2e kg^{-1} protein)	69	76	71	53	91	41	25	16.5	-0.4	17.4	-0.5
**Economics**											

Earnings before interests and taxes ('000 AU$)	487	500	627	686	644	458	409	649	646	550	543
Return on Capital (RoC, %)	4.04	4.15	5.18	5.66	3.65	3.80	3.26	5.22	5.10	4.35	4.20

Supplementary table 8. Biophysical, environmental, and economic outcomes associated with pathways to net zero emissions in the low rainfall sheep production system in 2030. Hist: historical, Base: baseline farm with no adaptation except removal of cattle, LHF: Low Hanging Fruit and TCN: Towards Carbon Neutral. Hist: scenarios simulated with historical climates. Base: impact of future climates. LHF: low-hanging fruit packages. TCN: towards carbon neutrality package. ID: income diversification with vineyard. Asp: *A. taxiformis*. Asp+PT: *A. taxiformis* + Planting trees 200ha. CN1: carbon neutral package 1 (*A. taxiformis*+ planting trees 200ha+ transformational feed conversion efficiency. CN2: carbon neutral package 2 (*A. taxiformis*+ planting trees 220ha + transformational feed conversion efficiency. CN3: carbon neutral package 2 (*A. taxiformis* + planting trees 200ha + Lucerne). CN4: carbon neutral package 4 (*A. taxiformis* + planting trees 220ha + Lucerne).

**Variable**	**Scenario**										
	**Hist**	**Base30**	**LHF**	**TCN**	**ID**	**Asp**	**Asp+PT**	**CN1**	**CN2**	**CN3**	**CN4**
**Livestock System**											
Stocking Rate (DSE ha^{-1} yr^{-1})	9.0	8.0	9.1	10.0	8.0	8.0	8.0	7.3	7.3	8.4	8.4
Farm Liveweight Production (t LW yr^{-1})	370.0	293.6	369.8	475.5	293.6	293.6	293.6	319.5	319.5	322.5	322.1
Farm Wool Production (t CFW yr^{-1})	71.0	79.0	86.5	93.7	79.0	79.0	79.0	72.0	72.0	82.1	82.1
Farm Livestock Production (t LW + CFW yr^{-1})	441.0	372.6	456.3	569.2	372.6	372.6	372.6	391.5	391.5	404.1	404.1
Protein Production (t protein yr^{-1})	137.6	131.9	153.1	179.3	133.3	131.9	131.9	129.5	129.5	140.0	140.0
Pasture Production (t DM ha^{-1} yr^{-1})	7.2	7.7	7.8	8.2	7.7	7.7	7.7	7.7	7.7	8.3	8.3
Supplementary Feeding (t DM ha^{-1} yr^{-1})	0.3	0.1	0.1	0.1	0.1	0.11	0.11	0.1	0.1	0.0	0.0
Total livestock GHG emissions (t CO_2e)	7,037	6,375	7,666	6,510	6,375	2,662	2,662	2,455	2,455	2,797	2,797
**Soil organic carbon**											
Initial SOC stocks (t C ha^{-1}, 1m depth)	175.0	182.5	182.5	182.5	182.5	182.5	182.5	182.5	182.5	182.5	182.5
Final SOC stocks (t C ha^{-1}, 1m depth)	179.2	184.1	185.4	185.6	184.1	184.1	184.1	184.7	184.7	186.0	186.0
SOC change (t C ha^{-1} yr^{-1})	0.21	0.08	0.15	0.16	0.08	0.08	0.08	0.11	0.11	0.17	0.17
SOC change (t CO_2e ha^{-1} yr^{-1})	0.77	0.29	0.53	0.57	0.29	0.29	0.29	0.41	0.41	0.64	0.64
SOC change (t CO_2e yr^{-1})	2,425	910	1,686	1,815	910	910	910	1,298	1,298	2,020	2,020
**Forestry/horticulture system**											
Site C change (t C ha^{-1} yr^{-1})	-	-	-	1.5	0.6	-	1.5	1.5	1.5	1.5	1.5
Site C change (t CO_2e ha^{-1} yr^{-1})	-	-	-	5.4	2.1	-	5.4	5.4	5.4	5.4	5.4
Farm Grape Production (t fresh fruit yr^{-1})	-	-	-	-	300	-	-	-	-	-	-
Grapes GHG emissions (t CO_2e ha^{-1} yr^{-1})	-	-	-	-	1.1	-	-	-	-	-	-
Total site C change (t CO_2e yr^{-1})	-	-	-	1,073	29	-	1,073	1,073	1,180	1,073	1,180
**Net GHG emissions**											
Net farm emissions (t CO_2e)	4,612	5,466	5,980	3,623	5,436	1,752	680	83	-24	-296	-403

Variable	Scenario										
	Hist	Base30	LHF	TCN	ID	Asp	Asp+PT	CN1	CN2	CN3	CN4
Net emission intensity ($kg\ CO_2e\ kg^{-1}$ LW produced)	6.0	7.5	7.0	3.6	7.5	2.4	0.9	0.3	-0.03	-0.4	-0.5
Net emission intensity ($kg\ CO_2e\ kg^{-1}$ CFW produced)	33.5	41.5	39.1	20.2	41.5	13.3	5.2	0.1	-0.2	-2.1	-2.9
Net emission intensity ($kg\ CO_2e\ kg^{-1}$ fruit produced)	-	-	-	-	-0.01	-	-	-	-	-	-
Net emission intensity ($kg\ CO_2e\ kg^{-1}$ LW + CFW produced)	10.5	14.7	13.1	6.4	14.6	4.7	1.8	0.2	-0.1	-0.7	-1.0
Net emission intensity ($kg\ CO_2e\ kg^{-1}$ protein)	33.5	41.5	39.1	20.2	40.8	13.3	5.2	0.6	-0.2	-2.1	-2.9
**Economics**											
Earnings before interests and taxes ('000 AU$)	919	1,246	1,481	1,991	1,510	1,158	1,137	1,231	1,228	1,327	1,325
Return on Capital (RoC, %)	5.11	6.93	8.10	10.34	8.40	6.44	6.22	6.73	6.71	7.03	7.01

Supplementary table 9. Biophysical, environmental, and economic outcomes associated with pathways to net zero emissions in the high rainfall sheep production system in 2050. Hist: historical, Base: baseline farm with no adaptation except removal of cattle, LHF: Low Hanging Fruit and TCN: Towards Carbon Neutral. Hist: scenarios simulated with historical climates. Base: impact of future climates. LHF: low-hanging fruit packages. TCN: towards carbon neutrality package. ID: income diversification with vineyard. Asp: *A. taxiformis*. Asp+PT: *A. taxiformis* + Planting trees 200ha. CN1: carbon neutral package 1 (*A. taxiformis*+ planting trees 200ha+ transformational feed conversion efficiency. CN2: carbon neutral package 2 (*A. taxiformis*+ planting trees 220ha + transformational feed conversion efficiency. CN3: carbon neutral package 2 (*A. taxiformis*+ planting trees 200ha + Lucerne). CN4: carbon neutral package 4 (*A. taxiformis*+ planting trees 220ha + Lucerne).

**Variable**	**Scenario**										
	**Hist**	**Base50**	**LHF**	**TCN**	**ID**	**Asp**	**Asp+PT**	**CN1**	**CN2**	**CN3**	**CN4**
**Livestock System**											
Stocking Rate (DSE ha^{-1} yr^{-1})	9.0	8.0	9.1	10.4	8.0	8.0	8.0	7.0	7.0	8.4	8.4
Farm Liveweight Production (t LW yr^{-1})	370.0	297.2	379.5	493.0	297.2	297.2	297.2	334.4	334.4	328.6	328.6
Farm Wool Production (t CFW yr^{-1})	71.0	77.6	86.0	96.0	77.6	77.6	77.6	68.8	68.8	81.8	81.8
Farm Livestock Production (t LW + CFW yr^{-1})	441.0	374.8	465.6	589.0	374.8	374.8	374.8	403.2	403.2	410.5	410.5
Protein Production (t protein yr^{-1})	137.6	131.1	154.4	184.7	132.5	131.1	131.1	129.0	129.0	141.0	141.0
Pasture Production (t DM ha^{-1} yr^{-1})	7.2	7.9	8.0	8.6	7.9	7.9	7.9	7.8	7.8	8.6	8.6
Supplementary Feeding (t DM ha^{-1} yr^{-1})	0.3	0.1	0.1	0.1	0.1	0.12	0.12	0.1	0.1	0.0	0.0
Total livestock GHG emissions (t CO_2e)	7,037	6,332	7,676	6,647	6,332	2,622	2,622	2,366	2,366	2,862	2,862
**Soil organic carbon**											
Initial SOC stocks (t C ha^{-1}, 1m depth)	175.0	184.1	185.4	185.6	184.1	184.1	184.1	184.7	184.7	185.8	185.8
Final SOC stocks (t C ha^{-1}, 1m depth)	179.2	186.0	188.0	188.6	186.0	186.0	186.0	186.4	186.4	189.4	189.4
SOC change (t C ha^{-1} yr^{-1})	0.21	0.10	0.13	0.15	0.10	0.10	0.10	0.09	0.09	0.17	0.17
SOC change (t CO_2e ha^{-1} yr^{-1})	0.77	0.36	0.48	0.54	0.36	0.36	0.36	0.31	0.31	0.64	0.64
SOC change (t CO_2e yr^{-1})	2,425	1,142	1,531	1,719	1,142	1,142	1,142	993	993	2,032	2,032
**Forestry/horticulture system**											
Site C change (t C ha^{-1} yr^{-1})	-	-	-	1.7	0.6	-	-	1.7	1.7	1.7	1.7
Site C change (t CO_2e ha^{-1} yr^{-1})	-	-	-	6.2	2.1			6.2	6.2	6.2	6.2
Farm Grape Production (t fresh fruit yr^{-1})					300						
Grapes GHG emissions (t CO_2e ha^{-1} yr^{-1})	-	-	-	-	1.1	-	-	-	-	-	-
Total site C change (t CO_2e yr^{-1})	-	-	-	1,247	29	-	1,247	1,247	1,372	1,247	1,372
**Net GHG emissions**											
Net farm emissions (t CO_2e)	4,612	5,190	6,144	3,680	5,160	1,480	233	125	0	-418	-543

Variable	Scenario										
	Hist	Base50	LHF	TCN	ID	Asp	Asp+PT	CN1	CN2	CN3	CN4
Net emission intensity (kg CO_2e kg^{-1} LW produced)	6.0	7.1	7.2	3.6	7.1	2.0	0.3	0.0	0.0	-0.5	-0.7
Net emission intensity (kg CO_2e kg^{-1} CFW produced)	33.5	39.6	39.8	19.9	39.6	11.3	1.8	0.0	0.0	-3.0	-3.9
Net emission intensity (kg CO_2e kg^{-1} fruit produced)	-	-	-	-	-0.01	-	-	-	-	-	-
Net emission intensity (kg CO_2e kg^{-1} LW + CFW produced)	10.5	13.8	13.2	6.2	13.8	3.9	0.6	0.3	0.0	-1.0	-1.3
Net emission intensity (kg CO_2e kg^{-1} protein)	33.5	39.6	39.8	19.9	39.0	11.3	1.8	0.0	0.0	-3.0	-3.9
**Economics**											
Earnings before interests and taxes ('000 AU$)	919	1,249	1,514	2,095	1,513	1,161	1,140	1,268	1,266	1,355	1,353
Return on Capital (RoC, %)	5.11	6.95	8.27	10.88	8.42	6.46	6.34	6.92	6.92	7.18	7.17

Supplementary table 10. Parameters of, and distributions for, capital costs and annual variable costs. DM = dry matter; DSE = dry sheep equivalent hd = head; ID = income diversification; TCN = Towards Carbon Neutral.

Variables	Beef farm	Sheep farm
**Capital costs (Year 1)**		
Land	13,237,500	13,250,000
Livestock	3,941,860	3,600,000
Machinery	1,113,425	1,100,000
Water	32,350	32,000
**Annual Farm Variable costs** (exc. supp feed)	635,230	561,525
Annual Supplementary feed	100,000	48,000
Annual Farm Cash Overhead costs	516,480	600,000
**TCN, ID and transformational options**		
Land for trees purchase cost ($ ha^{-1})	10,000	-
Land for farm expansion ($ ha^{-1})	10,000	-
Additional overhead costs with land expansion ($ $annum^{-1}$)	50,000	-
Trees Establishment ($ ha^{-1})	1,500	1,500
Trees maintenance p.a. ($ ha^{-1} $annum^{-1}$)	30	30
Trees depreciation ($ ha^{-1} $annum^{-1}$)	75	75
Methane vaccine ($ hd^{-1} $annum^{-1}$)	Uniform<br>Mean= 10<br>Max= 15<br>Min= 5	Uniform<br>Mean= 6.5<br>Max= 10<br>Min= 3
Extra cattle variable cost ($ DSE^{-1} $annum^{-1}$)	11.70	-
Extra sheep variable cost ($ DSE^{-1} $annum^{-1}$)	-	30
Extra livestock depreciation cost ($ DSE^{-1} $annum^{-1}$)	11.25	11.25
Lucerne establishment cost ($ ha^{-1})	400	400
Lucerne depreciation ($ ha^{-1} $annum^{-1}$)	40	40
Extra annual fertiliser maintenance ($ ha^{-1} $annum^{-1}$)	50	50
Vineyard Establishment ($ ha^{-1})	-	300,000
Net profit of vineyard ($ ha^{-1} $annum^{-1}$)	-	10,000
Vineyard depreciation ($ ha^{-1} $annum^{-1}$)	-	1,200
Biochar ($ kg DM^{-1})	1.4	2
Feeding *Asparagopsis taxiformis* ($ kg DM^{-1})	2	2

Supplementary table 11. Co-benefits and trade-offs associated with practices and technologies used in carbon neutral packages raised by the Regional Reference Group. CN: carbon neutral, GHG: greenhouse gas emissions; TFCE = transformational feed conversion efficiency.

CN package	Co-benefits	Trade-offs
Asparagopsis spp.	-Large reductions in methane emissions.<br>-Carbon sequestration (ocean)[21]<br>-Better water quality.<br>-Remove nutrients in excess from the ocean and reduce acidification.	-High cost/investment.<br>-Scalability.<br>-Formulation/delivery.<br>-Long-term animal health and safety.<br>-Invasive species introduction.
Planting trees	-Carbon “offsetting” and “insetting”.<br>-Potential agroforestry systems.<br>-Increase natural capital and farm diversification.<br>-Shelterbelt for animals.	-High cost/investment.<br>-More infrastructure (fencing).<br>-Irreversibility to productive land.
TFCE	-High animal performance.<br>-Large reductions in GHG emissions.<br>-Reduction of production costs.	-Results in the long-term.<br>-Holistic farm management (epigenetics).<br>-Large capital investment in animals with new genetics.
Lucerne	-Farmer’s acceptance to increase farm area covered by legumes.<br>-Potential association between legume management and delivery of *Asparagopsis spp*.<br>-Nitrogen fixation.	-Expensive if it fails.<br>-Adaptation period of ruminants to legumes (ruminant health risk if excessive legume consumed)

Supplementary table 12. Thematic adaptation/mitigation interventions co-designed with a Regional Reference Group (RRG). Interventions were categorized into themes (low-hanging fruit, towards carbon neutral, income diversification and transformational adaptations). The extent to which each factor was varied from the baseline level was derived from the RRG and values from the literature. CFA = cast for age; CN = carbon neutral; DM = dry matter; FCE = feed conversion efficiency; FullCAM = Australian Government full carbon accounting model; LW = liveweight; SOC = soil organic carbon; SB-GAF = Sheep-Beef Greenhouse Accounting Framework; TCN = towards carbon neutral; TFCE = transformational feed conversion efficiency; RD = root depth; RRG = regional reference group.

	**Adaptation**	**Assumptions**	**References**
Low-hanging fruit adaptation	Increasing soil fertility	Increase soil fertility 3% in all paddocks (3% higher fertility scalar in GrassGro). Fertility scalar is 0-1 value that represents the degree to which a pasture growth will be restricted by soil fertility at times when soil water availability does not limit pasture growth.	22
	Introduction of Talish clover	Paddocks grazed by wool and prime lamb flocks in the sheep farm including Talish clover (*Trifolium tumens*) with 600 mm rooting depth, adapted from white clover in GrassGro. No changes to baseline fertiliser were modelled in SB-GAF, as pastures contained perennial ryegrass, which requires synthetic N input.	23
	Increasing root depth by 10%	Increased rooting depth by 10% in all the modelled species within the paddock (upper limit 1200 mm)	RRG
	Increasing stocking rate by 10%	Increasing notional stocking rate by 10% from the baseline) in the beef and sheep farm. The notional stocking rate and weather stocking rate (in animals ha^{-1}) immediately after replacement animals are acquired or mature wethers.	RRG
	Shifting forward calving/lambing date in line with warmer climates increasing growth in winter	We altered lambing/calving dates and selling dates/stocking rate/liveweight to better match seasonal pasture supply.<br>Management rules for the beef farm:<br>Mate 8 Oct, Calving 18 Jul, wean 7 Feb (29 wks). The calves were 68 kg LW $head^{-1}$ (earlier birth and 18 kg LW $head^{-1}$ higher)	RRG

		Sell excess heifers 8 Oct (27 months) or at 600 kg target LW. Feed steers in a paddock from 1 Feb to reach 535 kg LW $head^{-1}$ on 31 Aug. Feed heifers in a paddock from 1 Feb to reach 520 kg LW $head^{-1}$ on 15 Sep.<br><br>Management rules for the sheep farm:<br>Self-replacing Merino flock, replace 18 Aug<br>CFA ewes sold 17 Aug (4-5 yrs) into lamb flock. Mate 8 Apr, lamb 4 Sep, wean 17 Jan (19 wks) and sell excess 10 Feb as preliminary modelling results suggest feed available until then. Shearing 6 Jul.<br>Purchased 18 Aug (from wool flock) at 24 mths of age. Mate 14 days sooner so 28 Mar, lamb 24 Aug, wean 20 Dec (17 wks) as feed remained until then. Shearing 6 Jul.	
	Increasing FCE	Increasing feed conversion efficiency 10% in 2030 and 15% in 2050 period. Alter FCE in GrassGro increasing factors by 10%, the parameters c-k-1, c-k-2, c-k-13 and c-k-14 were 0.55, 0.022, 0.0385 and 0.363 or 15%, the parameters c-k-1, c-k-2, c-k-13 and c-k-14 were 0.575, 0.0253, 0.0403 and 0.378.<br>C-k-1 and c-k-2 = parameters controlling efficiency of maintenance<br>C-k-13 and c-k-14 = parameters controlling efficiency of gain	24, 25
Towards carbon neutral adaptations	Pasture renovation with lucerne	Renovation of existing perennial ryegrass swards with lucerne (semi-winter active, 1200 mm RD) as deep-rooted species in all paddocks for the beef and sheep farm. Pasture renovation occurred via seed broadcasting. No change to baseline N fertiliser management in SB-GAF were made to the beef cattle farm, given swards were mixed with perennial ryegrass and have high production potential (high N demand). No changes to baseline N fertiliser in SB-GAF were made for the sheep farm.	RRG

	Enteric CH_4 inhibitor vaccine	Reducing enteric CH_4 fermentation by 30% to reflect an intervention reducing CH_4 emissions by a modest amount.	26
	Planting trees	For the beef farm:<br>Extra 50 ha for environmental plantings in FullCAM (Tasmanian Blue Gums, *Eucalyptus globolus*).<br><br>For the sheep farm:<br>Additional 200-220 ha of trees planted with environmentally bespoke species simulated using FullCAM via through thickening of existing non-grazed vegetation areas for the TCN and CN packages (trees, shrubs and understory species endemic to the region).	27,28
Income diversification adaptations	Buying a farm in a different region for enterprise climate diversification	Buying an extra farm in a different agroclimatic region (by translocating cow calf systems to Gladstone, NE Tasmania and dedicating the current farm to backgrounding and finishing of weaners). More details about soil and pasture type, pasture and herd management in supplementary table 5.	See Supplementary table 5
	Diversifying land use with grapes	Diversifying land use with grapes (by thickening 30 ha land for sheep farm with a vineyard for Pinot Noir). More details about vineyard management, production and assumptions can be found in subsection Diversifying land use with grapes on a sheep farm.	See subsection Diversifying land use with grapes on a sheep farm.
	Installing wind turbines	This adaptation implicates a 35 year project leasing a small part of the land to host 12 wind turbines on the beef farm. Given that each turbine generates 7,500 $ yr^{-1} over 35 years, and our simulation was for a 40 year period, we assumed an income of 7,500 $ yr^{-1} from 2022 to 2041 and 5,625 $ yr^{-1} from 2042 to 2061 for each turbine as in supplementary equation (8):<br><br>$\text{Annual income per wind turbine (period 2022} - 2041, \$\ \text{yr}^{-1}) = 7500\ \$\ \text{yr}^{-1}$	29

		$\text{Annual income per wind turbine (period 2042} - \text{2061, \$ yr}^{-1}) = \frac{7500\ \$\ \text{yr}^{-1} * 15\ \text{yr}}{20\ \text{yr}} = 5625\ \$\ \text{yr}^{-1}$ **(8)**<br>15 is the remaining number of years of the project in the second simulated period and 20 is the total number of years for such period of time.	
Transformational adaptations	Biochar as a feed supplement	For this adaptation we assumed an increase in liveweight production by 5% for the beef farm and reduced enteric CH_4 fermentation in SB-GAF by 10% for both case studies. Effects of biochar on SOC changes by C enrichment in manure are described in 'Accounting for carbon changes in soil by enrichment of manure with biochar'.	See subsection 'Accounting for carbon changes in soil by enrichment of manure with biochar'
	Red seaweed (*Asparagopsis taxiformis*) as a feed supplement	Reduce enteric CH_4 fermentation in SB-GAF by 80% to reflect transformational intervention to reduce GHG emissions of all weaned animals on farm. We assumed 0.5% of dietary intake would comprise *Asparagopsis taxiformis* as in supplementary equation (9):<br><br>$A.taxiformis\ (\text{kg DM}) = [\text{Total pasture intake (kg DM)} + \text{Supplement intake (kg DM)}] * 0.005$ **(9)**	30-32
	Transformational increases in FCE (20-30%)	Increasing feed conversion efficiency by 20% in 2030 and by 30% in 2050. In GrassGro this was implemented by increasing parameters by 20% (c-k-1, c-k-2, c-k-13 and c-k-14 were 0.60, 0.024, 0.042 and 0.396) or 30% (c-k-1, c-k-2, c-k-13 and c-k-14 were 0.65, 0.026, 0.0455 and 0.429), where the former group relate to efficiency of maintenance and the latter control efficiency of gain.	24,25

Supplementary table 13. Monthly change factors showing fractional change in temperate and rainfall for 2030 and 2050 relative to historical monthly average values. Calculated using raw data from Harris et al. [33] for Representative Concentration Pathways 8.5 (RCP8.5)

		Sheep farm		Beef farm	
		Rainfall	Temperature	Rainfall	Temperature
2030	Jan	1.06	1.04	0.99	1.05
	Feb	1.06	1.04	0.99	1.05
	Mar	0.97	1.05	0.94	1.05
	Apr	0.97	1.05	0.94	1.05
	May	0.97	1.05	0.94	1.05
	Jun	0.95	1.08	0.93	1.06
	Jul	0.95	1.08	0.93	1.06
	Aug	0.95	1.08	0.93	1.06
	Sep	0.92	1.07	0.89	1.06
	Oct	0.92	1.07	0.89	1.06
	Nov	0.92	1.07	0.89	1.06
	Dec	1.06	1.04	0.99	1.05
	**Avg**	0.97	1.06	0.94	1.06
2050	Jan	1.04	1.08	0.95	1.09
	Feb	1.04	1.08	0.95	1.09
	Mar	0.94	1.09	0.89	1.09
	Apr	0.94	1.09	0.89	1.09
	May	0.94	1.09	0.89	1.09
	Jun	0.94	1.14	0.89	1.11
	Jul	0.94	1.14	0.89	1.11
	Aug	0.94	1.14	0.89	1.11
	Sep	0.90	1.11	0.86	1.10
	Oct	0.90	1.11	0.86	1.10
	Nov	0.90	1.11	0.86	1.10
	Dec	1.04	1.08	0.95	1.09
	**Avg**	0.96	1.11	0.90	1.10

Supplementary table 14. Baseline characteristics of the beef case study farm. Summary of parameters and biophysical variables such as herd structure and dynamics, livestock, pasture and soil management, including key input factors and assumptions for each thematic adaptation which comprised multiple stacked incremental adaptations suggested by the Regional Reference Group. CFA = cast for age; CP = crude protein; CS = condition score; DM = dry matter; FS = fertility scalar; LW = liveweight; ME = metabolizable energy; MJ = megajoules; RD = root depth.

Herd	Variable	Historical/future climate
**Main herd** **(Cow-calf and home-bred young stock herd)**	Area grazed	· 402 ha
	Livestock numbers	· Stocking rate of 1.1 cows ha^{-1}
	Livestock management	· Breed: Angus<br>· Average liveweight at the start of the analysis:<br>-Cows 580 kg LW $head^{-1}$<br>-Weaners 240 kg LW $head^{-1}$<br>-Yearlings 425 kg LW $head^{-1}$ steers and 400 kg LW $head^{-1}$ heifers<br>-2-3 years old 650 kg LW $head^{-1}$ steers and 625 kg LW $head^{-1}$ heifers<br>-Calves 50 kg LW $head^{-1}$<br>· Self-replacing herd, replace 11 Feb<br>· Culled cows sold on 10 Feb (6-7 yrs)<br>· Sell excess heifers 30 Sep (26 months) or at 600 kg target LW<br>· Sell steers 15 Sep (25 months) or at 650 kg target LW<br>· Mate 23 Oct, Calving 2 Aug, wean 7 Feb (27 weeks)<br>· Age of first joining 1-2 years<br>· 1 bull 25 $cows^{-1}$ (kept for 4 years)<br>· Maint. feed females, when thinnest CS2.5<br>· Maint. feed weaners in paddock when thinnest CS3<br>· Maint. feed 100% hay (DM 85%, ME 11.5 MJ kg DM^{-1}, CP 20%)<br>· Production feeding rule- feedlot cows every year in feedlot and feed 5.5 kg $head^{-1}$ to oldest cows from 1 Jul to 31 Jul

	· Feed steers in a paddock from 1 Feb to reach 515 kg LW head^{-1} on 31 Aug<br>· Feed heifers in a paddock from 1 Feb to reach 505 kg LW head^{-1} on 15 Sep
Livestock genetics	· Default within GrassGro for c-k-1, c-k-2, c-k-13 and c-k-14 are 0.5, 0.02, 0.035 and 0.33<br>· Conception rate 95%<br>· Analysis of historical mortality rate from GrassGro 0.5%
Pasture types	· Paddock 1 (8 ha), Irrigated Perennial Ryegrass (*Lolium perenne* L.; 720 mm rooting depth (RD)), Cocksfoot (*Dactylis glomerata* L.; 850 mm RD) and White Clover (*Trifolium repens* L.; 500 mm RD)<br>· Paddock 2 (20 ha), Irrigated Lucerne-semi winter active (*Medicago sativa;* 1200 mm RD), Perennial Ryegrass (720 mm RD)<br>· Paddock 3 (187 ha), Rainfed Perennial Ryegrass (720 mm RD), Cocksfoot (850 mm RD) and Subterranean Clover – Seaton Park (*Trifolium subterraneum* L.; 600 mm RD)<br>· Paddock 4 (187 ha), Rainfed Perennial Ryegrass (750 mm RD) and White Clover (500 mm RD)
Pasture management (note rooting depth in pasture type)	· Irrigate paddock 1 and 2 between 21 Nov and 31 Mar, applying 20 mm and fill to 0.95<br>· Cut paddocks 3 and 4 (whenever DM yield exceeds 5000 kg ha^{-1} between 2 Sep-14 Dec). Proportion gathered 90%. Cutting height 125 mm. Don't cut when DM is below 800 kg ha^{-1}<br>· Uniform application of 51 kg N ha^{-1} annum^{-1}
Grazing management	· Cows- From 1 Jul to 30 Jun graze paddocks 3 and 4, withhold 21 days, check every 4 days and move when weight gain margin is > 0.01 kg day^{-1}<br>· Heifer Weaners- From 1 Jul to 30 Jun graze paddocks 3 and 4, withhold 21 days, check every 4 days and move when weight gain margin is > 0.01 kg day^{-1}<br>· Heifer Yearlings- From 1 Jul to 30 Jun graze paddocks 3 and 4, withhold 21 days, check every 4 days and move when weight gain margin is > 0.01 kg day^{-1}<br>· Steers Weaners- From 1 Jul to 30 Jun graze paddocks 1, 2, 3 and 4, withhold 21 days, check every 4 days and move when weight gain margin is > 0.01 kg day^{-1}<br>· Steers Yearlings- From 1 Jul to 30 Jun graze paddocks 2, 3 and 4, withhold 21 days, check every 4 days and move when weight gain margin is > 0.01 kg day^{-1}<br>· Steers 2-3 years old - From 1 Jul to 30 Jun graze paddocks 3 and 4, withhold 21 days, check every 4 days and move when weight gain margin is > 0.01 kg day^{-1}
Soils	· All paddocks soil texture defined from Atlas in GrassGro, corresponding to a Northcote Uc2.3 classification[20]<br>· Paddocks 1 and 2 FS 0.87<br>· Paddocks 3 and 4 FS 0.85

	Tree plantings	· No environmental plantings beyond currently on farm
**Purchased weaner herd**	Area grazed	· 127 ha
	Livestock numbers	· Stocking rate of 1.8 steers ha^{-1}
	Livestock management	· Breed: Angus<br>· Average liveweight at the start of the analysis:<br>-Weaners 225 kg LW $head^{-1}$<br>-Yearlings 425 kg LW $head^{-1}$<br>-2-3 years old 650 kg LW $head^{-1}$<br>-3-4 years old 700 kg LW $head^{-1}$<br>· Purchased 1 Feb at 6 mths of age and sold on 15 Sep (25 mths) or at 633 kg LW $head^{-1}$<br>· Maint. feed mature males and weaners in paddock when thinnest CS2.5.<br>· Maint. feed 100% hay (DM 85%, ME 11.5 MJ kg DM^{-1}, CP 20%)<br>· Feed steers in a paddock from 1 Feb to reach 500 kg LW $head^{-1}$ on 1 Sep
	Livestock genetics	· Default within GrassGro for c-k-1, c-k-2, c-k-13 and c-k-14 are 0.5, 0.02, 0.035 and 0.33
	Pasture types	· Paddock 1 (32 ha), Rainfed Perennial Ryegrass (720 mm RD) and White Clover (500 mm RD)<br>· Paddock 2 (32 ha), Rainfed Perennial Ryegrass (720 mm RD) and White Clover (500 mm RD)<br>· Paddock 3 (31.5 ha), Rainfed Perennial Ryegrass (720 mm RD) and White Clover (500 mm RD)<br>· Paddock 3 (31.5 ha), Rainfed Perennial Ryegrass (720 mm RD) and White Clover (500 mm RD)
	Pasture management	· Reset pasture species as necessary 1 Feb<br>· Cut paddocks 1 (Years: 1 and 4), 2 (Years: 1 and 2), 3 (Years: 2 and 3) and 4 (Years: 3 and 5) (whenever DM yield exceeds 5000 kg ha^{-1} between 2 Sep-14 Dec). Proportion gathered 90%. Cutting height 125 mm. Do not cut when DM is below 800 kg ha^{-1}
	Grazing management	· Weaners, Yearlings and 2-3 years old- From 1 Jan to 31 Dec graze paddocks 1, 2, 3 and 4, withhold 14 days, check every 7 days and move when weight gain margin is > 0.01 kg day-1
	Soils	· All paddocks soil texture defined from Atlas in GrassGro, corresponding to a Northcote Uc2.3 classification[20]<br>· All paddocks FS 0.85
	Tree plantings	· No environmental plantings above what currently on farm
**Purchased yearlings with agisted heifers**	Area grazed	· 40 ha
	Livestock numbers	· Stocking rate of 3.9 steers ha^{-1}
	Livestock management	· Breed: Angus

	· Purchased 1 Feb at 16 mths of age (375 kg LW head^{-1}) and sold on 15 Sep (28 mths) or at 545 kg LW head^{-1}<br>· Maint. feed steers in paddock when thinnest CS2.<br>· Maint. feed 100% hay (DM 85%, ME 11.5 MJ kg DM^{-1}, CP 20%)<br>· Feed steers in a paddock from 1 Feb to reach 350 kg LW head^{-1} on 1 Sep
Livestock genetics	· Default within GrassGro for c-k-1, c-k-2, c-k-13 and c-k-14 are 0.5, 0.02, 0.035 and 0.33
Pasture types	· Paddock 1 (20 ha), Rainfed Perennial Ryegrass (720 mm RD) and White Clover (500 mm RD)<br>· Paddock 2 (20 ha), Rainfed Perennial Ryegrass (720 mm RD) and White Clover (500 mm RD)
Pasture management	· No hay cutting<br>· Uniform application of 51 kg N ha^{-1} annum^{-1}
Grazing management	· Steers (Yearling and 2-3 years old)- From 1 Jan to 31 Dec graze paddocks 1 and 2, withhold 14 days, check every 7 days and move when weight gain margin is > 0.01 kg day^{-1}
Soils	· All paddocks soil texture defined from Atlas in GrassGro, corresponding to a Northcote Uc2.3 classification[20]<br>· Paddock 1 and 2 FS 0.82
Tree plantings	· No environmental plantings above what currently on farm

Supplementary table 15. Baseline characteristics of the sheep case study farm. Summary of parameters and biophysical variables such as flock structure and dynamics, livestock, pasture and soil management, including key input factors and assumptions for each thematic adaptation which comprised multiple stacked incremental adaptations suggested by the Regional Reference Group. CFA = cast for age; CP = crude protein; CS = condition score; DM = dry matter; FS = fertility scalar; LW = liveweight; ME = metabolizable energy; MJ = megajoules; RD = root depth.

Flock/herd	Variable	Historical/future climate
**Wool flock**	Area grazed	· 2,545 ha
	Livestock numbers	· Stocking rate of 2.8 ewes ha^{-1} and 2.7 wethers ha^{-1}
	Livestock management	· Self-replacing Merino flock, replace 1 Sep<br>· CFA ewes sold 31 Aug (4-5 yrs) into lamb flock<br>· CFA wethers 14 Oct (5-6 yrs)<br>· Mate 22 Apr, lamb 18 Sep, wean 31 Jan (19 wks) and sell excess 1 Feb (19 wks)<br>· Shearing 20 Jul<br>· Maint. feed ewes, wethers and weaners in paddock when thinnest CS2.5<br>· Maint. feed weaners in paddock when thinnest CS3<br>· Maint. feed 78% wheat, 22% hay (ME 12.3 MJ kg DM^{-1}, CP 12%)<br>· Production feeding rule- feedlot ewes every year in feedlot and feed 0.52 kg $head^{-1}$ from 15 Jan to 15 Apr, same quality as maint. feed<br>· No other production rule for weaner lambs or wethers
	Livestock genetics	· Default GrassGro parameters for c-k-1, c-k-2, c-k-13 and c-k-14 are 0.5, 0.02, 0.035 and 0.33<br>· Conception rate 88% singles, 0% twins and 0% triplets<br>· Analysis of historical mortality rate from GrassGro 16.2%
	Pasture types	· Paddock 1 (800 ha), rainfed Phalaris (*Phalaris aquatica* L.; 750 mm rooting depth (RD)) and subterranean clover (500 mm RD)<br>· Paddock 2 (1,553 ha), rainfed Wallaby grass (*Austrodanthonia spp.*; 790 mm rooting depth) and Weeping grass (*Microlaena stipoides*; 450 mm RD*)*<br>· Paddock 3 (64 ha), rainfed Phalaris seed crop (750 mm RD)

	· Paddock 4 (30 ha), rainfed Phalaris (750 mm RD) and subterranean clover (440 mm RD, lower rooting depth of subterranean clover to other paddocks due to soil conditions)<br>· Paddock 5 (67 ha), irrigated lucerne (900 mm RD)<br>· Paddock 6 (31 ha), irrigated annual ryegrass (*Lolium multiflorum*) as surrogate for dual purchase wheat (*Triticum aestivum;* 520mm RD)
Pasture management (note rooting depth in pasture type)	· Irrigate paddock 5 and 6 between 1 Sep and 31 Mar, applying 18 mm and fill to 0.95<br>· Reset pasture species as necessary on 5 Apr<br>· Cut paddock 5 (irrigated lucerne) 10 Nov<br>· Unform application of 35 kg N ha^{-1} $annum^{-1}$ to all improved pastures (nil fertiliser to native pastures)
Grazing management	· Ewes- 15 Jan to 30 Jun graze paddocks 1 and 3, withhold 14 days, check every 7 days and move when weight gain margin is > 0.02 kg day^{-1}<br>· Ewes- 1 Jul to 14 Jan graze paddock 1, withhold 14 days, check every 4 days and move when weight gain margin is > 0.02 kg day^{-1}<br>· Wethers- 15 Jan to 14 Mar graze paddocks 1 and 3, withhold 14 days, check every 4 days and move when weight gain margin is > 0.02 kg day^{-1}<br>· Wethers- 15 Mar to 30 Jun graze paddocks 1,2 and 3, withhold 14 days, check every 4 days and move when weight gain margin is > 0.02 kg day^{-1}<br>· Wethers- 1 Jul to 15 Sep graze paddocks 1 and 2, withhold 14 days, check every 4 days and move when weight gain margin is > 0.02 kg day^{-1}<br>· Wethers- 16 Sep to 14 Jan graze paddock 1, withhold 14 days, check every 4 days and move when weight gain margin is > 0.02 kg day^{-1}<br>· Ewe and wether weaners- 1 Jan to 14 Jan graze paddocks 1, 4 and 5, withhold 14 days, check every 4 days and move when weight gain margin is > 0.02 kg day^{-1}<br>· Ewe and wether weaners- 15 Jan to 30 Apr graze paddocks 1,3, 4 and 5, withhold 14 days, check every 4 days and move when weight gain margin is > 0.02 kg/ day^{-1}<br>· Ewe and wether weaners- 1 May to 31 May graze paddocks 1, 3, 4, 5 and 6, withhold 14 days, check every 4 days and move when weight gain margin is > 0.02 kg day^{-1}<br>· Ewe and wether weaners- 1 Jun to 30 Jun graze paddocks 1, 3, 4 and 6, withhold 14 days, check every 4 days and move when weight gain margin is > 0.02 kg day^{-1}<br>· Ewe and wether weaners- 1 Jul to 31 Aug graze paddocks 1, 4 and 6, withhold 14 days, check every 4 days and move when weight gain margin is > 0.02 kg day^{-1}<br>· Ewe and wether weaners- 1 Sep to 31 Dec graze paddocks 1 and 4, withhold 14 days, check every 4 days and move when weight gain margin is > 0.02 kg day^{-1}

	Soils	· All paddocks soil texture defined from Atlas in GrassGro, corresponding to a Northcote Dy5.61 classification[20]<br>· Paddocks 1, 3-6 FS 0.84<br>· Paddock 2 FS 0.80 (natives not fertilised, species altered, area changed etc)
	Tree plantings	· No environmental plantings beyond currently on farm
**Prime lamb flock**	Area grazed	· 360 ha
	Livestock numbers	· Stocking rate of 9.6 ewes ha^{-1}
	Livestock management	· Purchased 1 Sep (from wool flock) at 24 mths of age<br>· Mate 11 Apr, lamb 7 Sep, wean 15 Dec (14 wks), sell lambs 15 Dec at 27 kg<br>· Shearing 20 Jul<br>· CFA 16 Dec (3-4 yrs)<br>· Maint. feed ewes in paddock when thinnest CS2.5- rerun historical, 2030 and 2050 with CS 2.5<br>· Maint. feed weaners in paddock when thinnest CS3<br>· Maint. feed 78% wheat, 22% hay (ME 12.3 MJ kg DM^{-1}, CP 12%)<br>· Production feeding rule- feedlot ewes every year in feedlot and feed 0.52 kg head^{-1} from 15 Jan to 15 Apr, same quality as maint. feed<br>· No production feeding rule for lambs
	Livestock genetics	· Default within GrassGro for c-k-1, c-k-2, c-k-13 and c-k-14 are 0.5, 0.02, 0.035 and 0.33<br>· Conception rate 91% singles, 2% twins and 0% triplets<br>· Analysis of historical mortality rate from GrassGro 8.5%
	Pasture types	· Paddock 1 (120 ha), rainfed Phalaris (750 mm RD) and subterranean clover (500 mm RD)<br>· Paddock 2 and 3 a repeat of paddock 1
	Pasture management	· Reset pasture species as necessary 5 Apr (mimic subterranean clover germination if required)<br>· Cut one paddock each year 16 Dec, rotating between the three paddocks so always have 2 for grazing<br>· Unform application of 35 kg N ha^{-1} annum^{-1}
	Grazing management	· All sheep- 1 Jan to 31 Dec graze paddocks 1, 2 and 3, withhold 14 days, check every 4 days and move when weight gain margin is > 0.025 kg day^{-1}
	Soils	· All paddocks soil texture defined from Atlas in GrassGro, corresponding to a Northcote Dy5.61 classification[20]<br>· Paddocks 1, 2 &3 FS 0.85
	Tree plantings	· No environmental plantings above what currently on farm
**Cattle herd**	Area grazed	· 265 ha

Livestock numbers	· Stocking rate of 1.5 cows ha^{-1} (~ 337 cows, 55 replacement heifers age group^{-1}, 145 steers and 90 non-replacement heifers)
Livestock management	· Self-replacing Hereford herd 1 Apr<br>· CFA cows 31 Mar (7-8 yrs)<br>· Mate 20 Nov, wean 31 Mar, sell excess heifers 1 Apr at 31 weeks or 220 kg, sell steers 28 Feb at 18 months or 460 kg<br>· Maint. feed cows in paddock when thinnest CS3<br>· Maint. feed weaners in paddock when thinnest CS2.5<br>· Maint. feed 100% hay (ME 11.0 MJ kg DM^{-1}, CP 14%)<br>· Production feeding rule- feed steers in paddock from 1 Apr to reach 460 kg 28 Feb<br>· Production feeding rule- feed heifers in paddock from 1 Sep to reach 250 kg 31 Mar<br>· Production feed same quality hay as per maint. Feed
Livestock genetics	· Default within GrassGro<br>· Conception rate of 92% at CS3
Pasture types	· Paddock 1 (132.5 ha), rainfed Phalaris (750 mm RD) and subterranean clover (500 mm RD)<br>· Paddock 2 same as paddock 1
Pasture management	· Reset pasture species as necessary 5 Apr (mimic subterranean clover germination if required)<br>· No hay cutting<br>· Unform application of 35 kg N ha^{-1} annum^{-1}
Grazing management	· All cattle- 1 Jan to 31 Dec graze paddocks 1 and 2, withhold 14 days, check every 7 days and move when weight gain margin is > 0.01 kg day^{-1}
Soils	· All paddocks soil texture defined from Atlas in GrassGro, corresponding to a Northcote Dy5.61 classification[20]<br>· Paddock 1 & 2 FS 0.85
Tree plantings	· No environmental plantings above what currently on farm

Supplementary table 16. Measured tree carbon sequestration as a function of tree age in zones of southern Australia having more than 660 mm year^{-1} precipitation. Plantations comprise more than 50% *Eucalyptus spp.* Adapted from Hobbs et al.[1] and Neumann et al.[2]

Species	Annual precipitation (> 660 mm yr^{-1})	Age	C seq (Mg C ha^{-1} yr^{-1})	CO_2e seq (CO_2e ha^{-1} yr^{-1})	Location
E. viminalis ssp. cygnetensis (80.1%), E. ovata var. (9.3%), E. camaldulensis var. (3.9%), E. cladocalyx (3.2%), E. obliqua (2.8%), Ac. dodonaeifolia (0.3%), Mel. gibbosa (0.2%), Al. verticillata (0.1%), Ac. retinodes (0.1%), Mel. uncinata (<0.1%)	660	11.6	12.6	46.1	Kangaroo Island
E. camaldulensis/viminalis, Ac. Retinodes	843	12.9	7.4	27.1	Adelaide & Mt Lofty Ranges
Eucalyptus globulus ssp. Globulus	826	13.9	13.8	50.6	Adelaide & Mt Lofty Ranges
E. camaldulensis var. (99.2%), Mel. decussata (0.5%), Mel. gibbosa (0.2%), Mel. uncinata (0.1%), Callistemon rugulosus (<0.1%)	660	14.6	12.3	45.2	Kangaroo Island
E. camaldulensis, Ac. retinodes	843	28.0	8.4	30.4	Adelaide & Mt Lofty Ranges
E. camaldulensis var. camaldulensis (95.0%), Banksia marginata (3.7%), Ac. melanoxylon (1.3%)	727	34.0	2.8	10.2	Adelaide & Mt Lofty Ranges
Eucalyptus leucoxylon ssp.	672	96.9	2.2	8.1	Adelaide & Mt Lofty Ranges
E. goniocalyx ssp. goniocalyx (56.6%), E. camaldulensis var. camaldulensis	727	120.0	2.2	8.0	Adelaide & Mt Lofty Ranges

Species	Annual precipitation (> 660 mm yr^{-1})	Age	C seq (Mg C ha^{-1} yr^{-1})	CO_2e seq (CO_2e ha^{-1} yr^{-1})	Location
(28.2%), E. fasciculosa (10.8%), Ac. pycnantha (2.9%), Al. verticillata (1.4%)					

Supplementary table 17. Measured carbon sequestration of trees with recorded ages in temperate zones of southern Australia having 400-660 mm year^{-1} precipitation. Plantations comprise less than 50% Eucalyptus spp. and native species. Adapted from Hobbs et al.[1] and Neumann et al.[2]

Species	Precipitation (>400 & < 660 mm yr^{-1})	Average canopy age	C seq (Mg C ha^{-1} yr^{-1})	CO_2e seq (CO_2e ha^{-1} yr^{-1})	Location
Ac. mearnsii (55.0%), Ac. melanoxylon (19.7%), E. viminalis ssp. cygnetensis (14.0%), E. leucoxylon ssp. (7.3%), E. fasciculosa (4.0%), Al. verticillata (0.2%)	578	11.9	7.8	28.5	South East
Dodonaea viscosa ssp. (24.5%), Ac. pycnantha (23.4%), Ac. ligulata (22.2%), Ac. wattsiana (10.3%), E. leptophylla (8.4%), E. cyanophylla (4.3%), E. socialis ssp. (2.9%), Ac. brachy botrya (1.7%), Ac. anceps (1.0%), Al. verticillata (0.7%), Ac. notabilis (0.7%)	418	13.4	1.0	3.5	Kangaroo Island
E. leucoxylon ssp. (32.3%), Ac. mearnsii (30.6%), E. fasciculosa (12.5%), E. ovata var. (12.0%), Ac. pycnantha (12.0%), Mel. lanceolata (0.5%), Callistemon rugulosus (0.1%), E. gracilis (0.1%), E. incrassata (<0.1%)	507	14.8	6.9	25.2	South East
Corymbia maculata (50.5%), E. fasciculosa (30.2%), Ac. retinodes (13.1%), E. viminalis ssp. cygnetensis (4.8%), Ac. pycnantha (1.1%), Al. verticillata (0.3%), Mel. lanceolata (<0.1%)	592	14.9	2.6	9.5	Adelaide & Mt Lofty Ranges
Casuarina cunninghamiana (66.1%), E. cladocalyx (17.8%), E. leucoxylon ssp.	515	14.9	0.8	2.8	SA Murray-Darling Basin

Species	Precipitation (>400 & < 660 mm yr^{-1})	Average canopy age	C seq (Mg C ha^{-1} yr^{-1})	CO_2e seq (CO_2e ha^{-1} yr^{-1})	Location
(7.5%), Corymbia maculata (4.0%), Ac. pycnantha (2.4%), Al. verticillata (2.3%)					
Al. verticillata (99.5%), E. cneorifolia (0.4%), E. diversifolia ssp. diversifolia (0.1%)	503	16.6	0.9	3.4	Kangaroo Island
Al. verticillata (44.1%), E. fasciculosa (21.2%), E. diversifolia ssp. diversifolia (18.2%), E. cneorifolia (6.9%), Mel. halmaturorum (6.7%), E. cosmophylla (1.5%), Mel. gibbosa (1.3%), Ac. retinodes var. uncifolia (0.1%)	503	16.6	1.7	6.0	Kangaroo Island
E. porosa (47.2%), E. odorata (24.0%), Callitris gracilis (8.4%), Ac. notabilis (7.4%), E. incrassata (4.3%), Ac. oswaldii (3.3%), Ac. brachybotrya (2.1%), Ac. sclerophylla var. sclerophylla (1.4%), E. socialis ssp. (0.8%), Ac. acinacea (0.7%), Ac. ligulata (0.3%), Mel. lanceolata (0.1%)	414	18.8	0.6	2.0	Adelaide & Mt Lofty Ranges
**Non-Eucalypt species present**					
Corymbia maculata	492	6.9	3.9	14.3	SA Murray-Darling Basin
Callitris gracilis	478	7.4	0.1	0.3	SA Murray-Darling Basin
Corymbia maculata	495	7.4	1.1	4.0	SA Murray-Darling Basin
Ac. implexa	478	7.4	1.9	6.9	SA Murray-Darling Basin
Corymbia maculata	655	8.4	2.8	10.3	SA Murray-Darling Basin

Species	Precipitation (>400 & < 660 mm yr^{-1})	Average canopy age	C seq (Mg C ha^{-1} yr^{-1})	CO_2e seq (CO_2e ha^{-1} yr^{-1})	Location
Corymbia maculata	492	10.8	1.8	6.6	SA Murray-Darling Basin
Allocasuarina verticilllata	492	10.9	3.1	11.3	SA Murray-Darling Basin
Ac. mearnsii	492	12.5	9.2	33.8	SA Murray-Darling Basin
Casuarina cunninghamiana	585	14.9	0.7	2.6	SA Murray-Darling Basin
Casuarina cunninghamiana	585	14.9	0.9	3.5	SA Murray-Darling Basin
Casuarina cunninghamiana	465	14.9	1.2	4.3	SA Murray-Darling Basin
Allocasuarina verticilllata	403	33.0	0.4	1.4	SA Murray-Darling Basin

Supplementary table 18. Fitted parameters and market price distributions for beef cattle (Australian cents kg^{-1} dressed weight).

Units	One year old Heifers	Mixed age cows	Steers	MSA steers	Surplus heifers and feeder heifers	Cull cows
Probability Distribution	Pert	Pert	Pert	Pert	Log-normal	Pert
Min	300	300	300	300	-	300
Max	900	700	900	850	-	700
M. Likely	530	500	530	500	-	500
Mean	-	-	-	-	500	-
Std. Deviation	-	-	-	-	350	-

Supplementary table 19. Fitted parameters and market price distributions for wool production (Australian cents kg^{-1} clean fleece weight).

Units	Lambs (16 µm)	Ewes (18 µm)	Wethers (18 µm)	Merino Ram (18 µm)
Probability Distribution	Pert	Pert	Pert	Pert
Min	1400	1385	1385	1385
Max	2500	2300	2300	2300
M. Likely	1700	1636	1636	1636

Supplementary table 20. Fitted parameters and market price distributions for sheep meat (Australian dollars kg^{-1} dressed weight).

**Units**	**Lambs at 6 months**	**Prime lambs**	**Wethers**	**Rams**	**Cull ewes**
Probability Distribution	Pert[a]	Pert	Pert	Pert	Pert
Min	3.00	3.40	1.50	1.50	1.50
Max	8.00	8.00	5.93	5.93	5.93
M. Likely	5.40	5.70	3.45	3.45	3.45

[a] Family of continuous probability distributions defined by maximum, minimum and most likely values

Supplementary References

1 Hobbs T, Neumann C, Tucker M, Ryan K. Carbon sequestration from revegetation: South Australian agricultural regions. *Adelaide: Department of Environment, Water and Natural Resources, The Government of South Australia & Future Farm Industries Cooperative Research Centre*, (2013).

2 Neumann CR, Hobbs TJ, Tucker M. Carbon sequestration and biomass production rates from agroforestry in lower rainfall zones (300-650 mm) of South Australia: Southern Murray-Darling Basin Region. Adelaide & Future Farm Industries Cooperative Research Centre 32, (2011).

3 Bilotto F, Christie-Whitehead KM, Barnes N, Harrison MT. Operationalising net-zero with biochar: Black gold or red herring? *Trends Food Sci. Technol.* **150**, 104579 (2024). https://doi.org/10.1016/j.tifs.2024.104579

4 Coleman, K. & Jenkinson, D. RothC: a model for the turnover of soil carbon model description and user guide. *Rothamsted Research, Harpenden, UK* (2014). Available at: https://www.rothamsted.ac.uk/sites/default/files/RothC_guide_WIN.pdf

5 Morais, T. G., Teixeira, R. F. M. & Domingos, T. Detailed global modelling of soil organic carbon in cropland, grassland and forest soils. *PloS one* **14**, e0222604-e0222604 (2019). https://doi.org/10.1371/journal.pone.0222604

6 Hoogendoorn, C. J., Bowatte, S. & Tillman, R. W. Simple models of carbon and nitrogen cycling in New Zealand hill country pastures: exploring impacts of intensification on soil C and N pools. *New Zealand J. Agric. Res.* **54**, 221-249 (2011). https://doi.org/10.1080/00288233.2011.599395

7 Poeplau, C. Estimating root: shoot ratio and soil carbon inputs in temperate grasslands with the RothC model. *Plant Soil* **407**, 293-305 (2016). https://doi.org/10.1007/s11104-016-3017-8

8 Lefebvre, D. *et al.* Modelling the potential for soil carbon sequestration using biochar from sugarcane residues in Brazil. *Sci. Rep.* **10**, 19479 (2020). https://doi.org/10.1038/s41598-020-76470-y

9 Pulcher, R., Balugani, E., Ventura, M., Greggio, N. & Marazza, D. Inclusion of biochar in a C dynamics model based on observations from an 8-year field experiment. *SOIL* **8**, 199-211 (2022). https://doi.org/10.5194/soil-8-199-2022

10 Fernandez, M. Fit-for-purpose biochar to improve efficiency in ruminants. Meat & Livestock Australia Limited (2020). Retrievable at: https://www.mla.com.au/contentassets/a28c19322ec049e795b2695613553d26/bgbp_0032-biochar-final-report_mla-website-.pdf.

11 Goward, J. & Whitty, M. Estimating and predicting carbon sequestration in a vineyard using precision viticulture techniques. In Proceedings of the 19th Association of Public Authority Surveyors Conference (APAS2014), Pokolbin, NSW. (2014). Available at: https://www.apas.org.au/files/conferences/2014/Estimating-and-Predicting-Carbon-Sequestration-in-a-Vineyard-using-Precision-Viticulture-Techniques.pdf.

12 Tas Farming Futures. RMCG case study for Moores Hill Estate. (2023). Available at: https://www.tasfarmingfutures.com.au/carbon-neutral .

13 Australian Wine Research Institute. Nitrogen fertilisation Viti-Notes. (2010). Available at: https://www.awri.com.au/wp-content/uploads/1_nutrition_nitrogen_fertilisation.pdf.

14 Longbottom, M. L. & Petrie, P. R. Role of vineyard practices in generating and mitigating greenhouse gas emissions. *Aust. J. Grape Wine Res.* **21**, 522-536 (2015). https://doi.org/10.1111/ajgw.12197

15 Rugani, B., Vázquez-Rowe, I., Benedetto, G. & Benetto, E. A comprehensive review of carbon footprint analysis as an extended environmental indicator in the wine sector. *J. Clean. Prod.* **54**, 61-77 (2013). https://doi.org/10.1016/j.jclepro.2013.04.036

16 Williams, J. N., Morandé, J. A., Vaghti, M. G., Medellín-Azuara, J. & Viers, J. H. Ecosystem services in vineyard landscapes: a focus on aboveground carbon storage and accumulation. *Carbon Balance Manag.* **15**, 23 (2020). https://doi.org/10.1186/s13021-020-00158-z
17 Francaviglia, R. *et al.* Changes in soil organic carbon and climate change – Application of the RothC model in agro-silvo-pastoral Mediterranean systems. *Agric. Syst.* **112**, 48-54 (2012). https://doi.org/10.1016/j.agsy.2012.07.001
18 Eldon, J. & Gershenson, A. Effects of Cultivation and Alternative Vineyard Management Practices on Soil Carbon Storage in Diverse Mediterranean Landscapes: A Review of the Literature. *Agroecol. Sustain. Food Syst.* **39**, 516-550 (2015). https://doi.org/10.1080/21683565.2015.1007407
19 White, R.E. What are the realistic expectations for making money out of carbon credits in vineyards? Australian and New Zealand Grapegrower and Winemaker 666, 38-40 (2019).
20 Northcote, K. A Factual Key for the Recognition of Australian Soils. 4th Edition, Rellim Technical Publishers, Glenside, South Australia. (1979).
21 Duarte, C. M., Wu, J., Xiao, X., Bruhn, A., Krause-Jensen, D. Can Seaweed Farming Play a Role in Climate Change Mitigation and Adaptation? *Front. Mar. Sci*. 4, (2017). https://doi.org/10.3389/fmars.2017.00100
22 Harrison, M. T., Christie, K. M., Rawnsley, R. P. & Eckard, R. J. Modelling pasture management and livestock genotype interventions to improve whole-farm productivity and reduce greenhouse gas emissions intensities. *Anim. Prod. Sci.* **54**, 2018-2028 (2014). https://doi.org/10.1071/AN14421
23 Hayes, R. C. *et al.* Prospects for improving perennial legume persistence in mixed grazed pastures of south-eastern Australia, with particular reference to white clover. *Crop Pasture Sci.* **70**, 1141-1162 (2019). https://doi.org/10.1071/CP19063
24 Alcock, D. J. & Hegarty, R. S. Potential effects of animal management and genetic improvement on enteric methane emissions, emissions intensity and productivity of sheep enterprises at Cowra, Australia. *Anim. Feed Sci. Technol.* **166-167**, 749-760 (2011). https://doi.org/10.1016/j.anifeedsci.2011.04.053
25 Alford, A. R. *et al.* The impact of breeding to reduce residual feed intake on enteric methane emissions from the Australian beef industry. *Aust. J. Exp. Agric.* **46**, 813-820 (2006). https://doi.org/10.1071/EA05300
26 Reisinger, A. *et al.* How necessary and feasible are reductions of methane emissions from livestock to support stringent temperature goals? *Philos. Trans. A Math. Phys. Eng. Sci.* **379**, 20200452 (2021). https://doi.org/10.1098/rsta.2020.0452
27 Moore, A. D., Donnelly, J. R. & Freer, M. GRAZPLAN: Decision support systems for Australian grazing enterprises. III. Pasture growth and soil moisture submodels, and the GrassGro DSS. *Agric. Syst.* **55**, 535-582 (1997). https://doi.org/10.1016/S0308-521X(97)00023-1
28 Richards, G. P. & Evans, D. M. W. Development of a carbon accounting model (FullCAM Vers. 1.0) for the Australian continent. *Aust. For.* **67**, 277-283 (2004). https://doi.org/10.1080/00049158.2004.10674947
29 ARK Energy. Western Plains wind farm. Available at: https://arkenergy.com.au/wind/western-plains-wind-farm/.
30 Roque, B. M. *et al.* Red seaweed (*Asparagopsis taxiformis*) supplementation reduces enteric methane by over 80 percent in beef steers. *PloS one* **16**, e0247820 (2021). https://doi.org/10.1371/journal.pone.0247820
31 Black, J. L., Davison, T. M. & Box, I. Methane Emissions from Ruminants in Australia: Mitigation Potential and Applicability of Mitigation Strategies. *Animals* **11**, 951 (2021). https://doi.org/10.3390/ani11040951
32 Li, X. *et al.* *Asparagopsis taxiformis* decreases enteric methane production from sheep. *Anim. Prod. Sci.* **58**, 681-688 (2018). https://doi.org/10.1071/AN15883

33 Harris, R. M. B., Love, P. T., Fox-Hughes, P., Remenyi, T. A. & L., B. N. An assessment of the viability of prescribed burning as a management tool under a changing climate - Stage 2, Technical Report, Antarctic Climate and Ecosystems Cooperative Research Centre, Hobart, Tasmania. (2019).
