# Supplementary material for: Costs of transitioning the livestock sector to net-zero emissions under future climates
Source: Nat Commun. 2025 Apr 23;16:3810. doi: 10.1038/s41467-025-59203-5 (PMC12019546; doi:10.1038/s41467-025-59203-5)
Supplement: Supplementary file 4 — Source Data [file 41467_2025_59203_MOESM4_ESM.zip › Source data file SUBMITTED 20 March 2025/Bilotto et al (2023) Costs of transitioning to net-zero emissions under future climates for criculation 16 Apr 2023.docx]

**Costs of transitioning to net-zero emissions under future climates**

Franco Bilotto^1,2^, Karen Michelle Christie-Whitehead^3^, Bill Malcolm^4^, Matthew Tom Harrison^1*^

*^1^ Tasmanian Institute of Agriculture, University of Tasmania, Newnham, Launceston, TAS, 7248, Australia*

*^2^ AgResearch, Grasslands Research Centre, Tennent Drive, Private Bag 11008, Palmerston North 4442, New Zealand*

*^3^ Tasmanian Institute of Agriculture, University of Tasmania, 16-20 Mooreville Rd, Burnie, TAS, 7320, Australia*

*^4^ Faculty of Veterinary and Agricultural Sciences, The University of Melbourne, Parkville, VIC 3010, Australia*

*Corresponding author: [matthew.harrison@utas.edu.au](mailto:matthew.harrison@utas.edu.au)

**Abstract**

Contemporary land managers are faced with the trilemma of raising agri-food production, mitigating greenhouse gas (GHG) and/or conservation of natural resources. Here, we co-designed and costed transdisciplinary pathways for transitioning farming systems to net-zero emissions under future climates. Few interventions enhanced productivity and profitability while also reducing GHG emissions. *Asparagopsis* as a feed supplement and planting trees enabled the greatest GHG mitigation under future climates (67-95%); climatic diversification and altering lambing/calving times yielded the greatest improvement in productivity (16-18%), while enterprise diversification (investment in wind turbines), pasture renovation and feed-conversion efficiency (FCE) were most conducive to improved profitability (17-39%). Interventions that were considered most adoptable by the group of expert practitioners often had the lowest mitigation potential. The changing climate also had significant implications for the extent of carbon removals, with mitigation potential soils and vegetation declining under future climates.

Stacking together tree plantings, transformational FCE and pasture renovation realised carbon neutrality but also improved productivity and profitability when appropriately contextualised. Serendiptiously, we showed that the most socially unacceptable option – continuing business as usual and purchasing carbon credits to offset net farm emissions – was also the most costly option. In contrast, stacking together interventions enabling improved pasture growth together with soil carbon sequestration, together with adoption of animal genotypes with greater FCE and planting small areas of trees relative to farm size, could negate farm emissions. We conclude that economic returns associated with transitioning to net-zero emissions vary widely (-XXX% to +YYY%), depending on whether or not interventions are stacked and/or may elicit productivity co-benefits.

For the beef farm, feeding of *Asparagopsis*, planting trees and TFCE were most promising (CN1 and CN2), facilitating not only carbon neutrality but also an XX% gain in productivity and possible 30% profitability gain under 2050 climates (Fig. 3). For the sheep farm, productivity and profitability gains associated with carbon neutral GHG positions were more likely to be realised with stacking of *Asparagopsis* feed, planting trees and renovating pastures with lucerne, such that CN3 and CN4 increased production and profit by XXX and YYY% relative to the baselines, respectively (Fig. 4).

We conclude that stacking several contextualised interventions that enable carbon sequestration, enteric methane mitigation and productivity co-benefits will more likely result in sustainable avenues for transitioning farming systems to net-zero emissions and beyond.

**Keywords**

Climate emergency, Net-Zero 2050, Nationally Determined Contribution, Carbon Storage, Soil Carbon, Food Security, Environmental Stewardship, Social licence to operate, adoptability

**Introduction**

Increasing atmospheric greenhouse gases concentrations (GHG) evoke global warming, intensifying the global water cycle and increasing the risk of extreme events (Liu et al. 2020; Langworthy et al. 2018). During the last four decades, the frequency of natural disasters borne by extreme events has almost quadrupled, causing more than US$280B of losses in crop and livestock production^1^. However, regional variation in the effects of climate change, including seasonal and regional patterns of precipitation may - in some cases - realise benefits, such as reduced frequencies of extreme cold (Harrison 2021) and reduced waterlogging in arable landscapes (Liu et al. 2023).

Carefully conceived adaptations may enable food systems transformation, but often only if due consideration is given to a wide range of socioeconomic, institutional and cultural factors in the co-design process^5^. As a corollary, few *bona fide* examples of food systems transformations exist, perhaps because research has traditionally progressed in a reductionist fashion, with primarily unidisciplinary and isolated foci. Research designed to address only one GHG emissions reduction intervention has given rise to a phenomenon called ‘Carbon Myopia’, representing studies in which singular interventions are evaluated and rated based primarily on carbon removals or GHG emissions avoidance (Harrison et al. 2021). Effects of, or interactions caused by, such interventions on or with extraneous factors, such as prosperity, productivity, environmental stewardship and social licence, are often downplayed or ignored completely, even though such collectively factors determine the whether an intervention will be sustainable, and ultimately, successful (Ara et al. 2021; Taylor et al. 2016; Harrison et al. 2021). Compared with unidisciplinary approaches however, multi- and transdisciplinary work (cross discipline and cross institutional, respectively) tends to be more difficult to lead, and more costly in time and money to execute, and hence the majority of GHG emissions mitigation research continues to progress in siloed pockets^7,8^.

The bulk of past climate change adaptation and mitigation work for the livestock sector has however been premised primarily upon biophysical lenses. Such studies have examined, for example, (1) evaluation of GHG emissions of cropping and livestock systems (Meier et al. 2020); (2) comparisons of GHG emissions from model ensembles (Sandor et al. 2020), (3) and the influence of genotype by management by environment combinations on GHG emissions and productivity (Cottle et al. 2016; Yan et al. 2021; Ibrahim et al. 2019). Much less work for sheep and beef systems has focused on how interventions aimed at adaptation and/or mitigation influence productivity, profitability and GHG emissions, although similar efforts for other sectors (such as dairy and grains) indicate that conclusions drawn differ considerably when economics are also taken into account (Harrison et al. 2017; Monjardino et al. 2022). While land managers have a multitude of opportunities to reduce GHG emissions (e.g. through carbon removals, GHG emissions avoidance, or GHG emissions mitigation), scientific literature that develops, contrasts and economically costs pathways to carbon neutral farming systems is scarce. Here, our aims we thus to (1) co-develop a range of management, genetic, environmental, livestock and landscape interventions for both adapting livestock systems while reducing GHG emissions and (2) analytically cost (economically and biophysically) a range of plausible pathways to net-zero emissions. In evaluating these aims, we co-designed interventions with a ‘regional reference group’ (RRG) of industry experts and practitioners to ensure relevance, credibility and legitimacy of our proposed adaptation/mitigation interventions (Shahpari et al. 2021). We calibrate our models and social research using two real farms in southern Australia, refining analytical methods based on feedback from the RRG. We then explore the impact of singular and stacked (bundled) interventions on productivity, profitability, GHG emissions and adoptability (Harrison et al. 2016). Stacked interventions were categorised into groups based on similarity of intent, including ‘Low Hanging Fruit’ (simple, reversible, immediate changes that could be made to the farm system), ‘Towards Carbon Neutral’ (interventions primarily designed to reduce GHG emissions), Income Diversification (enabling revenue generation from enterprises other than livestock to reduce dependence on rainfall as a primary source of income) and others. While we exemplify our methods for two use cases, the approach could be generically adapted to any location, production system or transdisciplinary problem.

**The nexus between productivity, profitability and net greenhouse gas emissions**

In comparing sheep and beef production systems in 2030 and 2050, we reveal that (1) few individual interventions elicited significant impact on the three dimensions of productivity, profitability and GHG emissions and (2) the impacts of production system and intervention were greater than the impacts of climate change *per se* (Figs 1, 2).

Interventions targeting livestock enteric methane (methane produced by fermentation in the gut) were most promising in terms of putting a deep cut in GHG emissions, such as the seaweed feed additive *Asparagopsis taxiformis*, which reduced on-farm CO_2_-eq by 46-72% under future climates (Fig. 1a, 1b, Fig. 2a, 2b, Tables S1-S4). However, *Asparagopsis* when used as a feed supplement decreased profits by $23-25/Mg CO_2_e mitigated (Fig. 1c, 1d, Fig. 2c, 2d), and was one of the most costly singular interventions examined. Interventions that we considered most adoptable by the group of expert practitioners (the RRG) often had the lowest mitigation potential (Figs 1, 2).

Climatic diversification - purchasing a farm in a distinctively different climatic zone - and altering lambing/calving times yielded the greatest improvement in productivity (16-18%), while enterprise diversification (capital investment to enable grapevine/wind turbines enterprises), pasture renovation with deep-rooted legumes and improvements in animal genetic feed-conversion efficiency (FCE) were most conducive to improved profitability (17-39%). Interventions enabling the greatest gains in productivity and profitability tended to have little influence on GHG emissions mitigation however, underlining the recalcitrant coupling between GHG emissions and productivity.

Improving FCE - considered akin to good farm management practice by increasing pasture utilisation and liveweight gain per unit utilisation in a sustainable way – significantly raised profitability ($50-300/Mg CO_2_e mitigation; Fig. 1c, 1d, Fig. 2c, 2d, Tables S1-S4) and productivity (XXXX percentage gains here), although had more modest impacts on GHG emissions mitigation (XXXX effects on GHG). Transformational improvement in animal genetic feed conversion efficiencies was highly prospective (TFCE; raising baseline FCE by more than 30%) enhancing livestock production and farm profits by 8-39% and reducing net GHG emissions by 11-17%, although was considered less adoptable by the expert group of practitioners due to the prerequisite science on livestock genetics that would need to take place to improve FCE before such genotypes could be purchased (Fig. 3a, 3d, Fig. 4a, 4d).

The changing climate had significant implications for the extent of carbon removals. By 2050, GHG mitigation potential associated with improving soil carbon stocks was reduced by 6-13% for interventions that expanded farm area covered by deep-rooted perennial legumes (in this case lucerne or *Medicago sativa*), and by 20-40% for carbon sequestered by planting native vegetation (Figs 1, 2, Tables S1-S4). Planting trees had greater detrimental impact on profitability per unit CO_2_ mitigated compared with incorporation of lucerne into pastures (Fig. 1c, 1d and Fig. 2c, 2d), because lucerne enabled pasture growth and thus livestock production, whereas trees negated productive pasture area and thus livestock carrying capacity.


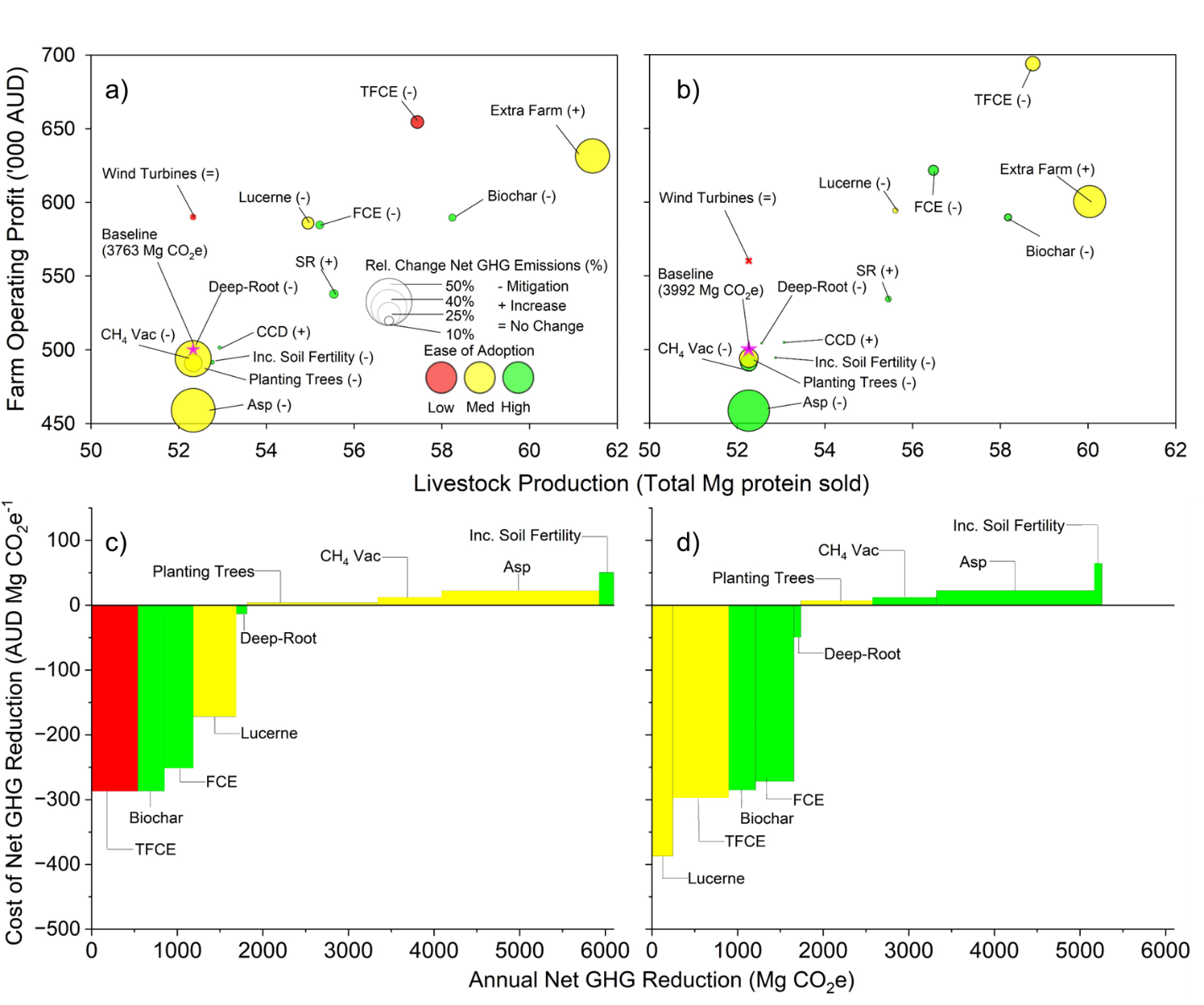


**Fig. 1.** Production, operating profit, adoptability, mitigation potential (a and b) and marginal abatement cost curves (c and d) of multiple thematic adaptation/mitigation intervention/s for a beef farming system. Interventions were co-designed with a Regional Reference Group of expert practitioners for 2030 (a and c) and 2050 (b and d) climates. Purple star depicts the baseline scenario. Total emissions for the baseline scenario shown in parenthesis in (a) and (b). Asp: Asparagopsis taxiformis as a feed supplement; CH4 vac: injecting animals with an enteric CH4 inhibitor vaccine; CCD: changing calving date; Deep-Root: increasing pasture sward root depth with perennial legume renovation; FCE: increasing livestock feed conversion efficiency; SR: increasing stocking rate; TFCE: transformational increases in livestock feed conversion efficiency.

**
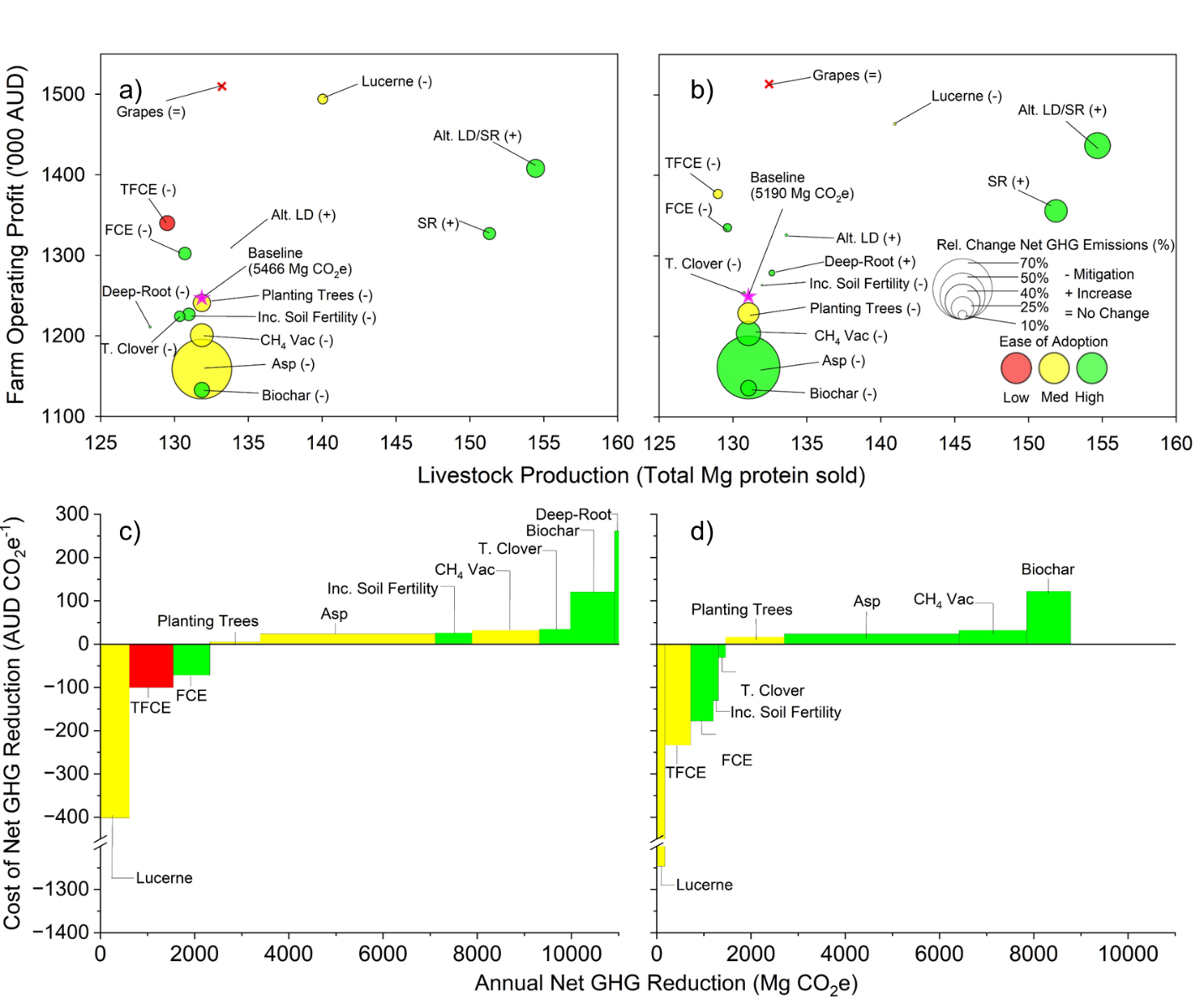
**

**Fig. 2.** Production, operating profit, adoptability, mitigation potential (a and b) and marginal abatement cost curves (c and d) of multiple thematic adaptation/mitigation intervention/s for a sheep farming system. Interventions were co-designed with a Regional Reference Group of expert practitioners for 2030 (a and c) and 2050 (b and d) climates. Purple star depicts the baseline scenario. Total emissions for the baseline scenario shown in parenthesis in (a) and (b). Asp: Asparagopsis taxiformis as a feed supplement; CH4 vac: injecting animals with an enteric CH4 inhibitor vaccine; CCD: changing calving date; Deep-Root: increasing pasture sward root depth with perennial legume renovation; FCE: increasing livestock feed conversion efficiency; SR: increasing stocking rate; TFCE: transformational increases in livestock feed conversion efficiency.

Considered highly adoptable by the RRG, biochar as a livestock feed supplement was proposed based on anecdotal evidence suggesting that use of biochar (1) improved liveweight gain, (2) reduced enteric methane and (3) enriched organic carbon content of manure. In line with the people-centric nature of this research, we conducted on-farm experiments with free-choice biochar, fed *ad libitum* over 12 months but revealed little impact of biochar on either liveweight gains or manure organic carbon content (Supplementary Figure/Table XXXX). Embedding these nascent results into our modelling frameworks showed that biochar feed supplement reduced net GHG emissions by 8% and increased profit by 18%, saving $290 Mg CO_2_e^-1^ per year (Fig. 1). However, effects of feeding biochar differed across production systems (cf. Fig. 1 c, d with Fig. 2c, d), with *de minimus* effects of biochar feed supplement on sheep liveweight gains and wool production along with elevated costs of implementation reducing profits by 10% despite an 18% reduction in GHG emissions for both climate horizons (Fig. 2).

To buffer against the possibility of reduced rainfall under future climates, income diversification avenues that were independent of rainfall in the one location were co-designed. These interventions included planting a small irrigated area of grapevines on the sheep farm, hosting wind turbines on the beef farm, and climatic diversification by purchasing a block of land for cattle farming in a distinctively different climatic zone. While wind turbines, developing irrigated grapevines and purchasing another beef cattle farm improved farm profits by 12-18%, 20% and 15% respectively (Figs 1 and 2), effects on productivity and profitability varied widely. Buying an extra beef farm in a diverse agro-climatic region improved production by 15% (Fig. 1), but this came with a cost of increased GHG emissions (net and emissions intensity, Tables S1-S2).

The RRG further underscored the low adoptability of all income diversification interventions. For example, purchasing a farm in a diversified climatic zone (north-eastern Tasmania, compared with the beef farm that was located some 400 km away in the north-west of the state) was perceived to require additional labour, trucks for transporting cattle between regions, infrastructure on the new farm, and effort in harmonising coordination of separate farm enterprises. Growing irrigated grapevines was thought to require specialist input to survey microclimates so to locate the vines in the most climatically convenient position (minimising risk of frost etc), while wind turbines required proximity to three-phase powerlines (to feed into the main electricity grid) as well as a baseline environment characterised by high prevailing windspeeds. Despite this however, the sheep case study farmer was indeed pursuing investment in irrigated grapevines, while the beef farmer had signed a lease for a company to lease part of his land for wind turbines.

**Contextualised adaptation-mitigation bundles: stacking interventions**

We next co-designed and stacked together contextualised bundles of inteventions, each group based on synergies of outcome intended (Figs 3 and 4). Simple, immediately actionable and relatively reversible were stacked together into a ‘Low Hanging Fruit (LHF)’ theme that (1) improved annual productivity (XXXX%), (2) increased profit by 14-20% but increased GHG emissions by XXXX% compared with the baseline scenarios under future climates.

A Towards Carbon Neutral (TCN) package was co-designed with the intent of improving productivity and reducing year-on-year GHG emissions by combining the LHF package with mitigation interventions (methane inhibition vaccine, planting trees and renovating pastures with deep-rooted legumes). The TCN package respectively increased livestock productivity by 18-20% (beef farm) and by 36-40% (sheep farm) under future climates (Tables S5-S8). Despite economic costs associated with tree planting and CH_4_ vaccine inoculation (Table S9), biophysical changes realised from pasture renovation increased profits by 33-37% and 60-68% for the beef and sheep farms, respectively. The TCN package reduced net GHG emissions by 37-69% for the beef farm (Fig. 3) and 29-34% for the sheep farm (Fig. 4), diluting emission intensities by 30-50% (Tables S5-S8). While the TCN package was highly ranked in terms of profit, production and GHG emissions evidenced by equally distributed ternary plots (Fig. 3c, 3f, Fig. 4c, 4f), the incorporation of strategies such as the methane inhibition vaccine (which is not commercially available) reduced the adoptability of TCN overall.

We revealed that multiple combinations of stacked interventions facilitated profitable transitioning of farm systems to net-zero emissions (Figs 3ad; 4ad). The four carbon neutral packages (CN1-4) were co-designed with consideration to various areas of trees planting, adoption (or not) of livestock genotypes with transformational gains in FCE (TFCE) and/or renovation of pastures with the deep-rooted perennial legume, lucerne. For the beef farm, feeding of *Asparagopsis*, planting trees and TFCE were most promising (CN1 and CN2), facilitating not only carbon neutrality but also an XX% gain in productivity and possible 30% profitability gain under 2050 climates (Fig. 3). For the sheep farm, productivity and profitability gains associated with carbon neutral GHG positions were more likely to be realised with stacking of *Asparagopsis* feed, planting trees and renovating pastures with lucerne, such that CN3 and CN4 increased production and profit by XXX and YYY% relative to the baselines, respectively (Fig. 4).

**
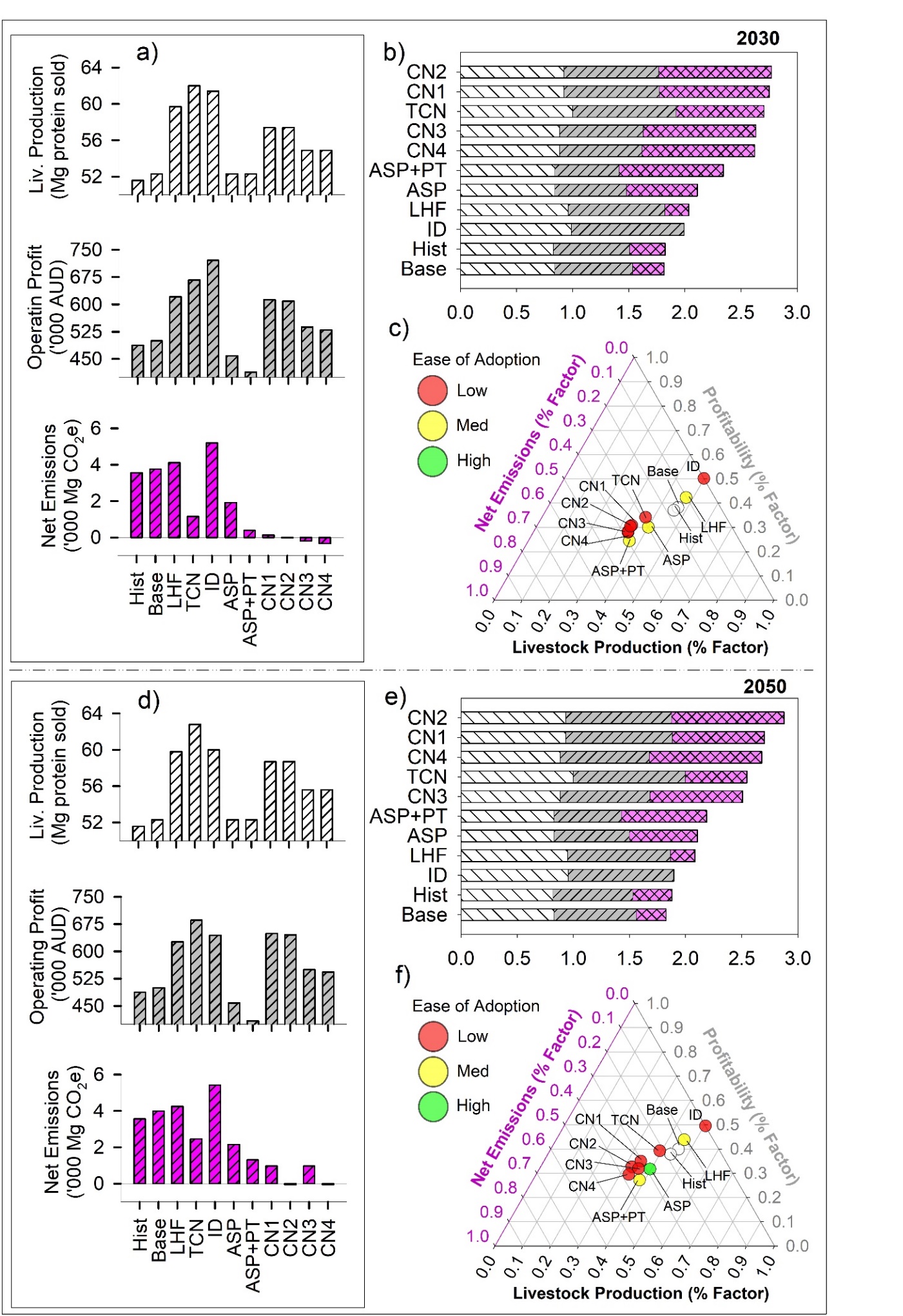
**

**Fig. 3.** Multidimensional assessment of co-designed thematic adaptations for a beef case study farm under 2030 and 2050 climate horizons. Hist: historical climates; Base: existing farming system under future climates; LHF: low-hanging fruit package; TCN: towards carbon neutral package; ID: income diversification; Asp: Asparagopsis taxiformis as a feed supplement; Asp + PT, Asp + planting 50 ha trees; TFCE, adopting livestock genotypes with transformational feed conversion efficiency; CN1: carbon neutral package 1 (Asp + TFCE + planting 50 ha trees), CN2: carbon neutral package 2 (Asp + TFCE + 55 ha trees 2030 and 80 ha trees 2050), CN3: carbon neutral package 3 (Asp + renovating pastures with lucerne + planting 50 ha trees); CN4: carbon neutral package 4 (Asp + renovating pastures with lucerne + 55 ha trees 2030 and 80 ha trees 2050).

**Costs of transitioning to net-zero emissions under future climates**

We next assumed a carbon market that taxed or credited on-farm GHG emissions and offsets, respectively. Payment of carbon taxes on net GHG emissions with no practice changes to reduce GHG emissions reduced farm profits by 64% and 33% for the beef and sheep farms, respectively (Fig. 5). While use of *Asparagopsis* as a feed supplement decreased operating profit by 7-8%, implementation of a carbon tax on residual GHG emissions improved profit by 58% (beef farm) or 25% (sheep farm) relative to the baseline farm in which all net GHG emissions were taxed (Fig. 5c, d, g, h). When feeding of *Asparagopsis* was stacked with purchasing an extra farm that was planted with trees (ASP+PT), a further 38-87% net GHG emissions were offset (Fig. 5a, b, e, f). Relative to the baseline farm in which all residual GHG emissions were taxed, ASP+PT improved profits by 34%/68% for the sheep/beef farm.

CN packages stacked TFCE (CN1 and CN2) or lucerne in the pasture mix (CN3 and CN4) with ASP+PT to synergistically reduce GHG emissions while further improving post-carbon taxes. For the beef farm, there was little difference in net GHG emissions after implementing TFCE (CN1) and lucerne in the pasture sward (CN3), both with residual GHG emissions of 1,000 Mg CO_2_e (Fig. 5a, b). Post-carbon tax profits were greater for the CN1 package (Fig. 5c) compared with the CN3 package (Fig. 5d), and were three times greater than the baseline farm, even after paying a tax on residual GHG emissions. Additional land for tree plantings was required for the beef farm’s CN1 and CN3 packages to become net-zero (CN2 and CN4 packages; Fig. 5a, b). For the sheep farm, the lucerne CN3 package achieved net-zero, with net sequestration of 1,400 Mg CO_2_e (Fig. 5f) and pre-carbon tax profit of $1,366K (Fig. 5h), which slightly declined if surplus carbon offsets were sold (Fig. 5h).

While the RRG highlighted potential difficulties in implementing CN packages (Table S10), our results clearly demonstrate that adoption of mitigation practices were at least three times more profitable for the beef farm and 1.5 times more profitable for the sheep farm, relative to farming systems that conducted business as usual and had all net GHG emissions subjected to carbon taxes.

**
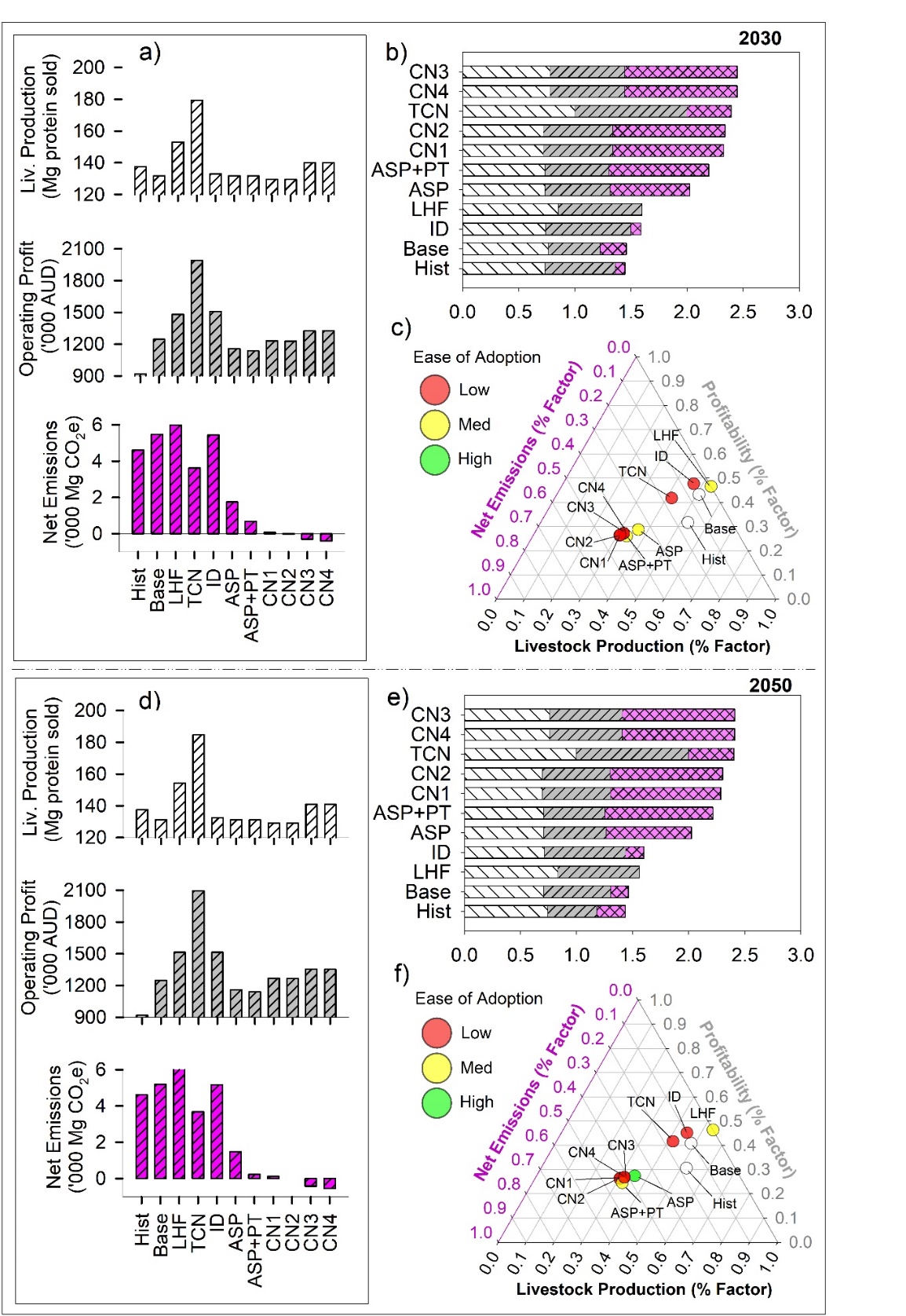
**

**Fig. 4.** Multidimensional assessment of co-designed thematic adaptations for a sheep case study farm under 2030 and 2050 climate horizons. Hist: historical climates; Base: existing farming system under future climates; LHF: low-hanging fruit package; TCN: towards carbon neutral package; ID: income diversification; Asp: Asparagopsis taxiformis as a feed supplement; TFCE, adopting livestock genotypes with transformational feed conversion efficiency; Asp + PT, Asp + planting 200 ha trees; CN1: carbon neutral package 1 (Asp + TFCE + planting 200 ha trees), CN2: carbon neutral package 2 (Asp + TFCE + 220 ha trees), CN3: carbon neutral package 3 (Asp + renovating pastures with lucerne + planting 200 ha trees); CN4: carbon neutral package 4 (Asp+ renovating pastures with lucerne + 220 ha trees).

**Discussion**

**A new framework for co-designing bundles of mitigation-adaptation interventions**

Here we develop and apply a participatory framework that embodies the best available science for effective climate action, together with rigorous stakeholder engagement to minimise trade-offs in line with key outcomes of COP27^12^. Our people-centric framework engaged end-users directly or indirectly affected by the climate crisis to develop fit-for-purpose farm interventions and thematic innovation bundles^7,14^. The flexible design of this framework with the use of integrated, interchangeable and numerical and social systems thinking could be adapted to explore sustainability indicators associated with multuple analogous production systems, locations or climatic horizons. Participatory research in this way not only builds end-user confidence and likelihood of adoption, but engenders social licence to operate towards landholders through improved public good towards their agri-environmental stewardship^17^.

**The cost of transitioning farm systems to net-zero emissions**

Serendiptiously, we showed that the most socially unacceptable option – continuing business as usual and purchasing carbon credits to offset net farm emissions – was also the most costly option. In contrast, stacking together interventions enabling improved pasture growth together with soil carbon sequestration (e.g. renovation with lucerne), together with adoption of animal genotypes able to gain more weight on the same amount of feed (FCE, TFCE) and planting small areas of trees relative to farm size (50-200 ha in this case), could and did negate all farm emissions. Even better, GHG emissions mitigation interventions soliciting a productivity co-benefit – such as improved metabolisible energy supply per unit area with legumes, or shade and shelter provided by trees – was likely to facilitate both carbon neutrality as well as XXX-yyy% gains in productivity and profitability under future climates. While we classify this result as a win-win-win, other factors, such as practical barriers to adoption, new knowledge required to implement practice changes, consumer needs and expectations were not taken into account. These extraneous factors may influence adoptability, as shown by feedback from an expert group of practitioners on some of the mitigation/adaptation bundles here (e.g. purchasing and extra farm or leasing part of the land to wind turbine companies to generate electricity).

The need to buy carbon credits to offset farm GHG was reduced as additional international were stacked into the package, especially when such interventions catalysed animal performance (CN packages, Fig. 5). Our results demonstrate that livestock producers need to continually adapt to avoid, reduce and remove GHG emissions (e.g., through interventions such as enteric CH_4_ vaccines, feed additives such as *Asparagopsis*, breeding low CH_4_ emitting animals, nitrification inhibitors, balancing dietary energy to protein^7^), whilst offseting farm emissions surplus through revegetation combined with increasing SOC where possible. Purchasing additional land with the explicit objective of planting trees to offset livestock emissions and feeding a CH_4_ inhibitor such as *Asparagopsis* in combination with TFCE (CN1 and CN2) or lucerne (CN3 and CN4) in the pasture mix was a promising avenue for improving profit while also achieving carbon neutrality. As the need for non-agricultural industries to also offset their GHG emissions increases, the price of arable land is likely to increase in line with public pressure to maintain or improve institututional and organisational carbon removals^32^. As a corollarly, carbon insetting (practices to reduce GHG within the value chain) may become a higher priority for land managers, rather than seeking new arable land elsewhere. Our analyses demonstrate that CN packages with and without emission trading schemes were more profitable than baselines scenarios where existing and emerging technologies could deliver the necessary abatement to reach net zero by 2050, opening the door to new markets spurred by increasing consumer preferences for low carbon products^33^.

Tactical and strategic whole farm management plans should be flexible enough to include new available technologies, practices, and market and climatic trends (Muleke et al. 2022). To be effective in Nationally-Determined Contributions forests require a minimum level of ‘permanence’ (e.g. 25-100 years, depending on carbon market)^34^, potentially consuming arable zones that go a long way towards fulfilling the growing global need for protein, fibre and starch.

**
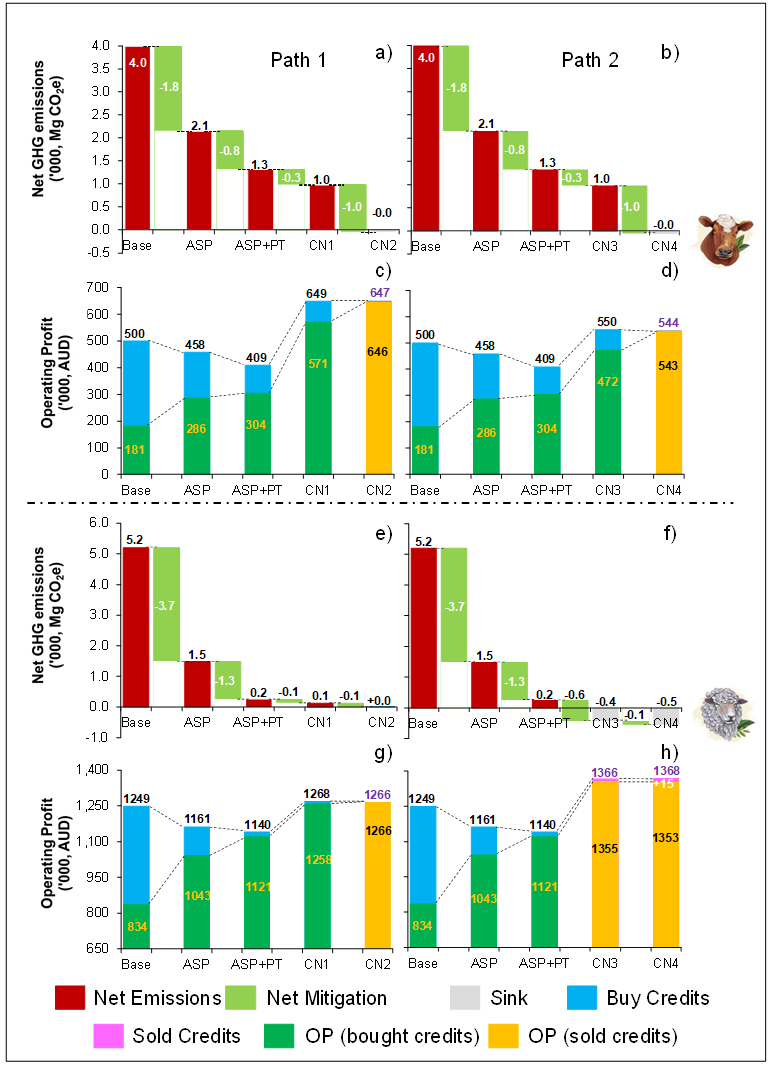
**

**Fig. 5.** Pathways to carbon neutrality (red and light green bars) with associated costs of pre-carbon taxes (blue and yellow bars) and post-carbon taxes (dark green and pink bars) across climate horizons and thematic adaptations for the beef farm (a, b, c and d) and sheep farm (e, f, g and h). Pathways 1 and 2 reflect net-zero farming systems attained by improving animal genetics (CN1 and CN2) or renovating pasture swards with lucerne (CN3 and CN4); Base: 2050 climates; ASP: Asparagopsis taxiformis as livestock feed supplement; Asp+PT: Asparagopsis taxiformis + planting trees (XX ha); CN1: carbon neutral package 1 [Asparagopsis taxiformis + planting trees 50 ha (beef farm) or 200 ha (sheep farm) + transformational feed conversion efficiency]; CN2: carbon neutral package 2 [Asparagopsis taxiformis + planting trees 85 ha (beef farm) or 220 ha (sheep farm) + transformational feed conversion efficiency]; CN3: carbon neutral package 3 [Asparagopsis taxiformis + planting trees 50 ha (beef farm) or 200 ha (sheep farm) + Lucerne]; CN4: carbon neutral package 4 [Asparagopsis taxiformis + planting trees 85 ha (beef farm) or 220 ha (sheep farm) + Lucerne]; OP: operating profit.

An *en masse* land-use conversion from commodity-based production to that designed for ecosystems services may have perverse outcomes, such as diminished food security or increase poverty; phenomena exacerbated by a burgeoning global population. Countries or regions that prioritise carbon and/or environmental outcomes may invoke carbon leakage^35^, wherein commodity-based production shifts to other regions or nations, potentially causing land clearing (e.g., substantial release of GHG emissions in developing nations in South America), and thus the atmosphere perceived more GHG emissions that would have occurred had the original land not been locked up for carbon or biodiversity purposes.

**Simple changes to systems raise productivity and profitability but further practices are required to reduce emissions to net-zero**

An important insight of our study was that CN packages simultaneously increased farm productivity and profitability while offsetting GHG emissions. Feeding livestock with *Asparagopsis* was the most promising adaptation*,* decreasing enteric CH_4_ emissions by 80% (46-72% reduction in total net GHG emissions on farm). However, we assumed high rates of enteric CH_4_ mitigation, and while our values are lower than some published results^18^, our CH_4_ mitigation quantum remains to be observed in practice. Projections for 2040 indicate a $1.5 billion seaweed industry in Australia, creating 9,000 jobs and up to 10% national GHG emissions reduction per year, making a substantial contribution to the UN Sustainable Development Goals^19^. However, several challenges remain before this future could be realised. Not least among which are whether species of seaweed for CH_4_ mitigation are invasive, whether when fed to animals bromoform (a potent CH_4_ inhibitor) may have contaminatory and/or carcinogenic implications for consumers, and whether synthetic bromoform manufacture at scale may impact on ozone depletion^18,20^.

Stacking of the transformational FCE (TFCE) into CN packages (CN1, CN2) increased animal performance in the beef farm and decreased costs of production (i.e. 50-88% reduction in supplementary feeding), improving profits (Figs 3, 4). However, reduced wool production associated with TFCE eroded overall livestock production for the sheep farm, similar to results seen by others^21^. On the other hand, lower pasture intakes may reduce enteric CH_4_ emissions and increase residual biomass and litter fall, potentially improving SOC stocks. Despite these prospective emergent economic and environmental complementarities, benefits elicted from genetic improvement have historically only been observed after 10-20 years of sustained investment^22,23^.

The expert group of practitioners we engaged showed great interest in the inclusion of legumes (e.g., lucerne and Talish clover) into existing grass pastures (CN3 and CN4 packages). Sturludóttir, et al. ^24^ demonstrated that mixing grasses with legumes improved herbage yield, dry matter digestibility and crude protein in pastures from Northern Europe and Canada, but also reduced the invasion of weeds compared to monocultures. The nitrogen yield advantage from grass-legume mixtures supported by symbiotic N_2_ fixation^25^, given the close linkage between C and N cycling in grazing systems^26^, could be though of as driving mechanisms of SOC stocks^27^. However, excessive proportions of legumes within swards come with animal welfare concerns, with excessive soluble protein and nitrate contents linked to ruminant bloat and even animal deaths^28^.

**Concluding remarks**

Here we reveal a number of unique insights:

1. Few singular interventions elicit improvements in productivity, profitability and GHG emissions;
2. Feeding *Asparagopsis* and planting trees had the greatest emissions benefits, but also came with the greatest costs;
3. Climatic diversification (purchasing and extra farm block in a unique climatic zone) as well as transformational improvements in animal feed-conversion efficiency (TFCE) solicited among the greatest benefits for productivity and profitability;
4. Stacking of interventions explicitly aimed at (1) reducing enteric methane, (2) carbon removals and (3) improving productivity were often the most profitable and productive, while also having the least GHG emissions;
5. Continuing business as usual and purchasing carbon credits so to negate all farm GHG emissions was most costly. This result is serendipitous, because, if adopted *en masse*, would see little carbon removed from the atmosphere, and thus little effect of practice changes on global warming;
6. Appropriately contextualised bundles of interventions (e.g. planting trees, renovating pastures with deep rooted legumes and adopting high FCE animal genotypes) not only reduced farm business GHG emissions to net zero, but also resulted in improved profitability and productivity gains;
7. We conclude that *transformational* solutions will be those that engender profitability, productivity, social licence to operate, low emissions or carbon removals and environmental stewardship. Not all of these interventions necessarily need to be implemented any given farm, but rather optimised across the landscape.

**Materials and methods**

**Study overview**

Farming systems were co-designed using integrated, cross-disciplinary framework^8^. A Regional Reference Group (RRG) of experts and industry practitioners was involved in the co-design of biophysical, environmental, and economic interventions (Fig. 6). Co-designed interventions (singularly and in combination) were further examined using a social science lens, including assessment of adoption barriers, social license to operate, and new skills needed to adopt them. A participatory process was used to quantify and stack individual whole farm adaptations on top of the baseline farm system, each intervention iteratively refined by discussing results with the RRG over several cycles.

To showcase this approach, farm systems across two regions of Tasmania, Australia, were selected: a sheep production system (hereafter ‘sheep farm’) in the low rainfall zone in central Tasmania and a beef production system (hereafter ‘beef farm’) in the relatively high rainfall zone of northwestern Tasmania. Individual interventions aimed at income diversification and/or transformational (30% improvement in each of production, profit and GHG emissions mitigation) were suggested by the RRG. Transformational adaptations were considered to be longer-term, higher risk interventions with some degree of irreversibility. These adaptations were stacked together in a mutually synergistic way based on commonality of intended outcomes. Incremental adaptations were defined as those that do not significantly alter the *status quo*. Income diversification interventions were designed such that new income streams would be derived that were independent of rainfall in the location of the current farm system, as rainfall was perceived to be a climatic index that would change under future climates, and these livestock systems relied primarily on pasture produced from rainfall. Income diversification was thus classified as those interventions affording either climatic diversification or enterprise diversification.

A multitude of approaches and software packages were used to simulate farm systems (Fig. 6). Future climate projections^17^ accounted for increased frequency and severity of extreme weather events. The whole-farm model GrassGro® (version 3.3.10^39^) was used to simulate daily pasture and livestock production and was driven by historical and future climate horizons. Soil organic carbon sequestration were simulated using RothC model (version 26.3 in Microsoft Excel format^40^) with GrassGro outputs, while FullCAM (version 4.1.6^41^) was invoked to estimate tree carbon sequestration. Net farm GHG emissions were calculated using Sheep Beef-Greenhouse Accounting Framework (SB-GAF version 1.4^42^) using outputs from GrassGro®, RothC and FullCAM. The @Risk model^43^ was used to account for market volatility using a partial budgeting approach (i.e. Earnings Before Interest and Taxes and herein referred to as operating profit or profit) to compare the costs and income benefits of incremental, income diversification and transformational adaptions faced by a farm business.


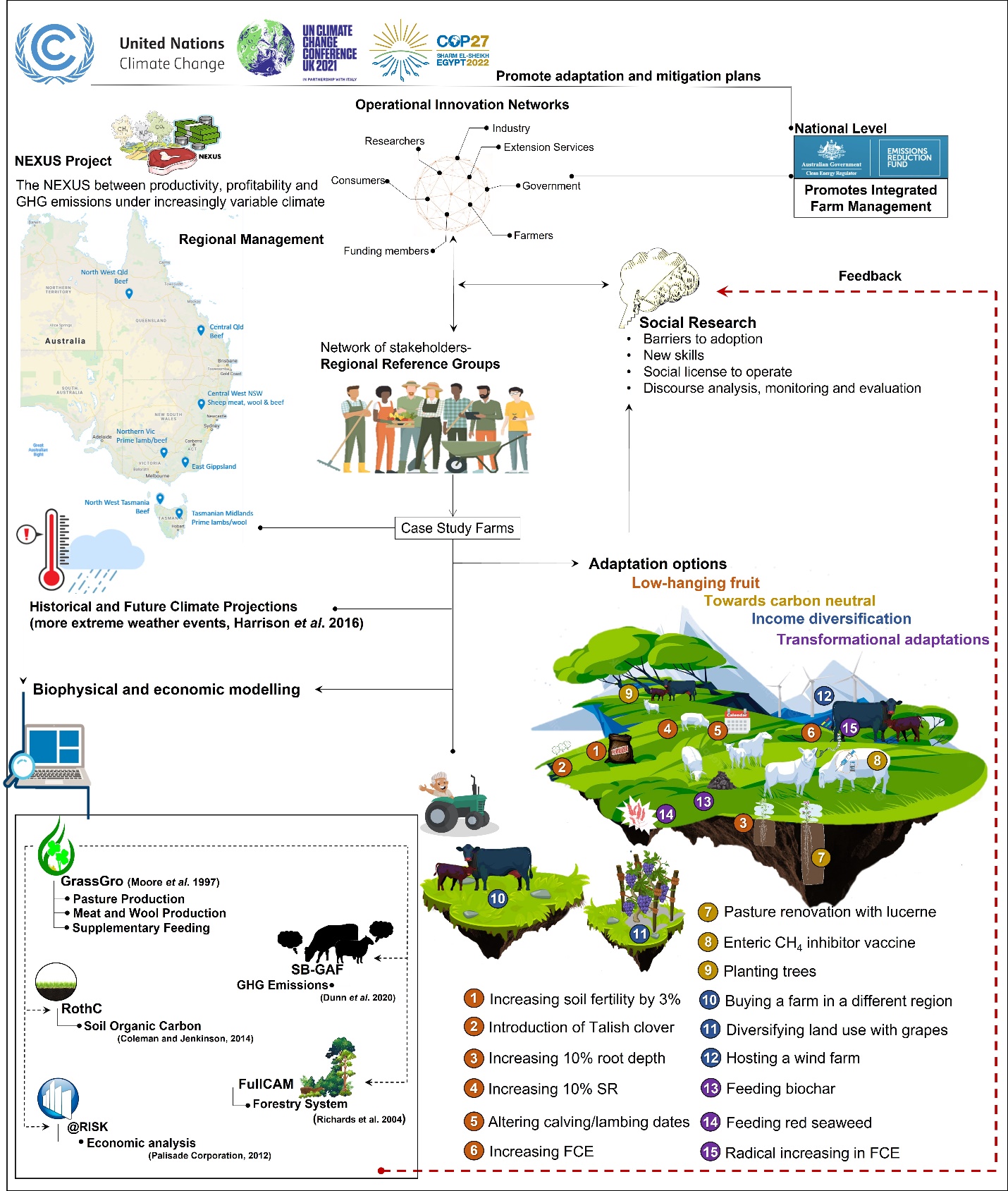


**Fig. 6.** Co-design climate change adaptation-mitigation framework for quantifying and examining relationship between productivity, profitability, GHG emissions, social acceptability and adoptability under historical and future climates. Orange, light brown, blue and purple circles represent Low-Hanging Fruit (LHF), Towards Carbon Neutral (TCN), Income Diversification (ID) and Transformational adaptation-mitigation themes, respectively.

**Historical and future climates**

The beef farm was located at Stanley in the cool temperate zone of north-western Tasmania (40° 43' 41''S 145° 15' 43''E), while the sheep farm was located in the Midlands, west of Campbell Town (41°56'30"S 147°25'02"E). Stanley and Campbell Town have long-term mean and standard deviation annual rainfall of 807 ± 139 mm and 499 ± 103 mm, respectively, with average daily temperatures of 16.5°C and 16.7°C in January and 9.1°C and 6.5°C in July, respectively (Fig. S1). Daily historical climate data for the baseline period of 1 January 1980 to 31 December 2018 was sourced from SILO meteorological archives (<http://www.longpaddock.qld.au/silo>). Data from SILO was used to generate future climate data following Harrison et al.^17^ using a stochastic approach to account for changes in climatic extremes, including heatwaves, droughts and extreme rainfall events^17^. Future climate projections were downscaled from global circulation models (GCMs) to regional and farm-scale^44^. To generate future climate data, (1) we estimated mean changes in future climates projected for a region based on ensembles of global climate models (GCMs), (2) accounted for historical climate characteristics (obviated by raw GCM data) and (3) generated climatic projections with increased variability. Future climate projections for 2030 and 2050 were developed using monthly regional climate scaling factors (Table S14) from GCMs provided by Harris et al.^44^ based on Representative Concentration Pathway (RCP) 8.5. Atmospheric CO_2_ concentrations were set at 350 ppm, 450 ppm and 530 ppm for the historical, 2030 and 2050 climate scenarios, respectively^45^.

**People-Centred Design: the Regional Reference Group (RRG)**

During an iterative process with a RRG, we sense-checked model assumptions and results. Model outputs discussed with the RRG included pasture growth rates, stocking rates, livestock production, wool production, supplementary feeding, costs, income, depreciation, net cash flows, and wealth. When RRG consensus was reached for results for each historical period (1986 to 2005), several biophysical and economic models were run for 26-year periods (first six years of data discarded to allow for model initialisation) centered on 2030 (2022 to 2041) and 2050 (2042 to 2061). Over several workshops, we gleaned RRG thinking and feedback on incremental, systems and transformational adaptation and mitigation opportunities in light of qualified holistic impacts of climate change. Assuming the recommendations from the RRG, we explore individual adaptations to understand their potential effects on productivity, profitability and offsetting of GHG emissions. Sequentially, several adaptations were combined into four distinct themes; ‘*Low Hanging Fruit*’, ‘*Towards Carbon Neutral*’, ‘*Income Diversification*’ and ‘*Carbon Neutral*’; outcomes from these themes were compared with the baseline scenario (detailed Fig. 5 and Table 1). Based on RRG advice, we refined model parameters to reflect feasibility and magnitude of variables simulated for each theme of adaptation. This process (1) ensured that model results were realistic, (2) provided the research team with nascent knowledge relating to opportunities for adaptation and mitigation of climate change from expert practitioners, (3) engender end-user confidence in the analytical process and results and (4), provided end-users with credible, legitimate and fit-for-purpose adaptation/mitigation interventions. Detailed information about the baselines and adaptation process is below and in the supplementary information (Tables S15-S18, Fig. S2).

**Pasture and livestock production**

The model GrassGro® enables simulation of ruminant grazing enterprises of southern Australia by combining biophysical (climate, soils, pastures and livestock), farm management (soil fertility, paddock size and layout, pasture grazing rotations, stocking rate and animal management) and economic data (gross margin). GrassGro® has been used to explore the effects of climate, soil, pasture, herd/flock management and adaptation for predicted climate change impacts on livestock productivity and profitability^46^ in pasture-based industries across Australia^47^, North America and Northern China^48,49^. GrassGro® computes soil moisture, pasture production, pasture quality [Crude Protein (%CP) and Dry Matter Digestibility (%DMD)] on a daily basis for each pasture species, paddock and farm. Other variables calculated by the model include sward characteristics, pasture cover, pasture persistence, pasture availability, pasture intake, feed supplement requirements, liveweight change, and feed carry-over effects from year to year. We initialised and parameterized GrassGro® using baseline information collated from each case study farmer.

**High rainfall beef production system**

The beef farm ran a self-replacing cow and calf operations on a land area of 569 ha. This enterprise comprised 367 mature cows calving in late winter (1 Aug with 95% weaning rate, first calving at two years of age) assuming a typical replacement rate of around 20% each year (74 heifers). Home-bred non-replacement heifers and steers were sold at 25 months (1 Sep) at approx. 550 and 600 kg, respectively. An additional 115 of weaners were purchased at 6 months of age (1 Feb) at approx. 200 kg liveweight (LW) and were sold at 25 months (1 Sep) at approx. 600 kg LW. A group of 155 steers was also purchased at 16 months of age (1 Feb) at approx. 375 kg LW each year and sold at 28 months (31 Jan) at approx. 545 kg LW. Before being cast for age on 10 Feb, mature cows were retained for five lactations. Pasture species mainly comprised perennial ryegrass (*Lolium perenne* L.) and white clover (*Trifolium repens* L.) but also cocksfoot (*Dactylis glomerata* L.), subclover (*Trifolium subterraneum* L.) and lucerne (*Medicago sativa*). According to the Northcote classification^50^, the soil type defined in GrassGro was Uc2.3. In addition, 5% of farm area (20 ha lucerne/ryegrass and 8 ha ryegrass/cocksfoot/white clover pastures) was irrigated between 21 Nov and 31 Mar each year (20mm/event on a 14-day interval) to replicate long-term average irrigation water applied. To either maintain LW (cows) or achieve target LWs (all other stock), production feeding rules were implemented in GrassGro using hay (dry matter digestibility (DMD) of 77% and crude protein (CP) of 20%)). While all stock grazed rainfed pastures, home-bred steers were also given access to irrigated pastures throughout the year. Further information can be found in the Supplementary Material (Table S15).

**Low-rainfall sheep production system**

The sheep farm ran a self-replacing Merino superfine wool, prime lamb and, secondary, a beef cattle enterprise grazing 3,170 ha and consisted of 49% native grasslands, 48% rainfed developed pastures and 3% centre pivot irrigation (introduced grasses and legumes). A total of 4,600 ha of native woodlands were also present on the farm that were not subjected to grazing. According to the Northcote classification^50^, the soil type defined in GrassGro was Dy5.61. The modelled rainfed pastures were composed of pure stands of phalaris (*Phalaris aquatica* L.) or phalaris-subclover mixtures. One paddock of lucerne was used for grazing and hay production and another paddock of dual-purpose wheat (*Triticum aestivum* L.) was grazed for four months prior to grain production. Both of these paddocks were irrigated from 1 Sep to 31 Mar with 18 mm of water per application to fill the soil profile to 95% of field capacity when soil water deficit reached 50%.

The sheep farm ran 24,750 animals, grouped in two flocks: a self-replacing Merino flock (SMF) and a prime lamb flock (PLF). The SMF comprised three groups: 5,300 mature superfine Merino ewes, 7,500 wethers and 5,500 replacement ewes and wethers. The SMF ewes were first lambed at 2 years of age and retained for three lambings before entering the PLF for two more annual births before being cast at 7 years old (16 Dec). Before wethers were cast for age (14 Oct), the animals were retained for five years. The non-replacement wether lambs and ewes were sold 1 Feb. A total of 3,450 Merino ewes were mated with White Suffolk rams in the PLF; the 2,950-lamb progeny were sold in mid-December at 27 kg LW. The sheep (except prime lambs) were all shorn on 20 Jul, clean fleece weight (CFW) were 3.3-4.1 kg with fibre diameters of 17.4-18.1µm (variation in CFW and micron depended on stock class and age). Further details are provided on maintenance and production feeding rules, as well as grazing rotations in Supplementary Material (Table S14). The beef cattle herd consisted of 340 mature cows and 60 replacement heifers per age group. Two-year-old mature cows calved (30 Aug) and were retained for eight years before being cast for age. After weaning date (1 Apr), steers (150 head) were sold at 18 months of age (28 Feb at ~ 460 kg LW) while non-replacement heifers (90 head) were sold at 200 kg LW.

**Net farm greenhouse gas emissions**

The Sheep-Beef Greenhouse Accounting Framework (SB-GAF version 1.4^42^), which incorporates Intergovernmental Panel on Climate Change methodology and is detailed in the Australian National Greenhouse Gas Inventory, was used to calculate net farm greenhouse gas emissions. Use of outputs from biophysical models^17,46^ as SB-GAF inputs has been previously shown to be reliable for beef^51^ and sheep enterprises^52^. Twenty-year seasonal mean data from GrassGro was used as input data for SB-GAF. To convert CH_4_ and N_2_O into carbon dioxide equivalents (CO_2_e), SB-GAF assumes 100-year global warming potentials (GWP_100_) of 28 and 265, respectively. Greenhouse gas outputs were calculated as net farm emissions (Mg CO_2_e/annum) and emissions intensity (Mg CO_2_e/Mg product). Greenhouse gas emissions considered included CH_4_ from livestock enteric fermentation and manure; N_2_O from nitrogenous (N) fertiliser, waste management, urinary deposition and indirect N emissions via nitrate leaching and ammonia volatilisation; CO_2_ from synthetic urea applications, electricity and diesel consumption, as well as CO_2_e pre-farm embedded emissions for fertiliser and supplementary feed. Annual electricity and diesel consumption are computed as a function of location, enterprise type, cultivation and machinery use, as well as livestock numbers and use of farm infrastructure. According to Wiedemann et al.^53^, the allocation of emissions between meat and wool was based on protein mass ratio.

**Soil organic carbon in grazed pastures**

The Rothamsted Carbon model (RothC; version 26.3 in Microsoft Excel format^40^) was used to simulate dynamic soil organic carbon (SOC). RothC has been used globally to model the impacts of climate and management on SOC stocks^54^. RothC simulations are driven by historical and projected monthly means of temperature, rainfall and pan evaporation (see *Historical and future climate data*). Monthly average GrassGro outputs were input into RothC including dung and litter. Root residue C inputs were derived from GrassGro outputs considering litter, allocation of net primary production between plant components, active root length density and proportion of root by layer (0-30 cm and 30-100 cm depth) and dung excreted by animals. Further details about the link between GrassGro and RothC can be found in *Supplementary Material* (*see subsection ‘Linking GrassGro and RothC model to account for soil carbon changes in long-term pastures’*). Soil types primarily consisted of clay loam Red Ferrosols on the beef farm^55^, and Dermosols on the slopes adjacent to native vegetation and Vertosols on the river flats on the sheep farm^56^. Soil clay contents in the 0-30 cm and 30-100 cm layers were derived from the TERN-ANU Landscape Data Visualiser (<https://maps.tern.org.au/#/>) and historical SOC was sourced from regional sources^55^. RothC simulates C transfers between several soil organic matter pools, including decomposable plant material (DPM), resistant plant material (RPM), fast and slow microbial biomass (BIOF and BIOS), humified organic matter (HUM) and inert organic matter (IOM) ^40^. RPM, HUM and IOM fractions were comparable to historical data for the three soil types across the two farms^55^. Allocations across SOC pools given by Hoyle et al.^57^ for initial fractions of DPM, BIOF and BIOS were adopted here (1%, 2% and 0.2% of initial SOC stocks, respectively) and IOM fraction was similar to that reported by Falloon et al.^58^. Soil carbon decomposition rates at 30 cm were derived following Jenkinson and Coleman^59^, except for the decomposition rate for RPM, which was set to 0.17 following Richards and Evans^41^, similar to the 0.15 reported by Cotching^55^, such that decomposition rates constants for DPM, RPM, BIO and HUM were 10, 0.17, 0.66 and 0.02, respectively. At 30-100 cm, decomposition rates were calculated following Jenkinson and Coleman^59^; all values were lower than values at 0-30 cm, reflecting lower decomposition rates at depth. Decomposition rates constants for DPM, RPM, BIO and HUM were 0.33, 0.01, 0.02 and 0.00, respectively. To account for the C enrichment of manure by feeding biochar, a sub-model was incorporated to RothC (*see more detail in supplementary materials subsection ‘Accounting for carbon changes in soil by enrichment of manure with biochar’, Fig. S3*).

**Tree growth, carbon in wood and soil carbon beneath tree canopies**

We invoked the FullCAM model (version 4.1.6^41^) to simulate dynamic temporal tree growth, along with carbon sequestration in biomass and in soils beneath trees. FullCAM is currently used in Australia’s National Carbon Accounting System and is driven using mean monthly temperature, rainfall and open-pan evaporation. Soil organic matter and carbon in FullCAM is simulated by RothC; all soil parameters were matched with those we used for RothC described above. FullCAM simulates C cycling between forest and soil components, including litter, surface and subsurface debris. We modelled planting of Tasmanian blue gum (*Eucalyptus globulus* L.) and ‘environmental’ plantings (combination of trees, understory and shrubs native to the region) for the beef and sheep farms, respectively. FullCAM simulations were run continuously from 2022 to 2062 by combining the climate data for the two future time frames, as opposed to two individual simulations commencing 2022 and 2042. We modelled planting of shelter belts for the beef farm and woody thickening of pre-existing woody vegetation for the sheep farm; livestock grazing beneath trees (silvopasture) was not permissible following advice from the RRG. The parameters assumed to simulate SOC changes for grapes were further explained in *Supplementary Material* (*see subsection ‘Diversifying land use with grapes on a sheep farm’*).

**Economic analyses**

In concert with GrassGro outputs, we used the @Risk Software^43^ to stochastically simulate annual feed supply, changes in annual carrying capacity and added annual supplementary feed requirements, commodity prices and animal farm incomes, following approaches outlined in previous studies^60^. Long-term wool, meat and livestock prices adjusted for inflation were adopted from Thomas Elder Markets, Data and Consultancy (<http://thomaseldermarkets.com.au>). The probability distribution of each price variable was derived from analysis of the price data series using BestFit software (Accura Surveys Ltd) (Tables S9, S11-S13). Prices of livestock products were correlated. Economic assessments of the baseline and adaptations were assessed using the @Risk model. To account for economic risk and uncertainty, we performed Monte Carlo simulations using 10,000 iterations of runs of 10-year annual operating profit (Earnings Before Interest and Taxes), as well as measures of return on capital. To attribute a cost for carbon offsetting, we computed operating profit plus a carbon ‘tax’, in which each tonne of CO_2_e was taxed at $60-$100/Mg CO_2_e, following Stiglitz et al.^61^. Any carbon sequestration beyond net farm GHG emissions were ‘credited’ at $35/Mg CO_2_e that of a carbon tax^62^.

**Normalised multidimensional impact assessments**

Normalised multidimensional impact assessments were used to rank all interventions and climate horizons through integration of the relative benefit of each adaptation across economic, biophysical and environmental disciplines into a singular unified metric. Following principles outlined by Gephart et al.^63^, liveweight production, net operating profit (pre-carbon taxes) and net farm GHG emissions were selected for normalisation by the maximum value for each corresponding metric, such that normalised values ranged from 0 to 1. Normalised net farm GHG emissions were computed as the additive inverse of 1 [i.e., 1 - normalised net farm GHG emission factor] given that lower values for this specific metric are desired. Normalised multidimensional impact was calculated as the sum of three key normalised metrics with equal weighting for each metric, such that each normalised output value ranged from 0 (very low impact) to 3 (representing very high beneficial impact in each of the productivity, profitability and GHG emissions dimensions).

**Incremental and contextualised stacking of thematic interventions**

The outcome of the co-design process was distinct adaptation/mitigation themes that were analysed individually or as combined interventions ‘stacked’ together (Table S17; Table 1). The “Low-Hanging Fruit” (LHF) intervention consisted of simple, immediate and reversible changes to existing farm systems that were considered good management practice and may occur over time in the absence of the present study. Incremental adaptations for LHF included changes in animal management/genetics, feedbase management, plant breeding and improved soil fertility (Tables 1 and S17). The second thematic adaptation was co-designed with an overarching aspiration of reducing net farm GHG emissions year on year, such that the trajectory of net farm GHG emissions over time diminished: “Towards Carbon Neutral” or TCN. Incremental adaptations within TCN comprised longer-term, more difficult, higher cost and sometimes irreversible interventions imposed on top of those in LHF including, but not limited to, pasture renovation with deep-rooted genotypes, injecting livestock with an enteric CH_4_ inhibition vaccine and planting regionally appropriate trees on a portion of existing farmland or on newly purchased land. A third thematic adaptation “Income Diversification” (ID) was co-designed with the RRG in which income is derived from sources other than the current livestock farm system through options such as buying another block of land in a different agroclimatic region (climate diversification), leasing land to host a wind turbine farm or diversifying part of the farm area with grapes (climate diversification, reduce the vulnerability to market fluctuations). The fourth thematic adaptation/mitigation bundle, described as “Carbon Neutral” or CN, was created after co-designing pathways designed to reach net zero emissions (Fig. S2). A summary of each adaptation theme together with subset incremental adaptations are shown in Table 1 (further details provided in Tables S15-S18, Fig. S2).

**Table 1. Summarised thematic adaptations co-designed with a Regional Reference Group (RRG).** Each thematic adaptation comprised multiple stacked incremental adaptations suggested by the RRG; the extent to which each factor was varied from the baseline level was derived from feasible values from the literature. Abbreviations: LHF: Low-Hanging Fruit. TCN: Towards Carbon Neutral; this theme also included all incremental adaptations for LHF. ID: Income Diversification. CN. Carbon Neutral Package. SR: Stocking Rate. LW: Liveweight per head. FCE: Feed Conversion Efficiency. RD: Rooting Depth. SSP: Single Superphosphate fertiliser. N: Nitrogen fertiliser. Further details are provided in Tables S15-S18.

| **Theme** | **Incremental, systemic and transformational adaptations stacked into holistic adaptation themes** |
| --- | --- |
| LHF | - Altered lambing/calving dates to better match seasonal pasture supply |
|  | - Altered selling dates/SR/LW to better match seasonal pasture supply |
|  | - Adopting pasture species with 10% improvements in maximum root depth^64^ |
|  | - Increasing soil fertility with SSP and N by 3% [Harrison et al.^65^; all paddocks except the native pastures for the sheep farm] |
|  | - Increasing FCE by 10% in 2030 and 15% in 2050, relative to baseline^66^ |
|  | - Introduction of Talish clover (*Trifolium tumens)* to a proportion of the sheep farm^67^ |
|  | - Removing cattle from the sheep farm and increasing rainfed introduced pasture area to the two sheep flocks |
|  |  |
| TCN | - Strategic manipulation of livestock selling dates/SR/LW to better match seasonal pasture supply |
|  | - Pasture renovation with (and increased farm area of) lucerne pastures |
|  | - Injecting animals with an enteric CH_4_ inhibitor vaccine to reduce CH_4_ by 30%^30^ - Purchase 50 ha of land for the beef farm to establish a tree plantation of Tasmanian Blue Gums to offset livestock GHG emissions |
|  | - Thickening of 200 ha of existing nature pasture (non-grazed) land for sheep farm with environmental plantings (trees, shrubs and understory species endemic to the region) |
| ID | - Buying an extra farm in a different agroclimatic region (by translocating cow calf systems to Gladstone, NE Tasmania to dedicate the current farm for backgrounding and finishing of weaners) - Diversifying land use with grapes by repurposing 30ha from the sheep farm to grow Pinot Noir and Chardonnay grapes (processed offsite and outside scope of the current project) - Hosting a wind farm (by leasing land for 12 wind turbines to generate an extra income, no insetting of CO_2_ from turbines to reduce on-farm GHG emissions, in line with the business model of the wind turbine company) |
| CN | - Feeding red seaweed (*Asparagopsis taxiformis*) to offset CH_4_ by 80%^68^ - Pasture renovation with (and increased farm area of) lucerne pastures - Purchase 55 to 85 ha of land for the beef farm to establish a tree plantation of Tasmanian Blue Gums to offset livestock GHG emissions - Thickening of 200 to 220 ha of existing nature pasture (non-grazed) land for sheep farm with environmental plantings (trees, shrubs and understory species endemic to the region) - Transformational increase in FCE, to 20% in 2030 and 30% in 2050, relative to baseline^66^ |

**References**

1 *FAO. The Impact of Disasters and Crises on Agriculture and Food Security (2021).* (Food and Agriculture Organization of the United Nations Rome, Italy).

2 Baker, H. S. *et al.* Higher CO2 concentrations increase extreme event risk in a 1.5 °C world. *Nature Climate Change* **8**, 604-608 (2018). <https://doi.org:10.1038/s41558-018-0190-1>

3 IPCC, 2021: Summary for Policymakers. In: Climate Change 2021: The Physical Science Basis. Contribution of Working Group I to the Sixth Assessment Report of the Intergovernmental Panel on Climate Change [Masson-Delmotte, V., P. Zhai, A. Pirani, S.L. Connors, C. Péan, S. Berger, N. Caud, Y. Chen, L. Goldfarb, M.I. Gomis, M. Huang, K. Leitzell, E. Lonnoy, J.B.R. Matthews, T.K. Maycock, T. Waterfield, O. Yelekçi, R. Yu, and B. Zhou (eds.)].(2021).

4 UN. Climate Change Conference in Glasgow (COP26). The Glasgow Climate Pact. . (2021).

5 Salman, R., Ferdinand, T., Choularton, R. & Carter, R. Transformative Adaptation in Livestock Production Systems. (2019).

6 Australian Government. Clean Energy Regulator. Emissions Reduction Fund. Understanding your integrated farm management project. Emissions Reduction Fund simple method guide for integrated farm management projects registered under the Carbon Credits (Carbon Farming Initiative-Integrated Farm Management). (2022).

7 Harrison, M. T. *et al.* Carbon myopia: The urgent need for integrated social, economic and environmental action in the livestock sector. *Global Change Biology* **27**, 5726-5761 (2021). <https://doi.org:https://doi.org/10.1111/gcb.15816>

8 Bilotto, F., Christie, K. M., Malcolm, B. & Harrison, M. T. Carbon, cash, cattle and the climate crisis. *Sustainability Science* (2022).

9 MLA. State of the industry report. The Australian red meat and livestock industry. (2022).

10 Parliament of Australia. Australian Meat and Live-stock Industry Act 1997. Chapter 5: Red Meat Memorandum of Understanding. (1997).

11 Primary Industries Climate Challenges Centre (PICCC). NEXUS project: exploring profitable, sustainable livestock businesses in an increasingly variable climate. Retrievable at: <https://piccc.org.au/research/project/NEXUS.html>. (2023).

12 UNFCCC. Sharm el-Sheikh Implementation Plan. The 27th United Nations Climate Change Conference or Conference of the Parties of the United Nations Framework Convention on Climate Change, COP27. Egypt. (2022).

13 United Nations Environment Programme. Adaptation Gap Report 2022: Too Little, Too Slow - Climate adaptation failure puts world at risk. Nairobi: United Nations Environment Programme. Retrievable at: <https://www.unep.org/resources/adaptation-gap-report-2022>. . (2022).

14 Reed, J. *et al.* Co-producing theory of change to operationalize integrated landscape approaches. *Sustainability Science* (2022). <https://doi.org:10.1007/s11625-022-01190-3>

15 Jones, J. W. *et al.* Brief history of agricultural systems modeling. *Agricultural Systems* **155**, 240-254 (2017). <https://doi.org:https://doi.org/10.1016/j.agsy.2016.05.014>

16 Fuso Nerini, F. *et al.* Connecting climate action with other Sustainable Development Goals. *Nature Sustainability* **2**, 674-680 (2019). <https://doi.org:10.1038/s41893-019-0334-y>

17 Harrison, M. T., Cullen, B. R. & Rawnsley, R. P. Modelling the sensitivity of agricultural systems to climate change and extreme climatic events. *Agricultural Systems* **148**, 135-148 (2016). <https://doi.org:https://doi.org/10.1016/j.agsy.2016.07.006>

18 Vijn, S. *et al.* Key Considerations for the Use of Seaweed to Reduce Enteric Methane Emissions From Cattle. *Frontiers in Veterinary Science* **7** (2020). <https://doi.org:10.3389/fvets.2020.597430>

19 Kelly, J. Australian Seaweed Industry Blueprint. A blue print for frowth. Australian Seaweed Institute. (2020).

20 Glasson, C. R. K. *et al.* Benefits and risks of including the bromoform containing seaweed Asparagopsis in feed for the reduction of methane production from ruminants. *Algal Research* **64**, 102673 (2022). <https://doi.org:https://doi.org/10.1016/j.algal.2022.102673>

21 Van Beem, D., Wellington, D., Paganoni, B. L., Vercoe, P. E. & Milton, J. T. B. Feed efficiency for meat and wool production by Merino and F1 DohneMerino lambs fed pelleted diets of different nutritive value. *Australian Journal of Experimental Agriculture* **48**, 879-884 (2008). <https://doi.org:https://doi.org/10.1071/EA08063>

22 Arthur, P. F. & Herd, R. M. Efficiency of feed utilisation by livestock — Implications and benefits of genetic improvement. *Canadian Journal of Animal Science* **85**, 281-290 (2005). <https://doi.org:10.4141/a04-062>

23 Alford, A. R. *et al.* The impact of breeding to reduce residual feed intake on enteric methane emissions from the Australian beef industry. *Australian Journal of Experimental Agriculture* **46**, 813-820 (2006). <https://doi.org:https://doi.org/10.1071/EA05300>

24 Sturludóttir, E. *et al.* Benefits of mixing grasses and legumes for herbage yield and nutritive value in Northern Europe and Canada. *Grass and Forage Science* **69**, 229-240 (2014). <https://doi.org:https://doi.org/10.1111/gfs.12037>

25 Suter, M. *et al.* Nitrogen yield advantage from grass–legume mixtures is robust over a wide range of legume proportions and environmental conditions. *Global Change Biology* **21**, 2424-2438 (2015). <https://doi.org:https://doi.org/10.1111/gcb.12880>

26 Wang, X. *et al.* Grazing improves C and N cycling in the Northern Great Plains: a meta-analysis. *Scientific Reports* **6**, 33190 (2016). <https://doi.org:10.1038/srep33190>

27 Peixoto, L. *et al.* Deep-rooted perennial crops differ in capacity to stabilize C inputs in deep soil layers. *Scientific Reports* **12**, 5952 (2022). <https://doi.org:10.1038/s41598-022-09737-1>

28 Hancock, K. *et al.* Progress towards developing bloat-safe legumes for the farming industry. *Crop and Pasture Science* **65**, 1107-1113 (2014). <https://doi.org:https://doi.org/10.1071/CP13308>

29 Vermeulen, S. *et al.* A global agenda for collective action on soil carbon. *Nature Sustainability* **2**, 2-4 (2019). <https://doi.org:10.1038/s41893-018-0212-z>

30 Reisinger, A. *et al.* How necessary and feasible are reductions of methane emissions from livestock to support stringent temperature goals? *Philosophical Transactions of the Royal Society A: Mathematical, Physical and Engineering Sciences* **379**, 20200452 (2021). <https://doi.org:doi:10.1098/rsta.2020.0452>

31 Yu, G., Beauchemin, K. A. & Dong, R. A Review of 3-Nitrooxypropanol for Enteric Methane Mitigation from Ruminant Livestock. *Animals* **11**, 3540 (2021).

32 Fujimori, S. *et al.* Land-based climate change mitigation measures can affect agricultural markets and food security. *Nature Food* **3**, 110-121 (2022). <https://doi.org:10.1038/s43016-022-00464-4>

33 Giller, K. E. *et al.* The future of farming: Who will produce our food? *Food Security* **13**, 1073-1099 (2021). <https://doi.org:10.1007/s12571-021-01184-6>

34 Wise, L. *et al.* Optimizing sequestered carbon in forest offset programs: balancing accounting stringency and participation. *Carbon Balance and Management* **14**, 16 (2019). <https://doi.org:10.1186/s13021-019-0131-y>

35 Hong, C. *et al.* Land-use emissions embodied in international trade. *Science* **376**, 597-603 (2022). <https://doi.org:doi:10.1126/science.abj1572>

36 Stevanović, M. *et al.* Mitigation Strategies for Greenhouse Gas Emissions from Agriculture and Land-Use Change: Consequences for Food Prices. *Environmental Science & Technology* **51**, 365-374 (2017). <https://doi.org:10.1021/acs.est.6b04291>

37 Fedele, G., Donatti, C. I., Harvey, C. A., Hannah, L. & Hole, D. G. Transformative adaptation to climate change for sustainable social-ecological systems. *Environmental Science & Policy* **101**, 116-125 (2019). <https://doi.org:https://doi.org/10.1016/j.envsci.2019.07.001>

38 de Coninck, H. *et al.* in *Global warming of 1.5 C: Summary for policy makers* 313-443 (IPCC-The Intergovernmental Panel on Climate Change, 2018).

39 Moore, A. D., Donnelly, J. R. & Freer, M. GRAZPLAN: Decision support systems for Australian grazing enterprises. III. Pasture growth and soil moisture submodels, and the GrassGro DSS. *Agricultural Systems* **55**, 535-582 (1997). <https://doi.org:https://doi.org/10.1016/S0308-521X(97)00023-1>

40 Coleman, K. & Jenkinson, D. RothC: a model for the turnover of soil carbon model description and user guide. *Rothamsted Research, Harpenden, UK* (2014).

41 Richards, G. P. & Evans, D. M. W. Development of a carbon accounting model (FullCAM Vers. 1.0) for the Australian continent. *Australian Forestry* **67**, 277-283 (2004). <https://doi.org:10.1080/00049158.2004.10674947>

42 Dunn, J., Wiedemann, S. & Eckard, R. J. A Greenhouse Accounting Framework for Beef and Sheep properties based on the Australian National Greenhouse Gas Inventory methodology. Retrieved from <http://piccc.org.au/Tools>. (2020).

43 Corporation, P. 'The DecisionTools Suite version 5.' (Palisade Corporation: Ithaca, USA). (2012).

44 Harris, R. M. B., Love, P. T., Fox-Hughes, P., Remenyi, T. A. & L., B. N. An assessment of the viability of prescribed burning as a management tool under a changing climate - Stage 2, Technical Report, Antarctic Climate and Ecosystems Cooperative Research Centre, Hobart, Tasmania. (2019).

45 CCIA. Climate Change in Australia. Climate information, projections, tools and data. Available at available at <https://www.climatechangeinaustralia.gov.au/en/> (accesed 14 Feb 2022). (2020).

46 Harrison, M. T. *et al.* The concordance between greenhouse gas emissions, livestock production and profitability of extensive beef farming systems. *Animal Production Science* **56**, 370-384 (2016). <https://doi.org:https://doi.org/10.1071/AN15515>

47 Cullen, B. R., Eckard, R. J., Timms, M. & Phelps, D. G. The effect of earlier mating and improving fertility on greenhouse gas emissions intensity of beef production in northern Australian herds. *The Rangeland Journal* **38**, 283-290 (2016). <https://doi.org:https://doi.org/10.1071/RJ15063>

48 Duan, Q. *et al.* in *Computer and Computing Technologies in Agriculture IV.* (eds Daoliang Li, Yande Liu, & Yingyi Chen) 134-146 (Springer Berlin Heidelberg).

49 Lynch, D. H., Cohen, R. D. H., Fredeen, A., Patterson, G. & Martin, R. C. Management of Canadian prairie region grazed grasslands: Soil C sequestration, livestock productivity and profitability. *Canadian Journal of Soil Science* **85**, 183-192 (2005). <https://doi.org:10.4141/s04-053>

50 Northcote, K. A Factual Key for the Recognition of Australian Soils. 4th Edition, Rellim Technical Publishers, Glenside, South Australia. (1979).

51 Herd, R. M., Oddy, V. H. & Bray, S. Baseline and greenhouse-gas emissions in extensive livestock enterprises, with a case study of feeding lipid to beef cattle. *Animal Production Science* **55**, 159-165 (2015). <https://doi.org:https://doi.org/10.1071/AN14222>

52 Harrison, M. T. *et al.* Increasing ewe genetic fecundity improves whole-farm production and reduces greenhouse gas emissions intensities: 1. Sheep production and emissions intensities. *Agricultural Systems* **131**, 23-33 (2014). <https://doi.org:https://doi.org/10.1016/j.agsy.2014.07.008>

53 Wiedemann, S. G. *et al.* Application of life cycle assessment to sheep production systems: investigating co-production of wool and meat using case studies from major global producers. *The International Journal of Life Cycle Assessment* **20**, 463-476 (2015). <https://doi.org:10.1007/s11367-015-0849-z>

54 Morais, T. G., Teixeira, R. F. M. & Domingos, T. Detailed global modelling of soil organic carbon in cropland, grassland and forest soils. *PloS one* **14**, e0222604-e0222604 (2019). <https://doi.org:10.1371/journal.pone.0222604>

55 Cotching, W. E. Organic matter in the agricultural soils of Tasmania, Australia – A review. *Geoderma* **312**, 170-182 (2018). <https://doi.org:https://doi.org/10.1016/j.geoderma.2017.10.006>

56 Smith, R. W. *et al.* Effects of wildlife grazing on the production, ground cover and plant species composition of an established perennial pasture in the Midlands region, Tasmania. *Wildlife Research* **39**, 123-136 (2012). <https://doi.org:https://doi.org/10.1071/WR11032>

57 Hoyle, F. C., D'Antuono, M., Overheu, T. & Murphy, D. V. Capacity for increasing soil organic carbon stocks in dryland agricultural systems. *Soil Research* **51**, 657-667 (2013). <https://doi.org:https://doi.org/10.1071/SR12373>

58 Falloon, P., Smith, P., Coleman, K. & Marshall, S. Estimating the size of the inert organic matter pool from total soil organic carbon content for use in the Rothamsted carbon model. *Soil Biology and Biochemistry* **30**, 1207-1211 (1998). <https://doi.org:https://doi.org/10.1016/S0038-0717(97)00256-3>

59 Jenkinson, D. S. & Coleman, K. The turnover of organic carbon in subsoils. Part 2. Modelling carbon turnover. *European Journal of Soil Science* **59**, 400-413 (2008). <https://doi.org:https://doi.org/10.1111/j.1365-2389.2008.01026.x>

60 Bell, L. W., Harrison, M. T. & Kirkegaard, J. A. Dual-purpose cropping – capitalising on potential grain crop grazing to enhance mixed-farming profitability. *Crop and Pasture Science* **66**, i-iv (2015). <https://doi.org:https://doi.org/10.1071/CPv66n4_FO>

61 Stiglitz, J. E. *et al.* Report of the high-level commission on carbon prices. (2017).

62 Zhang, Y., Mounter, S. & Griffith, G. Updating and recalibrating equilibrium displacement models of the Australian livestock industries: beef. *Australasian Agribusiness Review* **26**, 48-67 (2018).

63 Gephart, J. A. *et al.* The environmental cost of subsistence: Optimizing diets to minimize footprints. *Science of The Total Environment* **553**, 120-127 (2016). <https://doi.org:https://doi.org/10.1016/j.scitotenv.2016.02.050>

64 Cullen, B. R., Rawnsley, R. P., Eckard, R. J., Christie, K. M. & Bell, M. J. Use of modelling to identify perennial ryegrass plant traits for future warmer and drier climates. *Crop and Pasture Science* **65**, 758-766 (2014). <https://doi.org:https://doi.org/10.1071/CP13408>

65 Harrison, M. T., Christie, K. M., Rawnsley, R. P. & Eckard, R. J. Modelling pasture management and livestock genotype interventions to improve whole-farm productivity and reduce greenhouse gas emissions intensities. *Animal Production Science* **54**, 2018-2028 (2014). <https://doi.org:https://doi.org/10.1071/AN14421>

66 Alcock, D. J. & Hegarty, R. S. Potential effects of animal management and genetic improvement on enteric methane emissions, emissions intensity and productivity of sheep enterprises at Cowra, Australia. *Animal Feed Science and Technology* **166-167**, 749-760 (2011). <https://doi.org:https://doi.org/10.1016/j.anifeedsci.2011.04.053>

67 Hayes, R. C. *et al.* Prospects for improving perennial legume persistence in mixed grazed pastures of south-eastern Australia, with particular reference to white clover. *Crop and Pasture Science* **70**, 1141-1162, 1122 (2019).

68 Wasson, D. E., Yarish, C. & Hristov, A. N. Enteric methane mitigation through Asparagopsis taxiformis supplementation and potential algal alternatives. *Frontiers in Animal Science* **3** (2022). <https://doi.org:10.3389/fanim.2022.999338>
