# Supplementary material for: Costs of transitioning the livestock sector to net-zero emissions under future climates
Source: Nat Commun. 2025 Apr 23;16:3810. doi: 10.1038/s41467-025-59203-5 (PMC12019546; doi:10.1038/s41467-025-59203-5)
Supplement: Supplementary file 4 — Source Data [file 41467_2025_59203_MOESM4_ESM.zip › Source data file SUBMITTED 20 March 2025/Bilotto et al (2025) Figure 6 SUBMITTED 20 March 2025.pdf]

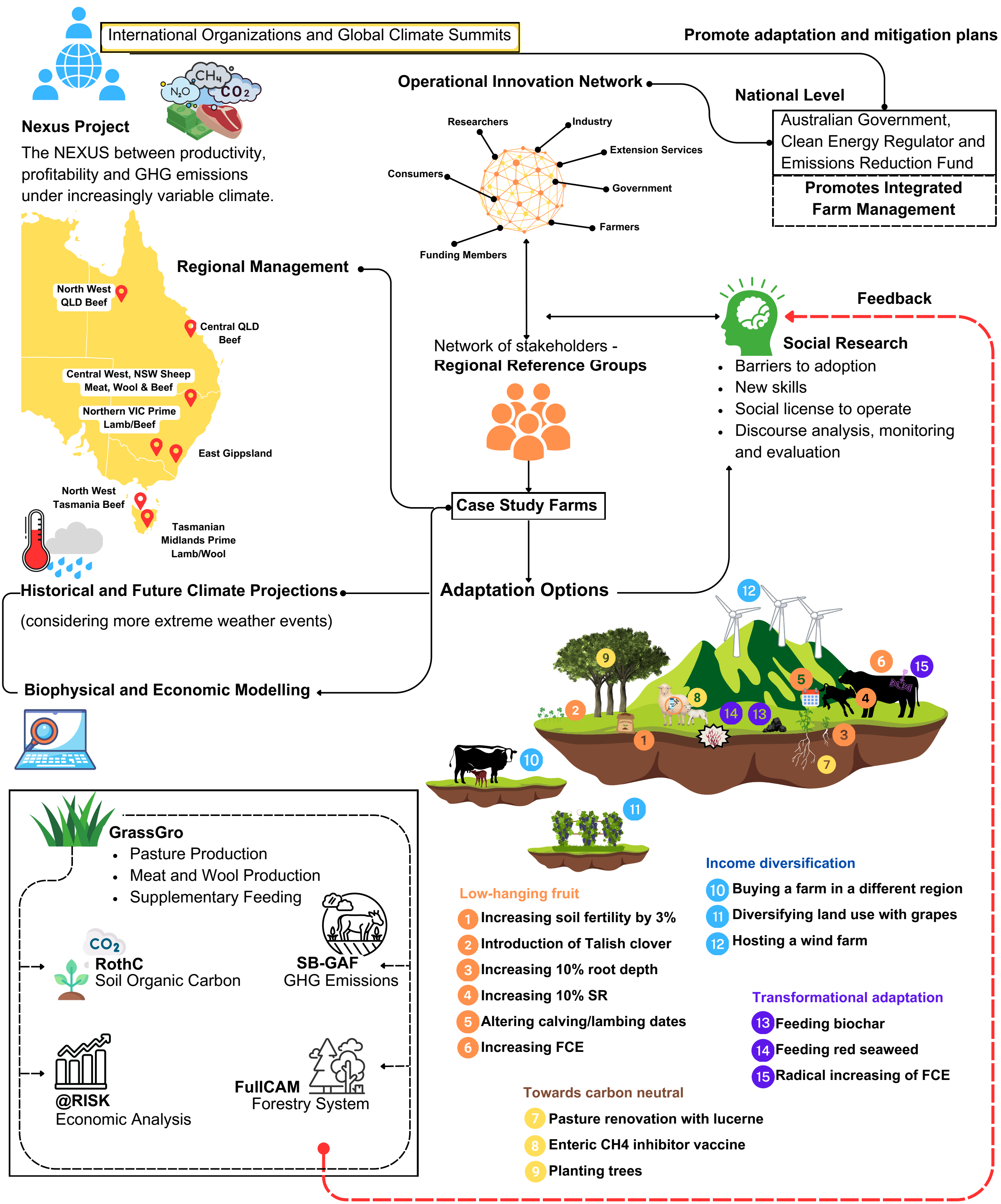
International Organizations and Global Climate Summits
Promote adaptation and mitigation plans
CH4
N2O
CO2
Nexus Project
The NEXUS between productivity, profitability and GHG emissions under increasingly variable climate.
Operational Innovation Network
National Level
Australian Government, Clean Energy Regulator and Emissions Reduction Fund
Promotes Integrated Farm Management
Researchers
Industry
Extension Services
Consumers
Government
Farmers
Funding Members
Regional Management
North West QLD Beef
Central QLD Beef
Central West, NSW Sheep Meat, Wool & Beef
Northern VIC Prime Lamb/Beef
East Gippsland
North West Tasmania Beef
Tasmanian Midlands Prime Lamb/Wool
Feedback
Social Research
Barriers to adoption
New skills
Social license to operate
Discourse analysis, monitoring and evaluation
Network of stakeholders -
Regional Reference Groups
Case Study Farms
Historical and Future Climate Projections
(considering more extreme weather events)
Adaptation Options
Biophysical and Economic Modelling
GrassGro
Pasture Production
Meat and Wool Production
Supplementary Feeding
CO2
RothC
Soil Organic Carbon
SB-GAF
GHG Emissions
@RISK
Economic Analysis
FullCAM
Forestry System
Low-hanging fruit
1 Increasing soil fertility by 3%
2 Introduction of Talish clover
3 Increasing 10% root depth
4 Increasing 10% SR
5 Altering calving/lambing dates
6 Increasing FCE
Income diversification
10 Buying a farm in a different region
11 Diversifying land use with grapes
12 Hosting a wind farm
Transformational adaptation
13 Feeding biochar
14 Feeding red seaweed
15 Radical increasing of FCE
Towards carbon neutral
7 Pasture renovation with lucerne
8 Enteric CH4 inhibitor vaccine
9 Planting trees
