# Supplementary figures and images for: Costs of transitioning the livestock sector to net-zero emissions under future climates
Source: Nat Commun. 2025 Apr 23;16:3810. doi: 10.1038/s41467-025-59203-5 (PMC12019546; doi:10.1038/s41467-025-59203-5)

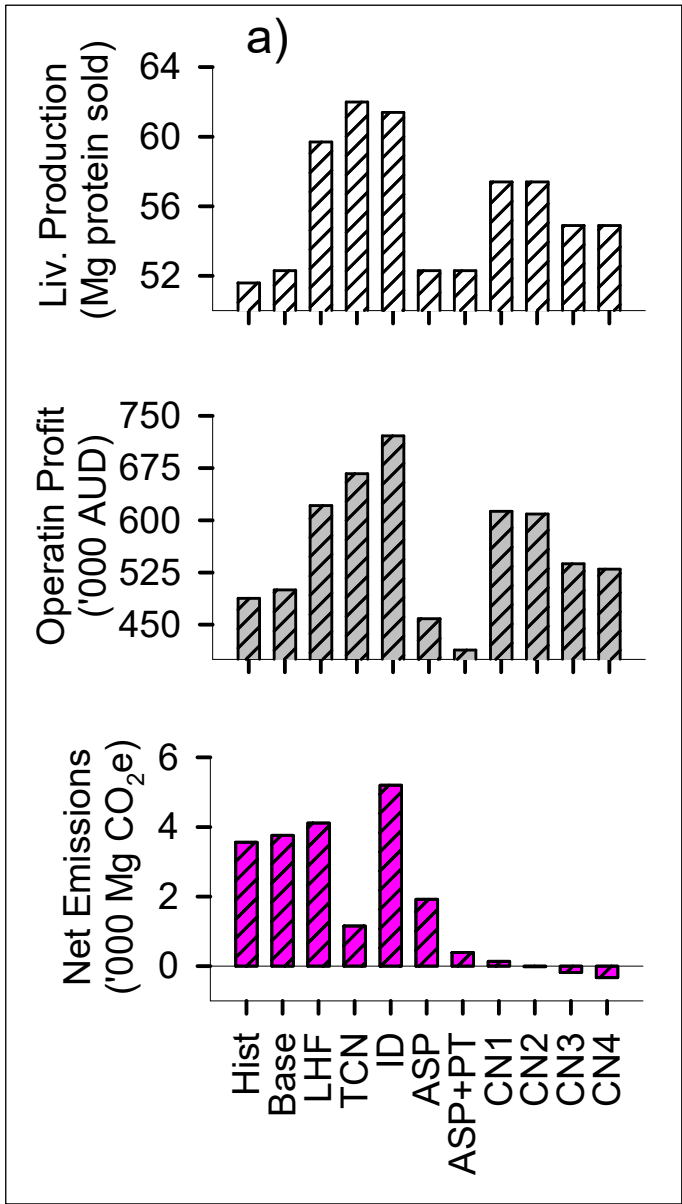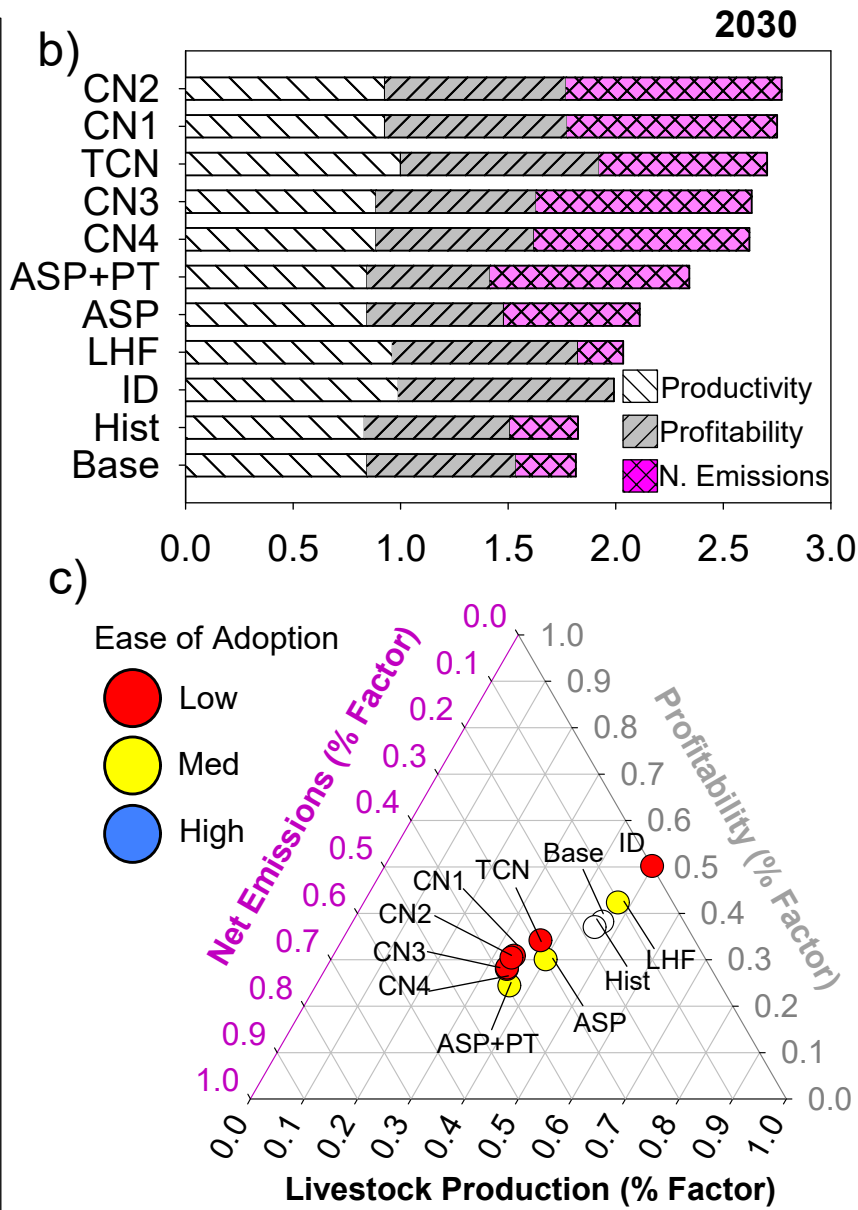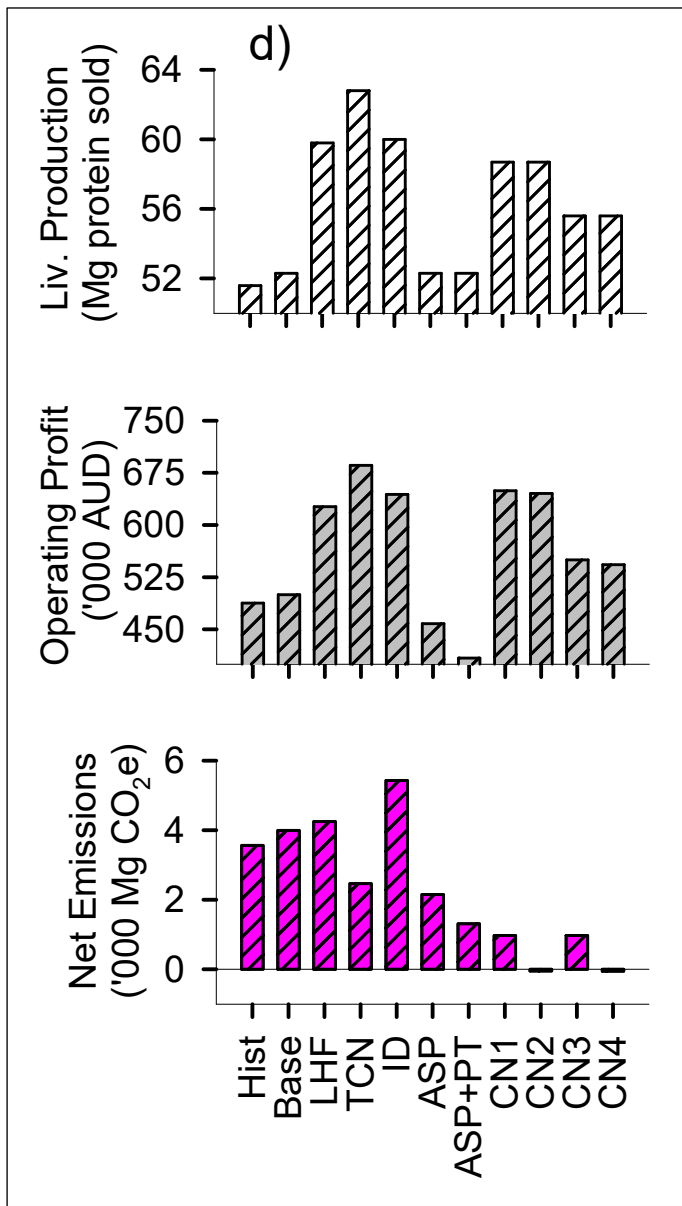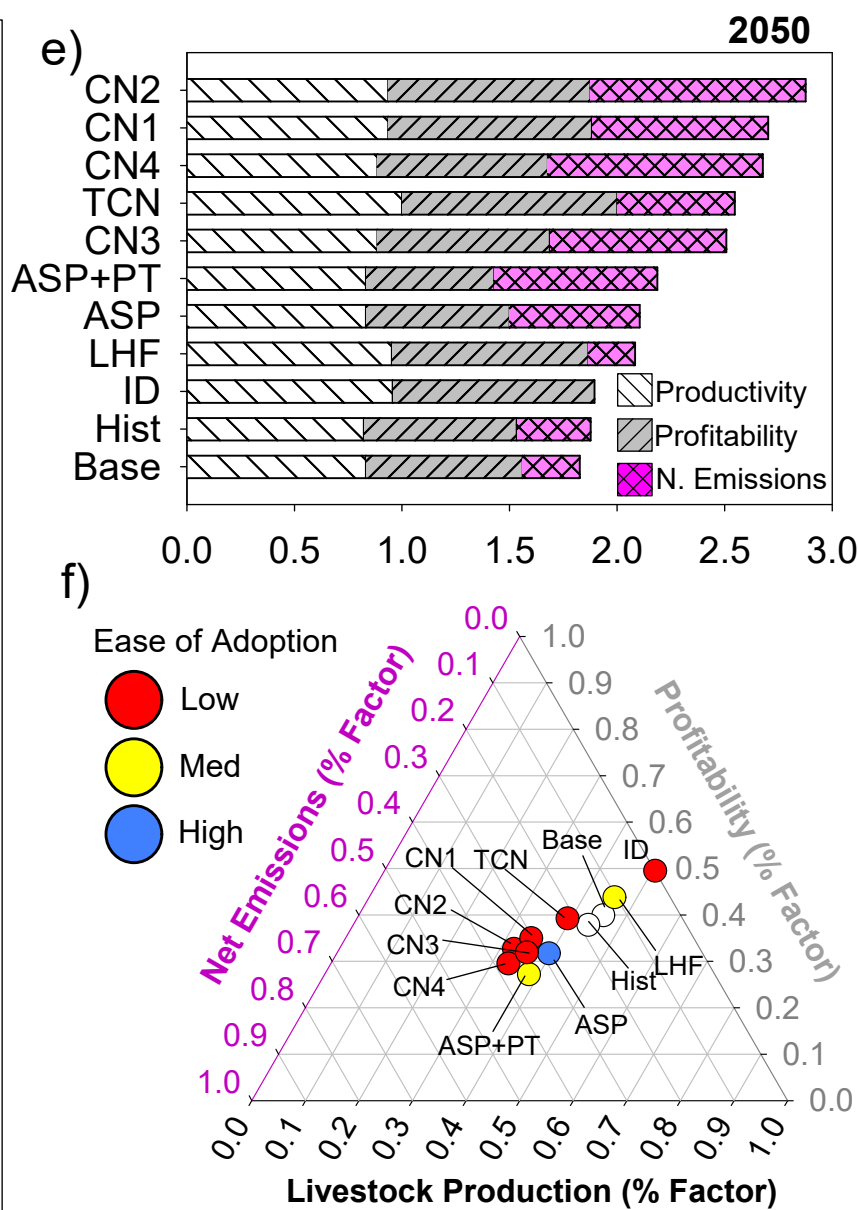

Supplement: Supplementary file 4 — Source Data [file 41467_2025_59203_MOESM4_ESM.zip › Source data file SUBMITTED 20 March 2025/Bilotto et al (2025) Fig 3a, b, c, d, e, f SUBMITTED 20 March 2025.PDF]

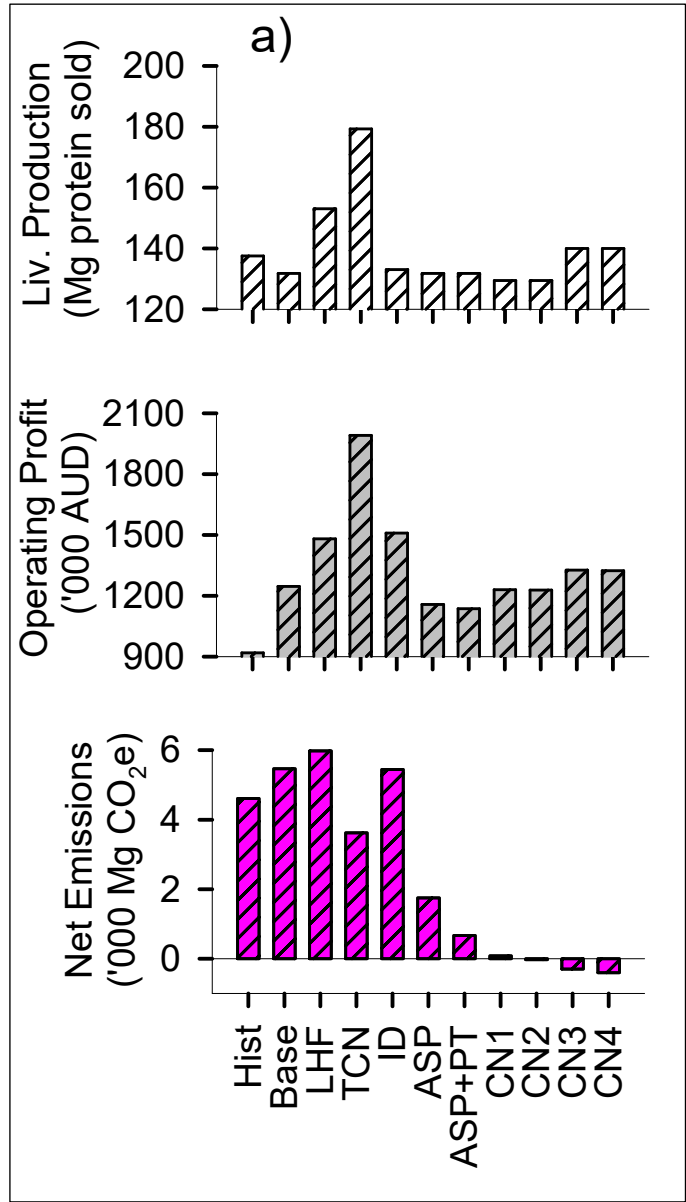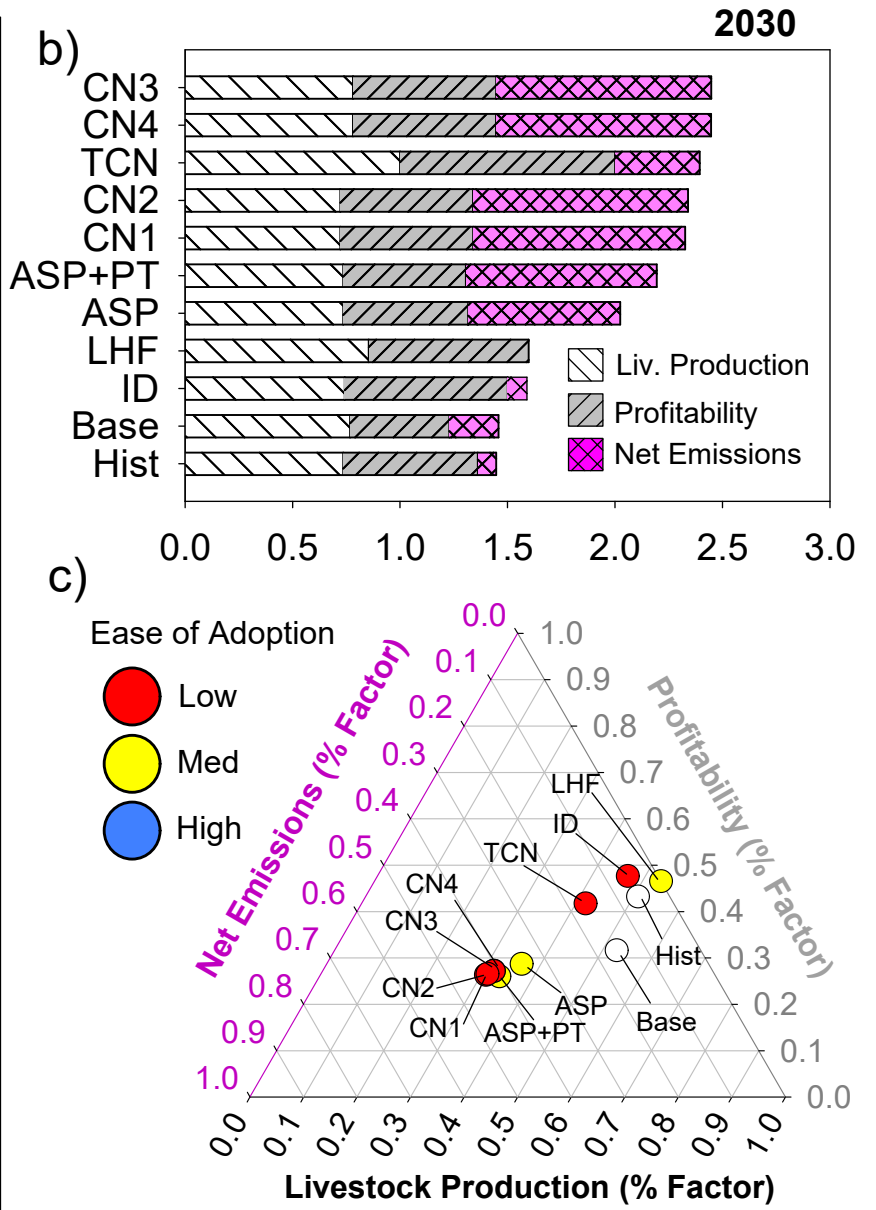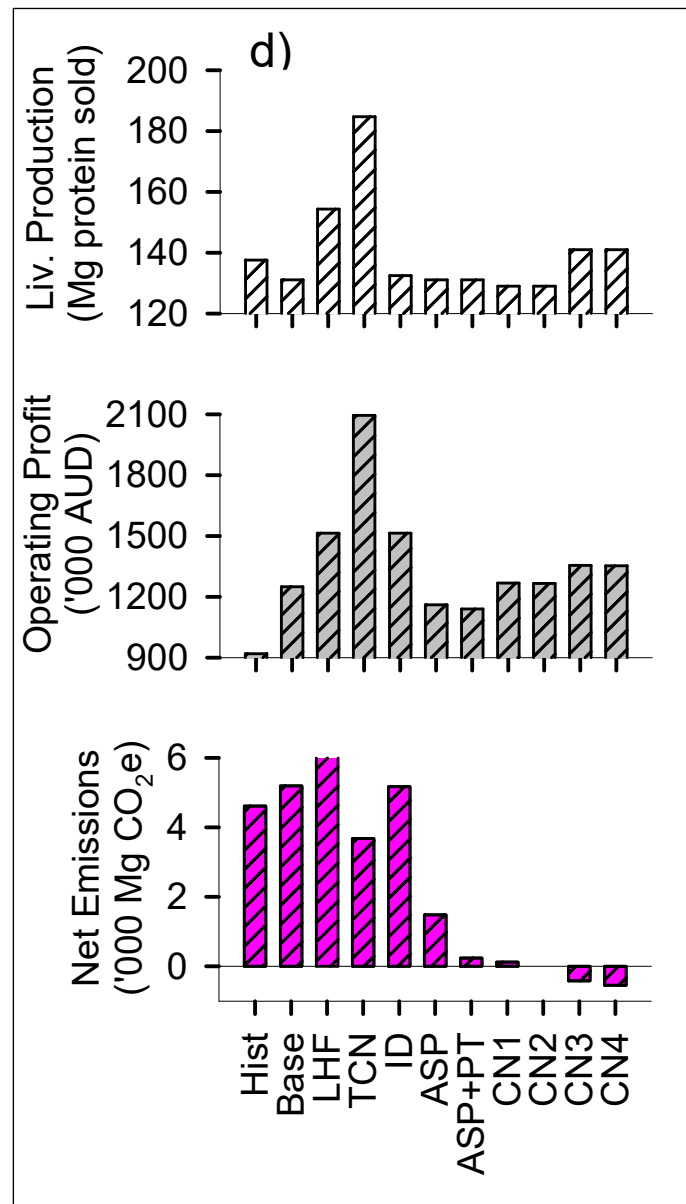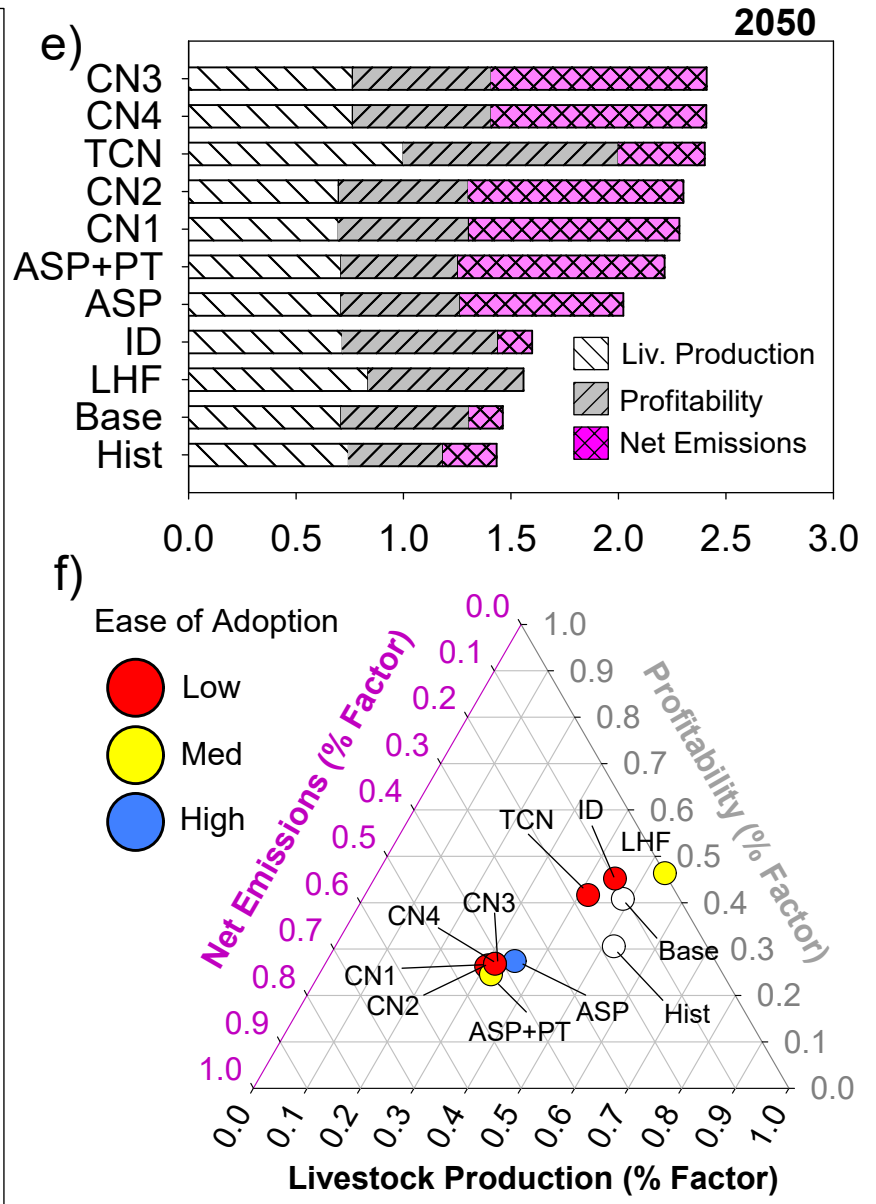

Supplement: Supplementary file 4 — Source Data [file 41467_2025_59203_MOESM4_ESM.zip › Source data file SUBMITTED 20 March 2025/Bilotto et al (2025) Fig 4a, b, c, d, e, f SUBMITTED 20 March 2025.PDF]

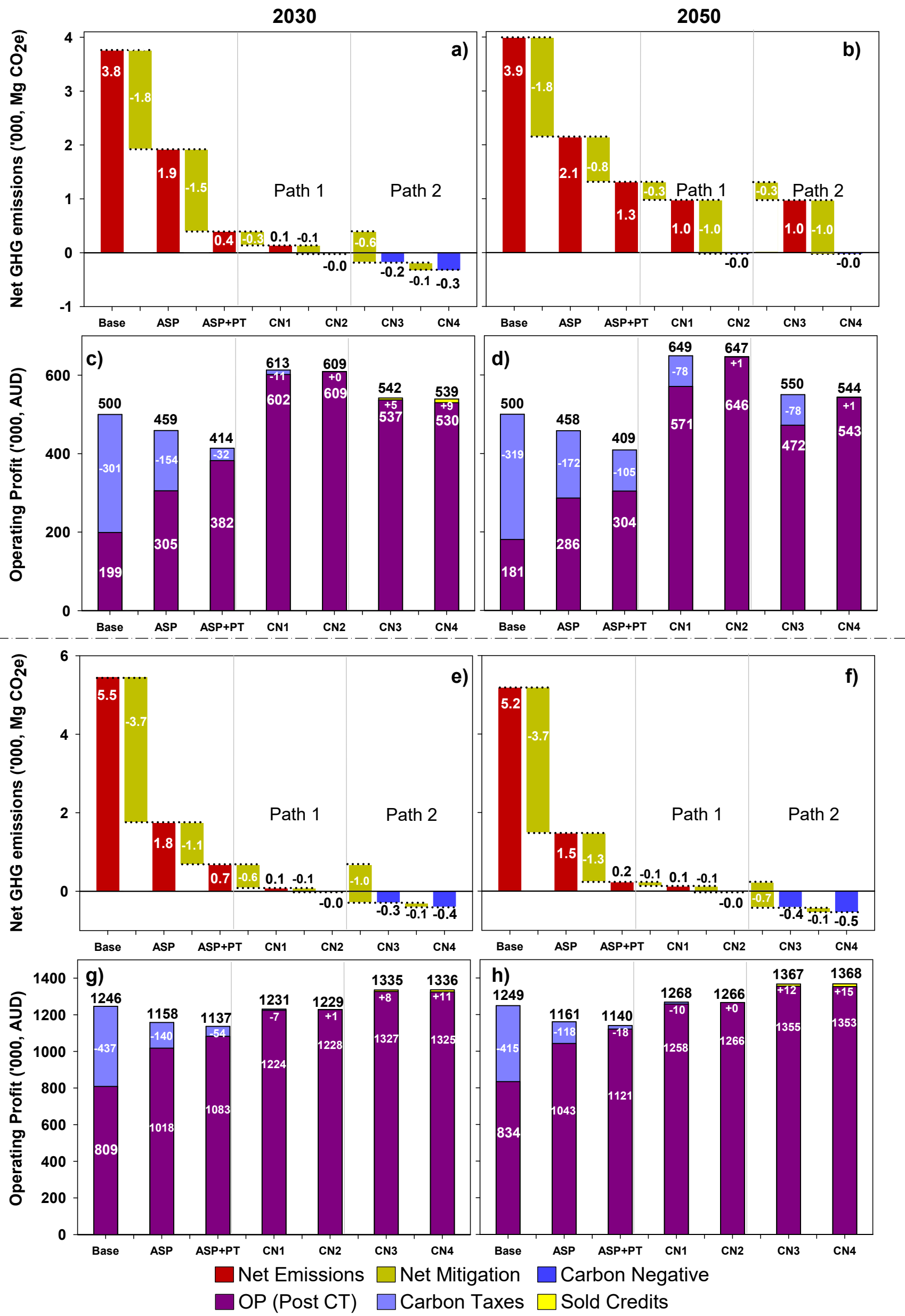

Supplement: Supplementary file 4 — Source Data [file 41467_2025_59203_MOESM4_ESM.zip › Source data file SUBMITTED 20 March 2025/Bilotto et al (2025) Fig 5a, b, c, d, e, f, g, h SUBMITTED 20 March 2025.PDF]

## Slide 1
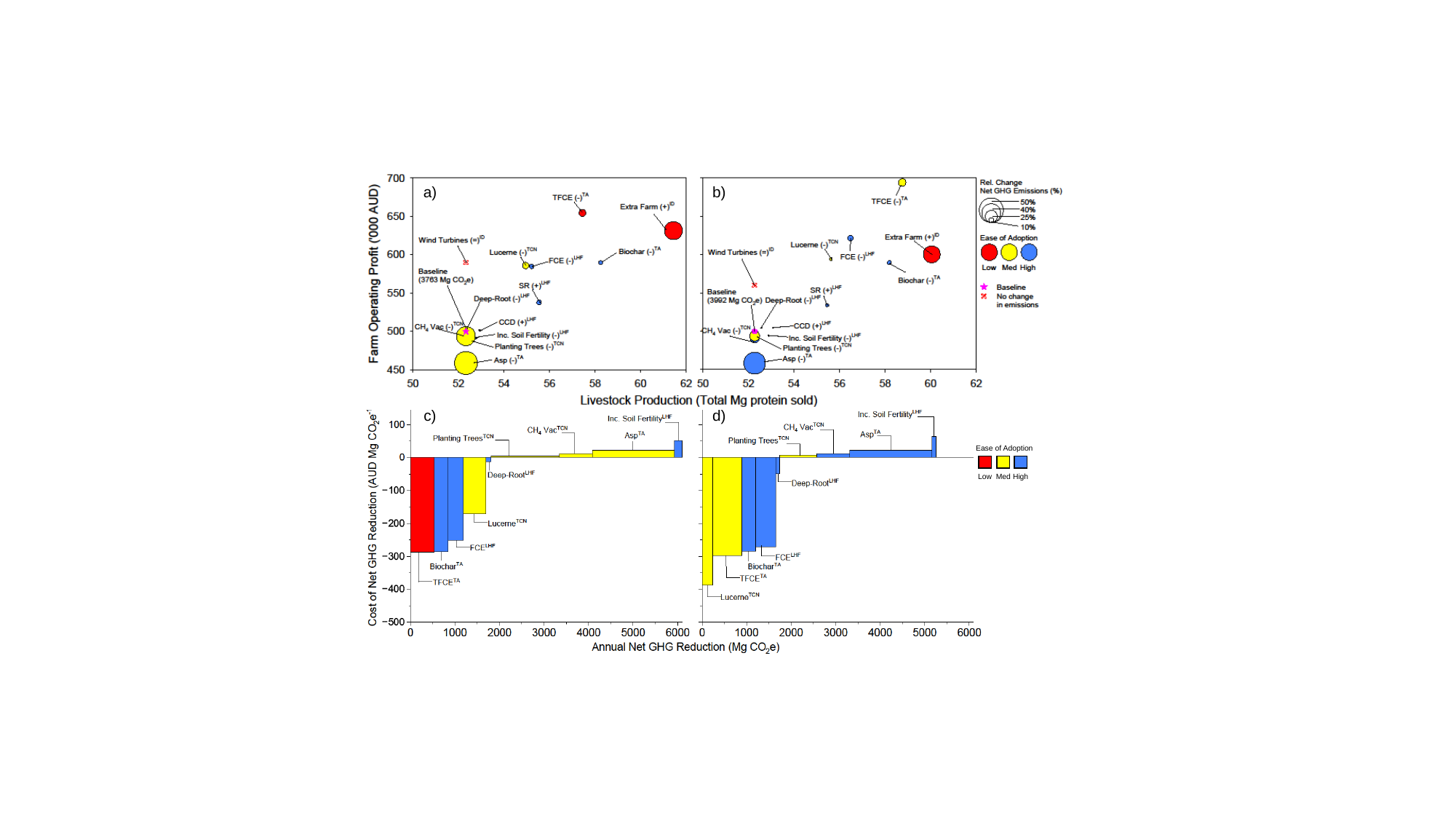

a)
b)
c)
d)
Ease of Adoption
Low
Med
High

Supplement: Supplementary file 4 — Source Data [file 41467_2025_59203_MOESM4_ESM.zip › Source data file SUBMITTED 20 March 2025/Bilotto et al (2025) Figure 1 SUBMITTED 20 March 2025.pptx]

## Slide 1
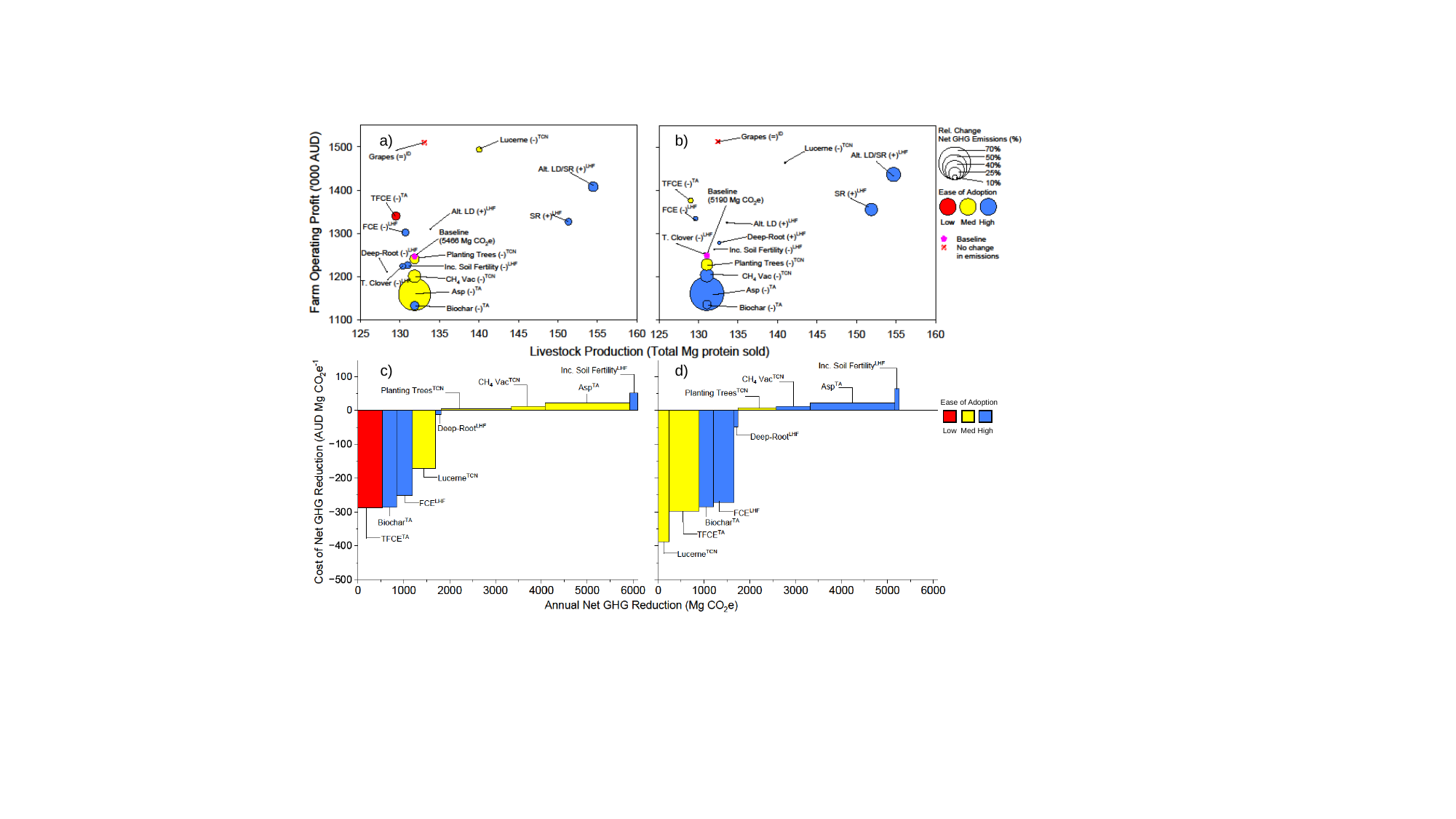

b)
a)
c)
d)
Ease of Adoption
Low
Med
High

Supplement: Supplementary file 4 — Source Data [file 41467_2025_59203_MOESM4_ESM.zip › Source data file SUBMITTED 20 March 2025/Bilotto et al (2025) Figure 2 SUBMITTED 20 March 2025.pptx]

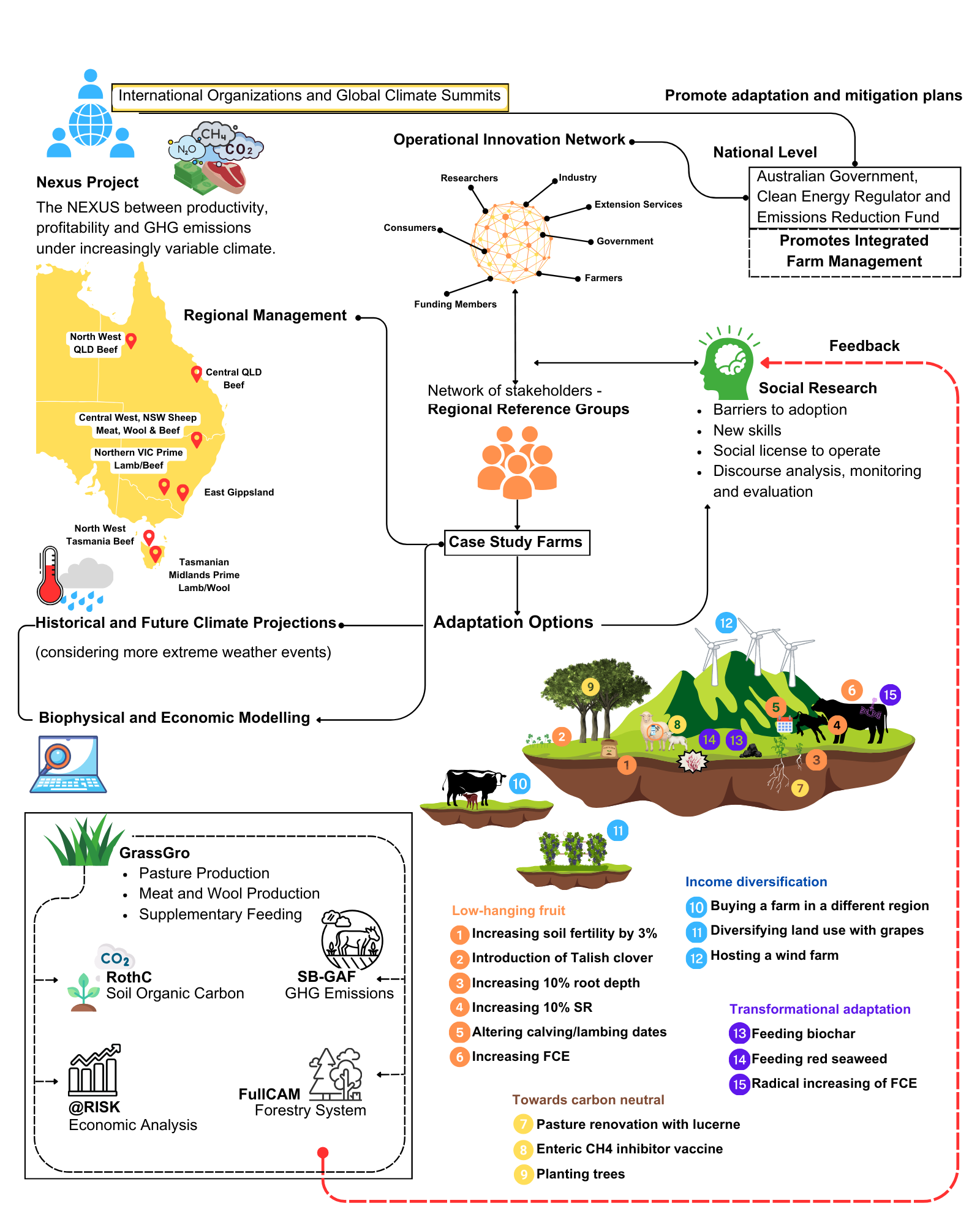

Supplement: Supplementary file 4 — Source Data [file 41467_2025_59203_MOESM4_ESM.zip › Source data file SUBMITTED 20 March 2025/Bilotto et al (2025) Figure 6 SUBMITTED 20 March 2025.png]
